# Nanoengineering of Tin Monosulfide (SnS)‐Based Structures for Emerging Applications

**DOI:** 10.1002/smsc.202100098

**Published:** 2021-12-29

**Authors:** You Zi, Jun Zhu, Lanping Hu, Mengke Wang, Weichun Huang

**Affiliations:** ^1^ School of Chemistry and Chemical Engineering Nantong University Nantong Jiangsu 226019 P. R. China

**Keywords:** black phosphorus analogues, high-performance energy devices, nanoengineering, nanostructures, tin monosulfide

## Abstract

In recent years, tin monosulfide (SnS), one kind of black phosphorus analogues, is of great interest owing to its unique properties, such as low cost, environmental compatibility, nontoxicity, earth‐abundance, etc., which merit it an ideal candidate for modern devices, such as batteries, sensors, and optoelectronics, among others. The controllable and precise synthesis of SnS‐based nanostructures with various crystal phases, sizes, and compositions holds great potential for high‐performance devices as crucial components. To further improve their performance for next‐generation devices, the nanoengineering of SnS‐based nanostructures has been extensively explored over the past decade. In this review, the latest research and progress on SnS‐based nanostructures, including 0D, 1D, 2D, and 3D pure SnS nanostructures, and SnS‐based loaded, sandwiched or encapsulated models are comprehensively presented, focusing on their synthetic approaches, fundamental properties, and fascinating applications such as batteries and solar cells, catalysis (photocatalysis and electrocatalysis), optoelectronics, sensors, ferroelectrics, thermoelectrics, nonlinear properties, and biomedical applications. Finally, the critical challenges and constructive perspectives are discussed for further improvement of the SnS‐based nanostructures in these burgeoning fields.

## Graphical abstract

This review provides an overview of the controlled synthesis and versatile applications of SnS‐based nanostructures, and presents insights on the challenges and future opportunities in the research area. It is envisioned that the controllable manipulation techniques and unique properties of SnS‐based nanostructures can inspire more efforts toward the next‐generation nanodevices based on SnS‐related nanostructures.

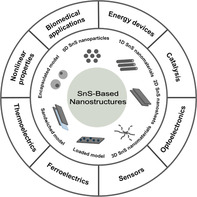

## 1 Introduction

Tin‐based binary chalcogenide Sn–X (X = S, Se, Te) compounds have attracted extensive interest in the optical, optoelectronic, catalysis, and flexible systems due to their intriguing performances.^[^ 1, 2, 3, 4, 5, 6, 7 ^]^ The Sn–X (X = S, Se, Te) compounds are typically layered materials and usually have three crystalline phases: hexagonal, monoclinic, and orthorhombic; orthorhombic phase is denoted by chemical formula SnX, and hexagonal and monoclinic phases are labeled as SnX_2_.^[^ ^8^ ^]^ Until now, SnSe and SnTe compounds have already achieved great progress in many fields, especially for thermoelectronics and ferroelectrics,^[^ 9, 10, 11, 12 ^]^ and many important reviews have been reported on SnSe and SnTe compounds due to their rapid development.^[^ 8, 13, 14, 15, 16 ^]^ For example, in 2020, Chen et al.^[^ ^14^ ^]^ summarized a comprehensive overview of controlled synthesis, characterization, and thermoelectric performance in SnSe. In 2018, Shi et al.^[^ ^8^ ^]^ summarized the growth, characterization, and applications (photovoltaics, supercapacitors, rechargeable batteries, phase‐change memory devices, and topological insulator) of SnSe. In 2018, Li et al.^[^ ^15^ ^]^ reviewed the development of SnS, SnSe, and SnTe, mainly focusing on the controllable tuning of the electron and phonon transport as well as the challenges for further optimization and practical applications. Among Sn–X (X = S, Se, Te) compounds, tin monosulfide (SnS) as a typical black phosphorus analogue, has also received intensive scientific attention due to its unique properties, such as inexpensive, sustainable, suitable bandgap between Si (1.12 eV) and GaAs (1.43 eV),^[^ ^17^ ^]^ high absorption coefficient (10^−4 ^cm^−1^),^[^ ^18^ ^]^ strong mechanical properties, and anisotropic optoelectronic,^[^ 19, 20, 21 ^]^ which are of great value in optoelectronics, catalysis, sensors, and nanomedicines.^[^ 7, 22, 23, 24, 25, 26, 27 ^]^ In addition, layered SnS with suitable spacing structures hold promising potentials in the field of lithium (Li)‐ion batteries and sodium (Na)‐ion batteries due to their relatively high electrical conductivity and theoretical capacity.^[^ 28, 29, 30, 31, 32 ^]^ However, although popular SnS has achieved great progress in a variety of fields, few comprehensive reviews have hitherto focused on the field of SnS‐based nanostructures and their fascinating applications.

**Figure 1:**
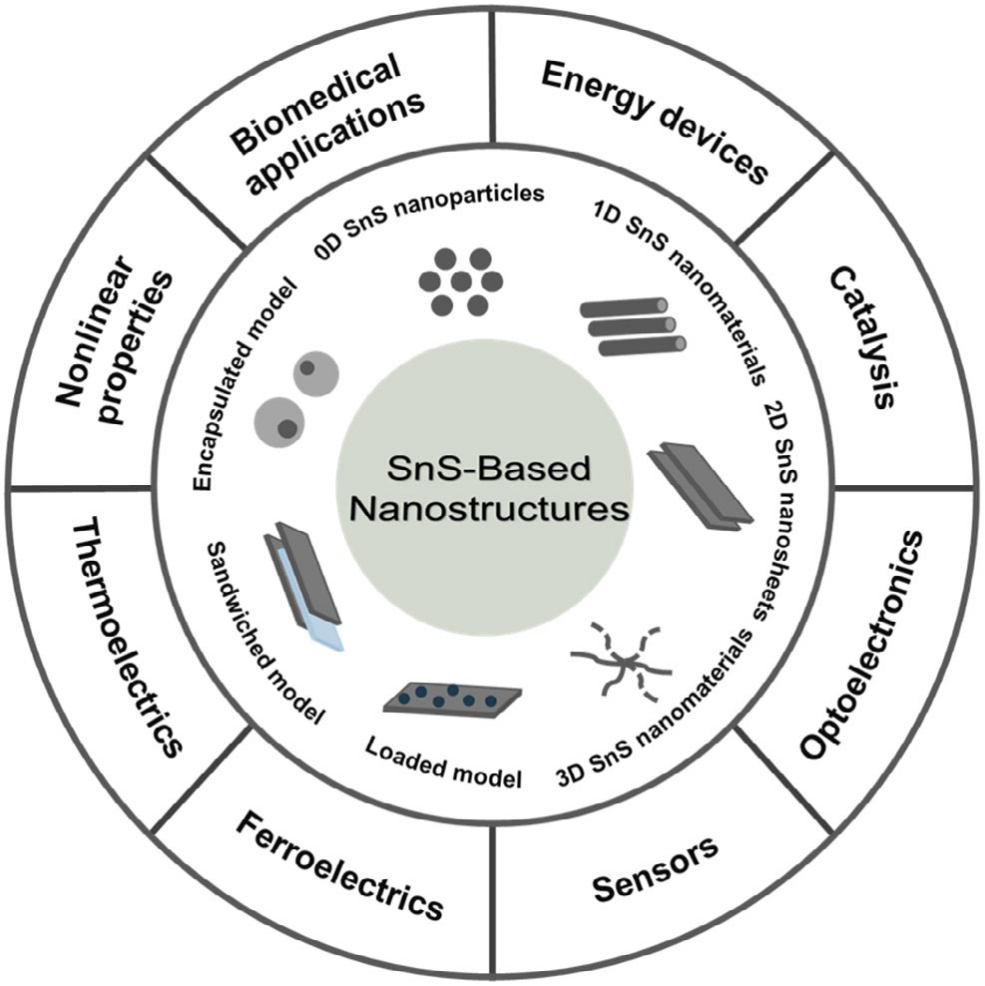
Schematic diagram of the morphology control of SnS‐based nanostructures and their versatile applications for next‐generation devices.

Inspired by important reviews on SnSe reported by Chen^[^ ^14^ ^]^ and Li^[^ ^8^ ^]^ in recent years, we comprehensively summarized the synthesis, characterization, and the latest progress of SnS‐based nanostructures, as shown in **Figure** **1**. First, the most commonly employed techniques (hydrothermal, vapor deposition, electrostatic assembly, etc.) for preparing SnS and SnS‐based nanostructures are presented in detail with their recent progress. Furthermore, the fundamental properties (crystal structure, electronic band structure, optical property, and Raman spectroscopy) and diverse applications (batteries, solar cells, catalysis, optoelectronics, sensors, ferroelectrics, thermoelectrics, nonlinear properties, and biomedical applications) of the SnS‐based nanostructures are also discussed. Finally, the challenges and future opportunities in this community have also been presented at the end of the review.

## 2 Synthesis of SnS‐Based Nanostructures

To understand the structure–property of any nanostructures, it is essential to precisely synthesize and thoroughly characterize the desired nanostructures.^[^ 33, 34, 35, 36, 37, 38, 39, 40, 41 ^]^ With the rapid development of synthetic techniques, a variety of approaches have been employed to synthesize SnS‐based nanostructures, including 0D, 1D, 2D, and 3D pure SnS nanostructures, and SnS‐based loaded, sandwiched, or encapsulated model. In this section, these approaches are briefly summarized in **Table** **1**, and it should be noted that only the commonly used approaches are presented which do not include all the synthetic approaches.

**Table 1:** Strategies used for the synthesis of SnS‐based nanostructures

SnS‐based nanostructuresa)	Sample	Synthetic route	Sn source	S source	Reaction temperature [°C]	Atmosphere or solvents	Reference
0D SnS	SnS quantum dots (QDs)	Solvothermal method	SnCl_2_ or SnBr_2_	Na_2_S	r.t. or 70	Glycerol or ethylene glycol (EG)	[45, 266]
	SnS nanoparticles (NPs)	Solvothermal method	[Sn‐[N(SiMe_3_)_2_]_2_]	Thioacetamide (TAA)	120‐180	N_2_	[46]
	SnS NPs	Solvothermal method	Sn_6_O_4_(OH)_4_	TAA	150	Oleic acid (OA)	[47]
1D SnS	SnS nanowires	Chemical vapor deposition (CVD)	SnS powder	SnS powder	700	Ar and H_2_	[60]
	SnS nanowires	Template‐assisted pulsed electrochemical deposition	SnCl_2_	Na_2_S_2_O_3_	10	Deionized (DI) water	[67]
	SnS nanoribbons	Solvothermal method	SnCl_2_ or SnI_4_	S powder	100 or 180	Oleylamine	[61, 65]
	SnS nanorods	Solvothermal method	SnCl_2_•2H_2_O	Na_2_S	140‐200	DMF or EG	[66, 267, 268]
	SnS nanobelts	Hydrothermal method	SnCl_2_•2H_2_O	TAA	170	DI water	[62]
2D SnS	SnS nanosheets (NSs)	Plasma enhanced CVD	SnCl_2_•2H_2_O or SnCl_4_•5H_2_O	Na_2_S_2_O_3_•5H_2_O	600	Ar	[269]
	SnS NSs	Liquid phased exfoliation (LPE)	SnS powder	SnS powder	r.t.	N‐methyl‐2‐pyrrolidone (NMP), acetone	[2, 36, 176, 270]
	SnS NSs	CVD	SnS powder	S powder	650	Ar and H_2_	[271]
	SnS NSs	Molecular beam epitaxy	SnS powder	SnS powder	120‐320	Vacuum	[83]
	SnS NSs	Hydrothermal method	SnCl_2_•2H_2_O	Na_2_S•9H_2_O or thiourea	190‐200	EG, DI water	[68, 184]
	SnS NSs	Physical vapor deposition (PVD)	SnS powder	SnS powder	410‐470	N_2_	[272]
	SnS NSs	Electrochemical exfoliation	Bulk SnS	Bulk SnS	N/A	Na_2_SO_4_	[5]
3D SnS	SnS nanoflowers	Chemical bath deposition	SnCl_2_•2H_2_O	TAA	80	DI water	[87]
	SnS nanoflowers	Solvothermal method	SnCl_2_•2H_2_O or SnI_2_	Oleylamine sulfide	200	Oleylamine	[32, 99]
	SnS nanoflowers	Hydrothermal method	SnCl_2_•2H_2_O	Thioglycolic acid	108‐160	DI water	[273]
Loaded model	SnS NP/Ti_3_C_2_ NS	Electrostatic assembly	SnS_2_	(NH_4_)_2_S	600	DI water	[113]
	SnS NS/carbonized bacterial cellulose nanofiber	Hot bath method and carbonization process	SnCl_4_	TAA	80	Ethanol	[114]
	SnS NS/GeS NS	PVD	SnS powder	SnS powder	650	Ar and H_2_	[108]
	SnS/MoS_2_	CVD	SnCl_2_	MoS_2_	450	Ar	[127]
	SnS NS/MoO_3_ nanorod	Solvothermal method and etching process	SnCl_2_•2H_2_O	TAA	500	EG	[115]
	SnS NS/porous microtube	Solvothermal and thermal‐treatment method	SnCl_2_•2H_2_O	Thiourea	400	EG	[111]
	Graphene/SnS nanobelt	Carbon‐plasma method	SnCl_2_•2H_2_O	TAA and urea	400	DI water	[63]
Sandwiched model	Zn_0.34_Cd_0.66_S/SnS/Mo	PVD	SnS granule	SnS granule	600	Ar	[133]
	Au/SnS/TiO_2_	Solvothermal method	SnCl_2_	Thiourea	350‐450	Ar	[134]
	N‐doped graphene (NG)/SnS/NG	Solution method	SnCl_4_•5H_2_O	Dodecanethiol	650	N_2_	[137]
	CdS/SnS/Mo	Chemical bath deposited	SnS powder	SnS powder	80	Vacuum	[135]
	SnS NS/polypyrrole (PPy)	In situ polymerization route	ZnSn(OH)_6_ microcubes	TAA	160	DI water	[138]
Encapsulated model	MoS_2_@SnS QD/C nanosheet network (CNN)	Hydrothermal method	SnCl_4_•5H_2_O	TAA	200	DI water	[142]
	SnS NP/ N‐doped porous C nanosheet	Freeze‐drying and calcination	SnCl_4_	Thiourea	600	DI water	[143]
	SnS/C nanofibers	Low‐temperature vulcanization	SnO_2_	Thiourea	700	Ar	[144]
	Co‐, N‐modified porous C fibers@SnS@graphene	CVD	SnS_2_	SnS_2_	500	Ar and H_2_	[69]
	SnS/C nanofibers	Electrospinning	SnCl_2_•2H_2_O	Thiourea	550‐750	Ar and H_2_	[30]

a) r.t.: room temperature; N/A: not applicable.

### 2.1 Synthesis of SnS Nanostructures

#### 2.1.1 Synthesis of 0D SnS Nanomaterials

**Figure 2:**
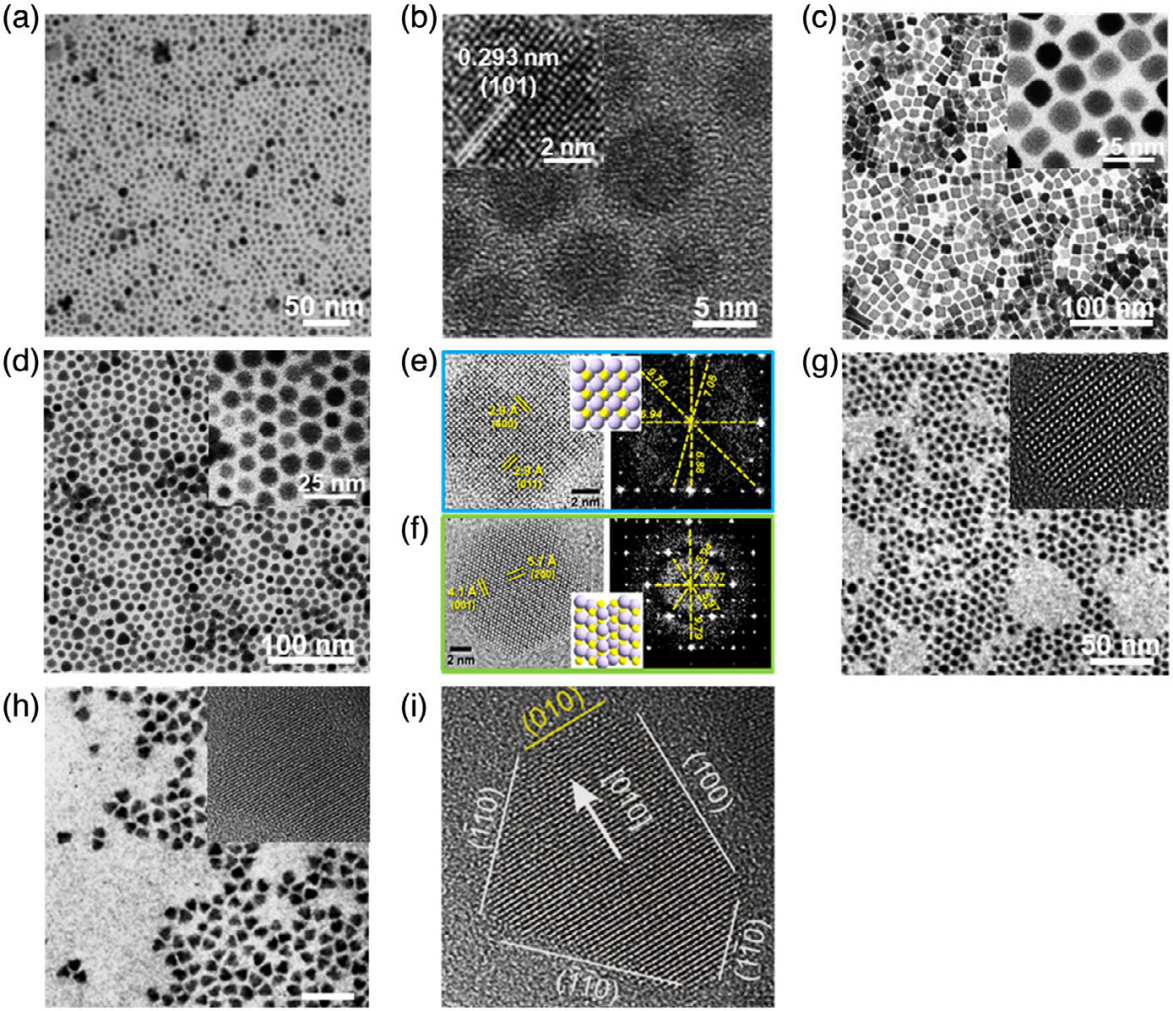
Structural characterizations of the as‐fabricated 0D SnS nanomaterials. a) TEM image, and b) HRTEM image of the as‐synthesized 0D SnS NPs synthesized by a solvothermal method; inset showing the HRTEM image of one single SnS NP. Reproduced with permission.^[^ ^47^ ^]^ Copyright 2010, The Royal Society of Chemistry. c) Representative low‐magnification TEM image of the as‐prepared 0D SnS nanocubes (inset showing the high‐magnification TEM image); d) Representative low‐magnification TEM image of the SnS spherical polyhedra (inset showing the high‐magnification TEM image); e) HRTEM image of a SnS nanocube taken from the (011) zone axis with measured lattice fringes and the resulting FFT, which can be indexed to SnS (011) and f) HRTEM image of a SnS spherical polyhedron taken from the (010) zone axis with measured lattice fringes and the resulting FFT, which can be indexed to SnS (010). Reproduced with permission.^[^ ^50^ ^]^ Copyright 2013, American Chemical Society. HRTEM images of g) spherical‐ and h) triangular‐shaped SnS nanocrystals (insets showing the corresponding enlarged images); i) HRTEM depicting the termination facets of the triangular SnS nanocrystals. Reproduced with permission.^[^ ^46^ ^]^ Copyright 2008, American Chemical Society.

Narrow bandgap SnS semiconductor nanomaterials, composed of low‐cost, earth abundant, and eco‐friendly elements, have a strong optical activity in the near‐infrared (NIR) and infrared (IR) regions which have great potentials in the field of NIR photodetectors and biomedical applications.^[^ 42, 43, 44 ^]^ 0D nanoparticles have a size range from 1 to 100 nm, which have received intensive attention in the past two decades in many fields, such as energy storage and conversion, catalysis, optoelectronics, sensors, and biomedicines, due to their quantum effect.^[^ 33, 34, 36 ^]^ Until now, a number of approaches have been employed to synthesize the 0D SnS nanomaterials with narrow size distribution.^[^ 31, 42, 43, 44, 45, 46, 47 ^,^ 47, 48, 49, 50 ^]^ For example, in 2010, Ning et al.^[^ ^47^ ^]^ reported 0D SnS NPs by a solvothermal method using Sn_6_O_4_(OH)_4_ as a Sn source and TAA as a S source. By strictly controlling the molar ratio of Sn to S, the 0D SnS NPs with uniform distribution can be obtained, as shown in **Figure** **2**a. The diameter of the as‐synthesized 0D SnS NPs is around 5 nm with a standard deviation of only 3%. High‐resolution transmission electron microscopy (HRTEM) image displays the high crystallinity of the as‐synthesized 0D SnS NPs and lattice of NPs can be clearly obtained (Figure 2b). HRTEM image of one single SnS NPs shows the interplanar distance of 0.293 nm (Figure 2c), which can be assigned to the (101) lattice fringe of orthorhombic SnS. Furthermore, in 2013, Biacchi et al.^[^ ^50^ ^]^ reported the high‐yield synthesis of colloidal 0D SnS nanocubes and spherical polyhedra by a solvothermal method. The representative TEM images at different magnifications of the as‐synthesized 0D SnS nanocubes (Figure 2d) and spherical polyhedra (Figure 2e) show good uniformity in both morphology and size. Statistical analysis of the as‐synthesized 0D SnS nanocubes displays an average diameter of 11.5 ± 1.9 nm with an estimated morphological yield of 95% (Figure 2d), while the 0D SnS spherical polyhedra has an average diameter of 9.7 ± 1.5 nm with an estimated morphological yield of 92% (Figure 2e). Figure 2f displays a measured distance of 2.9 Å, which can be indexed to both the (400) and (011) lattice fringes of α‐SnS crystal, and it matches well with the values determined from the fast Fourier transform (FFT) analysis. Figure 2g shows measured distances of 4.1 and 5.7 Å of the as‐synthesized 0D SnS spherical polyhedra, which are corresponding to the (001) and (200) planes of α‐SnS, respectively, and the resulting FFT presents a diffraction pattern that can be assigned to α‐SnS (010). In addition, 0D SnS spherical‐ and triangle‐shaped nanocrystals were successfully synthesized by a solution method.^[^ ^46^ ^]^ The as‐synthesized 0D SnS nanocrystals are uniform with a very low size distribution under no size selective protocols. The shape of 0D SnS nanocrystals can be readily controlled by tuning the OA/oleylamine ratio (OLA), that is, the size of spherical SnS NPs obtained at the predetermined OA/OLA molar ratio of 0.5 (Figure 2h) increases with the OLA concentration, and meanwhile the shape of the NPs becomes angular, while a distinct triangular or even truncated triangular shape with angles of 60° can be clearly observed at a higher OLA concentration (OA/OLA molar ratio of 1, Figure 2i). It is elucidated that if the boundaries of the NPs in Figure 2j are considered as facets, the triangular NPs are predominately terminated by (100) and (110) facets, both of which contain Sn and S atoms when exposed to the vacuum, and only the (010) facets consist of a single atom species (Sn or S), marked yellow in Figure 2j.

#### 2.1.2 Synthesis of 1D SnS Nanomaterials

**Figure 3:**
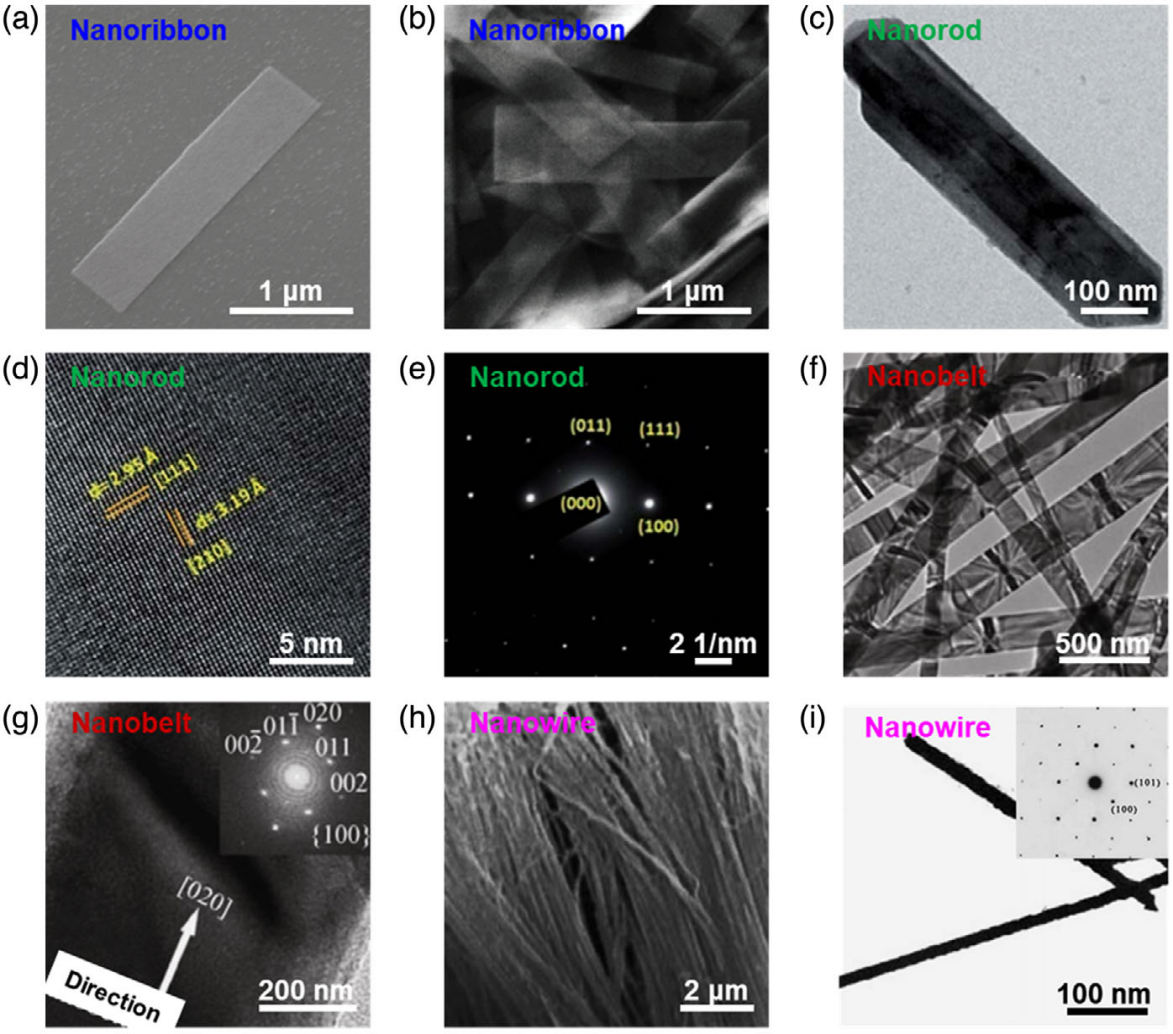
Structural characterizations of the as‐fabricated 1D SnS nanomaterials. SEM images of a) a solution‐synthesized nanoribbon on a SiO_2_/Si substrate and b) a high concentration of nanoribbons. Reproduced with permission.^[^ ^65^ ^]^ Copyright 2018, The Royal Society of Chemistry. c) TEM image, d) HRTEM image, and e) SAED pattern of the SnS nanorods synthesized by a solvothermal method at 160 °C. Reproduced with permission.^[^ ^66^ ^]^ Copyright 2014, The Royal Society of Chemistry. f) TEM image of the SnS nanobelts synthesized by a solvothermal method at 170 °C, and g) HRTEM image (inset showing its SAED pattern). Reproduced with permission.^[^ ^62^ ^]^ Copyright 2013, Springer. h) A typical cross‐section SEM image of the SnS nanowires synthesized by the template‐assisted pulsed electrochemical deposition in the porous AAO template, and i) TEM image of the SnS nanowires with a diameter of ≈50 nm (inset showing the SAED pattern). Reproduced under the terms of the CC‐BY 2.0 license.^[^ ^67^ ^]^ Copyright 2009, The Authors, published by Springer.

1D nanostructures that are nanosized with less than 100 nm in two dimensionalities while being elongated in the third dimensionality, such as nanowires, nanotubes, nanoribbons, nanorods, and nanobelts, are of great interest owing to their unique properties.^[^ 33, 34, 35, 51, 52, 53, 54, 55, 56, 57, 58, 59 ^]^ As we know, 1D nanostructures provide a very good platform to study the relationship between nanostructures and properties, such as the dependence of electrical and thermal transport or mechanical properties on quantum confinement. They also play an important part in fabricating nanodevices (e.g., nanoelectronics, nanooptoelectronics, nanoelectrochemical, and nanomechanical devices) as functional building blocks. In recent years, 1D SnS nanomaterials with semiconducting property have been widely employed as active materials for a variety of applications, such as photodetectors^[^ 60, 61 ^]^ and batteries.^[^ 62, 63, 64 ^]^ For example, in 2018, Hajzus et al.^[^ ^65^ ^]^ successfully synthesized colloidal 1D SnS nanoribbons by a solvothermal method using SnCl_2_ as the Sn source and S powder as the S source. The as‐synthesized 1D SnS nanoribbons are several microns in length and 20 nm or less in thickness (**Figure** **3**a,b), and the individual SnS nanoribbon (Figure 3a) was chosen to pattern contacts by an electron‐beam lithography to study electrical behavior. Tripathi and Mitra^[^ ^66^ ^]^ successfully synthesized 1D SnS nanorods by a solvothermal method using SnCl_2_•2H_2_O and Na_2_S as precursors and *N,N*‐dimethyl formamide (DMF) as a solvent. Transmission electron microscopy (TEM) image shows that well‐distributed single crystalline nanorods of the as‐synthesized 1D SnS nanorods are formed with a high aspect ratio (Figure 3c). The interatomic *d*‐spacings of the lattice fringes are measured to be 2.95 and 3.19 Å, in good agreement with (110) and (210) planes of α‐SnS crystal (Figure 3d). The single crystalline nature of SnS nanorods with dots indexed to (011), (100) and (111) planes was also confirmed by the selected area electron diffraction (SAED) pattern (Figure 3e). In addition, Lu et al.^[^ ^62^ ^]^ reported that (020)‐oriented 1D SnS nanobelts were successfully synthesized by a hydrothermal method in the absence of any surfactants. The TEM image (Figure 3f) shows that the as‐synthesized 1D nanobelts with width of 80–300 nm mainly lie on the carbon membrane. Figure 3g shows the HRTEM image of the lateral face of a SnS nanobelt, and SAED pattern shows the clear diffraction spots (Figure 3g inset), indicating that the nanobelts are single crystalline. In addition, template‐assisted pulsed electrochemical deposition was carried out to synthesize 1D SnS nanowire arrays in the porous anodized aluminum oxide (AAO).^[^ ^67^ ^]^ The results show that the as‐synthesized 1D SnS nanowires with the nature of single crystalline and present a highly preferential orientation. Figure 3h displays a cross‐section SEM in which a majority of alumina matrix of the AAO template has been removed. It can be observed that the as‐obtained 1D SnS nanowires are well aligned, and uniformly distributed, and have several tens of microns in length, consistent with the thickness of the AAO template employed. Figure 3i shows the TEM image of the 1D SnS nanowires synthesized within the AAO template. The average diameter of the well‐distributed 1D SnS nanowires is approximately 50 nm, which is in good accordance to that of the nanochannels of the AAO template. SAED pattern (Figure 3i inset) taken from a single 1D SnS nanowire illustrates that the 1D SnS nanowires synthesized by this template‐assisted pulsed electrochemical deposition technique are also single crystalline.

#### 2.1.3 Synthesis of 2D SnS Nanomaterials

**Figure 4:**
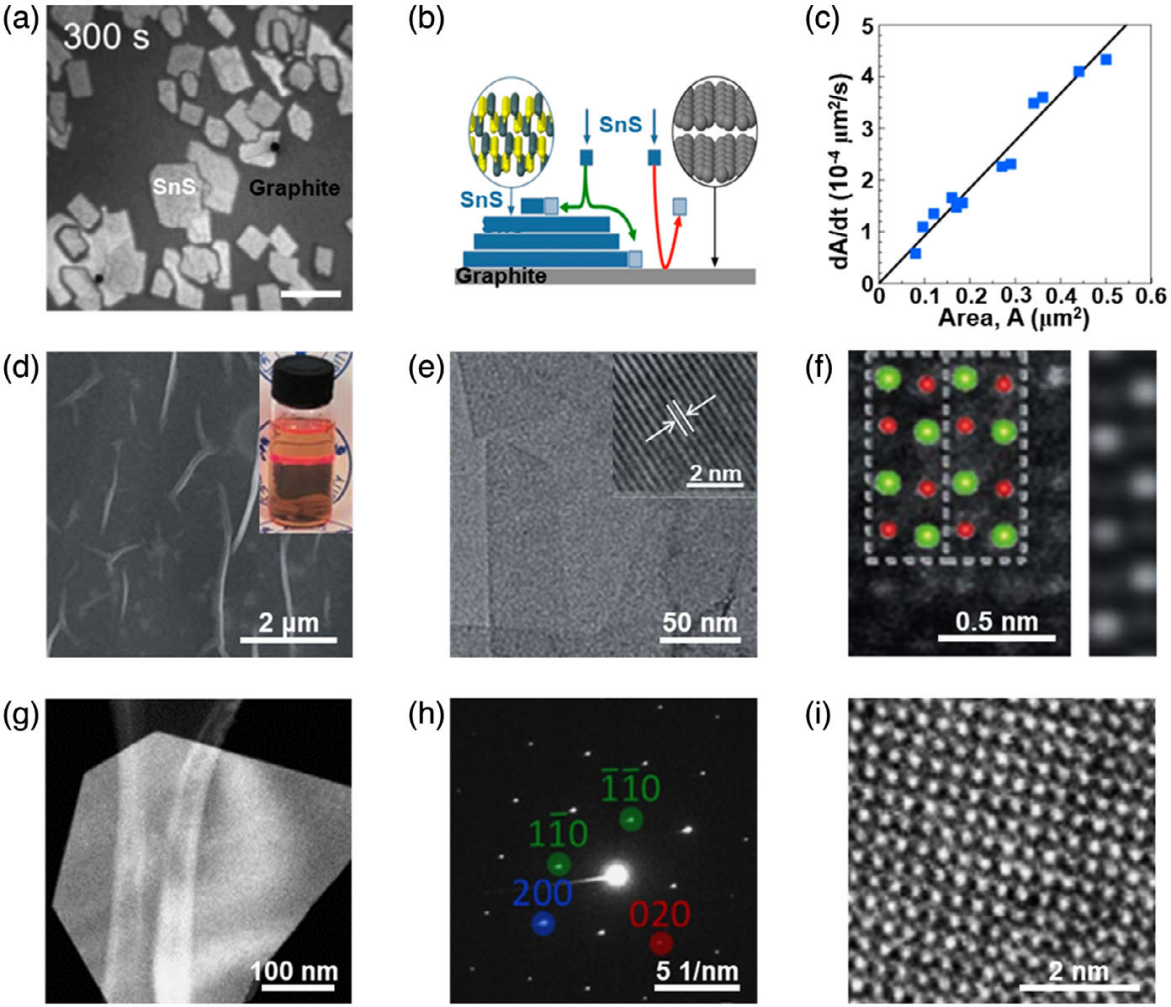
Structural characterizations of the as‐fabricated 2D SnS nanomaterials. a) Time‐lapse image of low‐energy electron microscopy (LEEM) image of SnS molecular beam epitaxy on graphite at 300 s; b) schematic showing the observed predominant adsorption of SnS on existing SnS nuclei and negligible sticking to the graphite substrate; and c) analysis of the SnS lateral growth rate (dA/dt) as a function of the projected flake area, A. Reproduced with permission.^[^ ^83^ ^]^ Copyright 2018, American Chemical Society. d) SEM image of the SnS NSs synthesized via an electrochemical exfoliation route and the corresponding Tyndall effect (inset); e) TEM image of the as‐synthesized SnS NSs and its HRTEM image (inset); and f) HAADF‐STEM image of SnS along the (100) direction (inset showing the crystal structure of SnS in the same projection, where Sn and S atoms are depicted in green and red, respectively) and a simulated HAADF image along (100) using the thickness of 2 unit cells, consistent with the HAADF‐STEM result. Reproduced with permission.^[^ ^5^ ^]^ Copyright 2020, The Royal Society of Chemistry. g) HAADF‐STEM image, h) SAED pattern, and i) HRTEM image of a characteristic SnS NS using Au NPs as the templates. Reproduced with permission.^[^ ^84^ ^]^ Copyright 2019, American Chemical Society.

Over the past decade, 2D nanomaterials (e.g., graphene^[^ 68, 69 ^]^) with a large lateral size and atomically small thickness have drawn extensive attention due to their intriguing physicochemical properties and promising applications, such as energy, detection, catalysis, and optoelectronics.^[^ 36, 41, 51, 70, 71, 72, 73, 74, 75, 76, 77, 78, 79, 80, 81, 82 ^]^ Compared with the rapid progress in 0D and 1D SnS nanostructures, there are much less reports on SnS nanostructures, their synthetic strategies and fascinating properties. In 2018, Sutter et al.^[^ ^83^ ^]^ employed in situ microscopy during molecular beam epitaxy to study the fundamental growth mechanisms of SnS. **Figure** **4**a gives the analysis of real‐time microscopy image at 300 s of the SnS growth by a molecular beam epitaxy technique on a clean, atomically flat graphite van der Waals substrate at a sample temperature of 260 °C. It can be observed that all of the SnS nuclei are parallel to the surface of the graphite van der Waals substrate, which is schematically diagramed in Figure 4b. The rate of lateral expansion (dA/dt) of SnS on graphite at a predetermined time as a function of the projected area (*A*) of the SnS nuclei is plotted (Figure 4c). It can be observed that the dA/dt shows linear relationship with the *A*, and the fitted line almost intersects the coordinate axes at the origin. It is concluded from the linear dependence that the SnS NSs mainly grow by means of SnS adsorption from the vapor phase. In the limit *A* (*A*→0), the disappearance of dA/dt values suggests that the SnS adsorption on the graphite surface has no significant contribution to the SnS growth. In addition, in the same year Chen et al.^[^ ^5^ ^]^ reported a facile electrochemical exfoliation technique to fabricate 2D ultrathin SnS NSs on a large scale using bulk SnS. A two‐electrode system was set up under alternating voltage with the bulk SnS as the working electrode and Pt mesh as the counter electrode in 0.5 m Na_2_SO_4_. There is an obvious layer structure with several wrinkles on the surface of the as‐fabricated 2D SnS NSs in Figure 4d, and a clear Tyndall effect for the 2D SnS NSs dispersion can be observed (Figure 4d inset), further confirming that 2D SnS NSs have been successfully fabricated by the electrochemical exfoliation technique. The TEM image also verifies the layer structure of the as‐fabricated 2D SnS NSs with several hundreds of nanometers in lateral size (Figure 4e), and the HRTEM image exhibits a lattice fringe distance of 0.29 nm, which can be indexed to (101) plane of SnS crystal (Figure 4e inset). A representative high‐angle annular dark‐field scanning transmission electron microscopy (HAADF‐STEM) image for the as‐fabricated 2D SnS NSs along the (100) direction can be observed (left picture in Figure 4f). The arrangement of atomic columns in these images is in good agreement with the structure of SnS (space group *Pnma*). The HAADF image simulation was further performed using the thickness of 2 unit cells, and the result demonstrated the precise crystalline structure of ultrathin 2D SnS NSs (right picture in Figure 4f). Moreover, in 2019, Sutter et al.^[^ ^84^ ^]^ used Au NPs to seed the nucleation, determine the position, and tune the thickness of the 2D SnS NSs. In situ TEM was employed to study the thermal stability and decomposition pathways of the individual 2D ultrathin SnS NSs grown on 5 nm Au NPs during annealing. The TEM image of a characteristic flake can be seen in Figure 4g. The SAED pattern (Figure 4h) and the HRTEM image (Figure 4i) indicate that the main facets define the shape of the 2D SnS NSs as the low‐index (100), (010), and (110) facets, which are perpendicular to the *a* and *b* axes and the basal plane diagonals, respectively. The investigation of 2D SnS NSs nucleating on an individual Au‐rich NP growth seed provides a useful way to obtain sparsely nucleated NSs whose placement on the substrate can be readily identified by isolated Au NPs. The mechanism of the thickness‐controlled growth of the 2D SnS NSs was also proposed: i) small 2D SnS NSs sparsely nucleated on the Au NPs formed on the surface of Si substrate; ii) nucleation and growth of more 2D SnS NSs until the entire Si surface was totally covered by a large quantity of square NSs. In addition, our group used a facile LPE technique to fabricate 2D SnS NSs and the size selection of the as‐fabricated 2D SnS NSs was also carried out by a liquid cascade centrifugation (LCC) technique.^[^ ^36^ ^]^ It was elucidated that the average thickness and lateral size of the as‐fabricated 2D SnS NSs gradually decreased with the increase of centrifugation speed, characterized by TEM, AFM, and dynamic light scattering, and the as‐fabricated 2D SnS NSs possessed excellent stability under acidic, neutral, or basic condition, which holds great promise in photodetection, sensing, modulation, etc. Notably, monolayer SnS^[^ ^85^ ^]^ and few‐layer SnS^[^ ^86^ ^]^ were also successfully fabricated for the systematical study of thickness‐dependent in‐plane ferroelectricity.

#### 2.1.4 Synthesis of 3D SnS Nanomaterials

**Figure 5:**
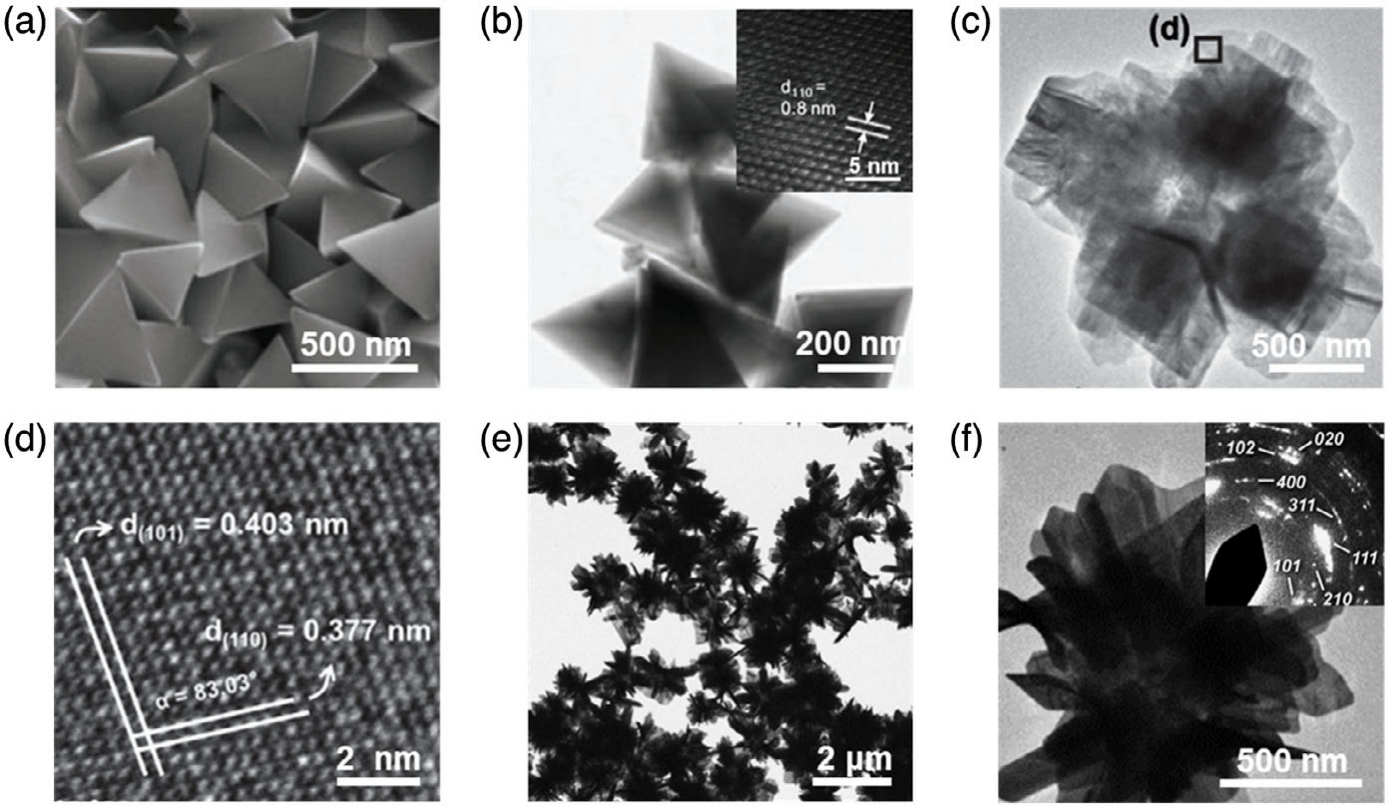
Structural characterizations of the as‐fabricated 3D SnS nanomaterials. a) SEM image of tetrahedral ZB 3D SnS nanomaterials synthesized by a solvothermal method, and b) TEM image; inset showing the corresponding HRTEM image. Reproduced with permission.^[^ ^95^ ^]^ Copyright 2019, Wiley‐VCH. c) TEM image of an individual 3D SnS nanoflower synthesized via a simple polyol method, and d) HRTEM image obtained from the region marked in (c). Reproduced with permission.^[^ ^32^ ^]^ Copyright 2016, Wiley‐VCH. e) TEM image of the 3D SnS nanoflowers synthesized by a solution method, and f) SEM image of a single 3D SnS nanoflower (inset showing its corresponding SAED pattern). Reproduced with permission.^[^ ^99^ ^]^ Copyright 2012, The Royal Society of Chemistry.

Although the controlled synthesis of uniform low‐dimensional (0D, 1D, or 2D) nanostructures has been rapidly exploited in recent years, yet 3D hierarchical nanostructures are also of important significance owing to their remarkable properties and relatively facile synthesis, which have great potential in a variety of fields, such as batteries, supercapacitors, catalysis, and sensors.^[^ 32, 87, 88, 89, 90, 91, 92, 93, 94, 95, 96, 97, 98 ^]^ In 2019, a solvothermal method was carried out by Kim et al.^[^ ^95^ ^]^ to synthesize the zinc blende (ZB) 3D SnS nanomaterials. By simply controlling the Sn and S precursor ratio, reaction time, and temperature, the well‐defined morphology and crystal structure was achieved. The morphological features of the 3D SnS nanostructures characterized by SEM show that they display a tetrahedral morphology with an average diameter of ≈500 nm on a side (**Figure** **5**a,b). The lattice fringe of 0.8 nm (Figure 5b inset) is corresponding to the (110) plane of orthorhombic SnS. It was demonstrated that the ZB 3D SnS nanomaterial‐based solid electrolyte can efficiently suppress the degradation of active materials and electrolyte reduction, and enables a reversible conversion reaction, leading to stable cycling performance.^[^ ^95^ ^]^ Furthermore, a simple polyol process was also performed to fabricate 3D SnS nanoflowers containing hierarchical NS subunits.^[^ ^32^ ^]^ The TEM image shows that the as‐fabricated individual 3D SnS nanoflower is comprised of NSs which are linked to a central core (Figure 5c). The HRTEM image taken from the marked region in Figure 5d exhibits that the 3D SnS nanoflowers display clear lattice *d*‐spacings of 0.377 and 0.403 nm, which are corresponding to the (110) and (101) planes of the orthorhombic SnS. It was reported that the as‐fabricated 3D SnS nanoflowers had a larger surface area and more active electrochemical interfacial areas that were beneficial to Na^+^ insertion and extraction compared with bulk SnS, which could be a competitive candidate as a promising anode material for high‐performance Na‐ion battery. In addition, Vaughn et al.^[^ ^99^ ^]^ successfully synthesized 3D nanoflowers by a solvothermal method using SnI_2_ as the Sn source and oleylamine sulfide as the S source. The TEM image displays that the as‐fabricated 3D SnS nanoflowers have discrete hierarchical nanostructures (Figure 5e), and are comprised of individual NS which are also connected to a central core (Figure 5f), similar to Cho's work.^[^ ^32^ ^]^ SAED pattern (Figure 5f inset) for an ensemble of 3D SnS nanoflowers exhibits consistency with polycrystalline black‐phosphorus‐analogue GeS‐type SnS with no crystalline impurities.^[^ ^99^ ^]^ The as‐fabricated 3D SnS nanoflowers were also found to be promising candidates in energy device, such as Li‐ion batteries, due to their excellent charge/discharge capacity and superior reversibility.

### 2.2 Synthesis of SnS‐Based Nanostructures

**Figure 6:**
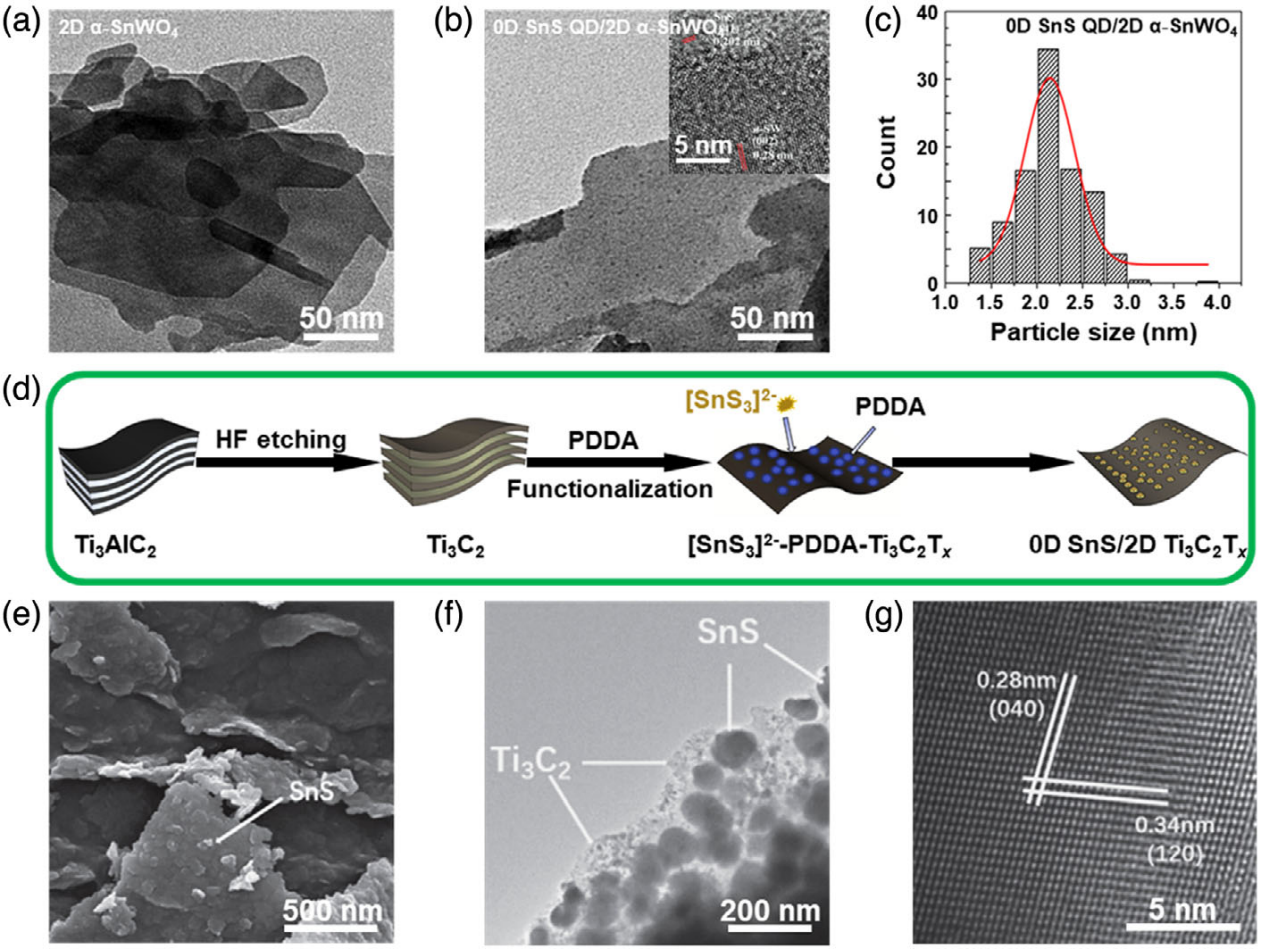
Structural characterizations of the SnS‐based nanostructures with 0D SnS loaded. a) TEM image of the precursor α‐SnWO_4_ NSs; b) TEM image of the as‐obtained 0D SnS QD/2D α‐SnWO_4_ heterostructures synthesized by a one‐pot hydrothermal method (inset showing its HRTEM image), and c) size distribution of 0D SnS QDs measured in (b). Reproduced with permission.^[^ ^118^ ^]^ Copyright 2020, Elsevier B.V. d) Schematic illustration for the synthetic process of 0D SnS QD/2D MXene Ti_3_C_2_T_*x*_ NS heterostructure; e) SEM image, f) TEM image, and g) HRTEM image of the as‐synthesized 0D SnS QD/2D MXene Ti_3_C_2_T_*x*_ NS heterostructures. Reproduced with permission.^[^ ^113^ ^]^ Copyright 2019, Elsevier B.V.

**Figure 7:**
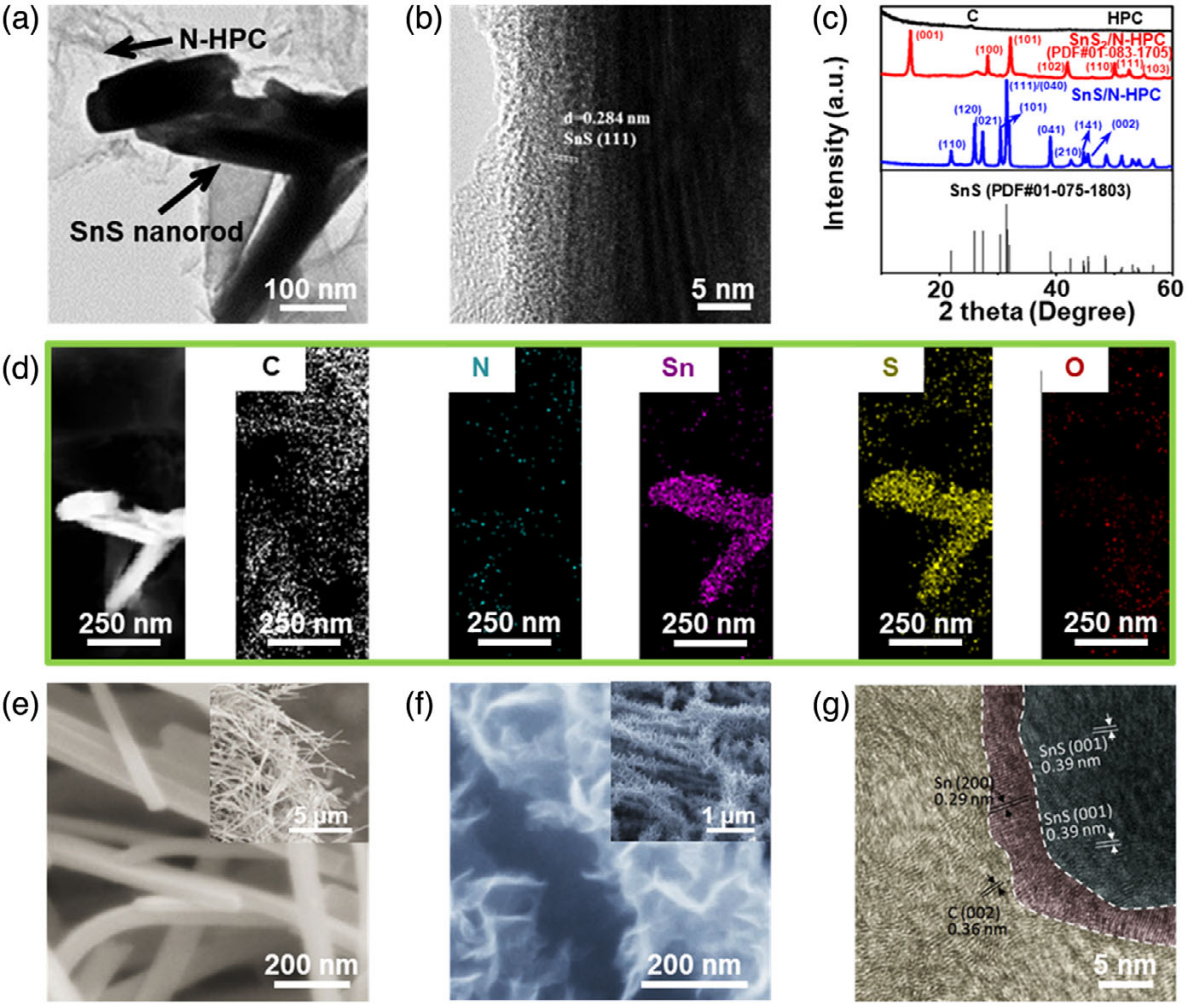
Structural characterizations of the SnS‐based nanostructures with 1D SnS loaded. a) TEM image and b) HRTEM image of N‐HPC/1D SnS nanorod heterostructures synthesized by post‐thermal annealing of SnS_2_ NPs supported on N‐HPC through a hydrothermal process; c) XRD patterns of HPC, SnS, N‐HPC/0D SnS_2_ NP heterostructures, and N‐HPC/1D SnS nanorod heterostructures; and d) HAADF‐STEM image capturing the 1D SnS nanorods on N‐HPC and EDX mapping images of the elemental distribution of C, N, Sn, S, and O. Reproduced with permission.^[^ ^64^ ^]^ Copyright 2020, American Chemical Society. e) SEM image of pure 1D SnS nanobelts synthesized by a one‐step hydrothermal method (inset showing its low‐magnification SEM image); f) SEM image of the hierarchical graphene/1D SnS nanobelt heterostructures bundles fabricated by a rapid carbon‐plasma method (inset showing its low‐magnification SEM image), and g) HRTEM image of the as‐fabricated heterostructures displaying a typical interface between hierarchical graphene and 1D SnS nanobelts. Reproduced with permission.^[^ ^63^ ^]^ Copyright 2018, Wiley‐VCH.

**Figure 8:**
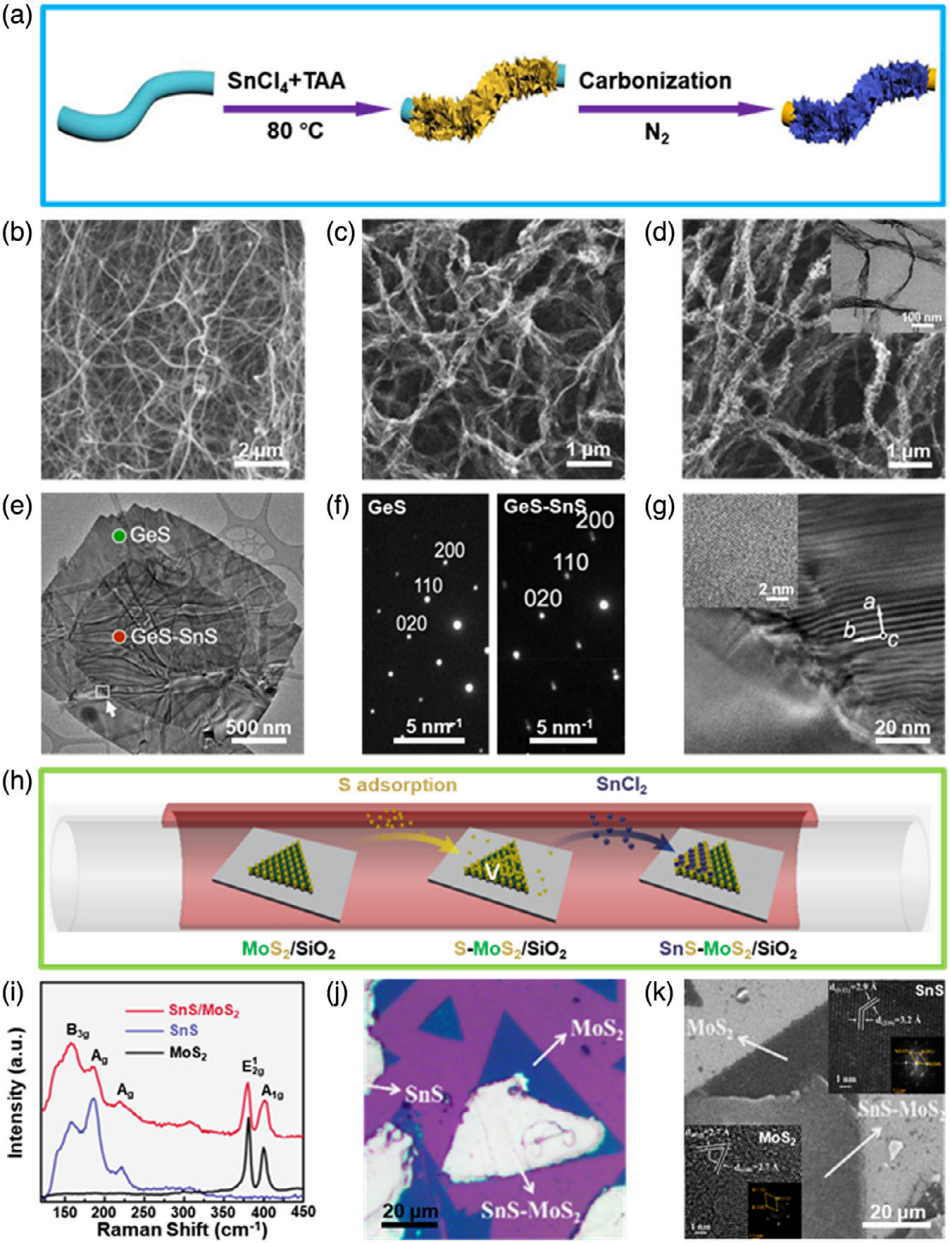
Structural characterizations of the SnS‐based nanostructures with 2D SnS loaded. a) Schematic illustration of the synthesis of 2D SnS NS/CBC nanofiber heterostructures; b) SEM image of BC aerogel; c) SEM image of 2D SnS_2_ NS/BC aerogel heterostructures; and d) SEM image of 2D SnS_2_ NS/CBC aerogel heterostructures (inset showing the corresponding TEM image). Reproduced with permission.^[^ ^114^ ^]^ Copyright 2021, Elsevier B.V. e) STEM and f) HAADF‐STEM images of a characteristic 2D SnS/2D GeS heterostructure, and g) HRTEM image near the interface of the in‐plane heterostructure (arrow in (e)); inset showing HRTEM of the GeS region (lower left in (e)). Reproduced with permission.^[^ ^108^ ^]^ Copyright 2020, American Chemical Society. h) Schematic illustration of the growth process of 2D SnS/2D MoS_2_ heterostructures; i) Raman spectra of MoS_2_, SnS, and the as‐fabricated 2D SnS NS/2D MoS_2_ NS heterostructures; j) optical image and k) SEM image of the as‐fabricated SnS/2D MoS_2_ heterostructures (insets showing the corresponding FFT patterns). Reproduced with permission.^[^ ^127^ ^]^ Copyright 2020, American Chemical Society.

**Figure 9:**
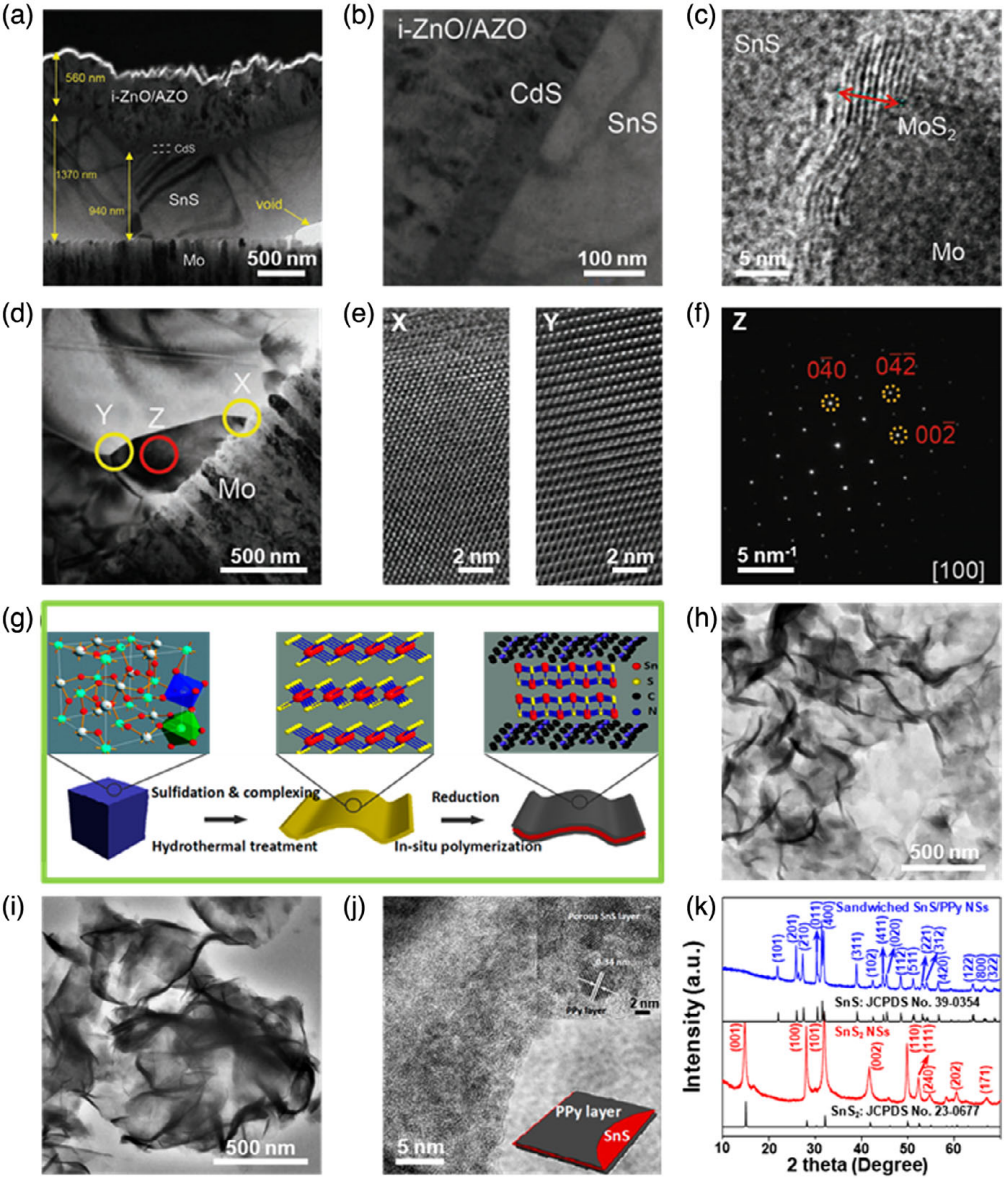
Structural characterizations of the SnS‐based nanostructures with 2D SnS sandwiched. a) Low‐magnification TEM image of the Mo/SnS/CdS/i‐ZnO/AZO heterostructures, and TEM images of the b) SnS/CdS and c) Mo/SnS interfaces; the HRTEM images in (e) correspond to the regions X and Y shown in d,f) SAED pattern of the region Z in (d) with the (100) zone axis. Reproduced with permission.^[^ ^135^ ^]^ Copyright 2018, Wiley‐VCH. g) Schematic illustration of the fabrication of sandwich‐like 2D SnS NS/PPy heterostructures from uniform ZnSn(OH)_6_ microcubes; h) low magnification TEM image of NS‐like SnS_2_ precursor revealing that these 2D NSs have a smooth surface and relatively thin thickness; i) low‐ and j) high‐magnification TEM images of 2D SnS NS/PPy heterostructures; the bottom‐right inset of (j) showing the schematic structure sandwich‐like NSs and the upper‐right inset of (j) showing that the pristine SnS_2_ smooth NSs were in situ reduced into porous SnS and fully encapsulated by conductive PPy layers; and k) XRD patterns of the SnS_2_ ultrathin NSs and 2D SnS NS/PPy heterostructures, clearly showing that the SnS_2_ precursor has been converted into SnS completely. Reproduced with permission.^[^ ^138^ ^]^ Copyright 2016, American Chemical Society.

**Figure 10:**
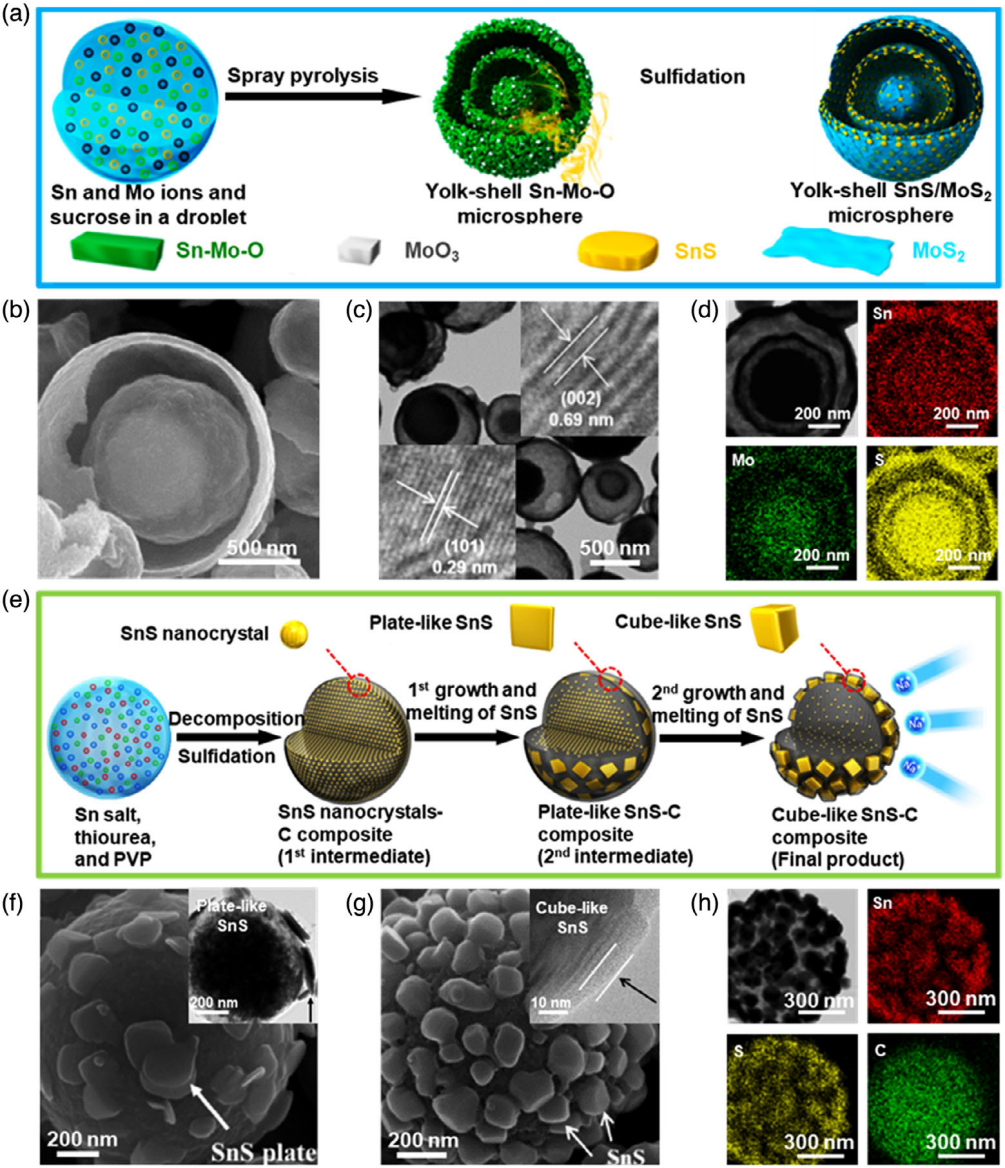
Structural characterizations of the SnS‐based nanostructures with 0D SnS encapsulated. a) Schematic illustration of the fabrication process of the yolk–shell SnS–MoS_2_ composite microsphere by a spray pyrolysis and subsequent sulfidation process; b) SEM image, and c) TEM image of the yolk–shell SnS–MoS_2_ composite microspheres; inset of (c) showing the corresponding HRTEM image; and d) EDX mapping images of Sn, Mo, and S components. Reproduced with permission.^[^ ^147^ ^]^ Copyright 2015, American Chemical Society. e) Schematic diagram of the detailed formation mechanism of the SnS/C microsphere in the one‐pot spray pyrolysis; high‐magnification SEM image of the as‐fabricated SnS/C microspheres prepared at f) 700 °C and g) 900 °C; inset of (f) shows TEM image and inset of (g) shows the HRTEM image; and h) EDX mapping images of Sn, S, and C components. Reproduced with permission.^[^ ^154^ ^]^ Copyright 2015, Springer.

**Figure 11:**
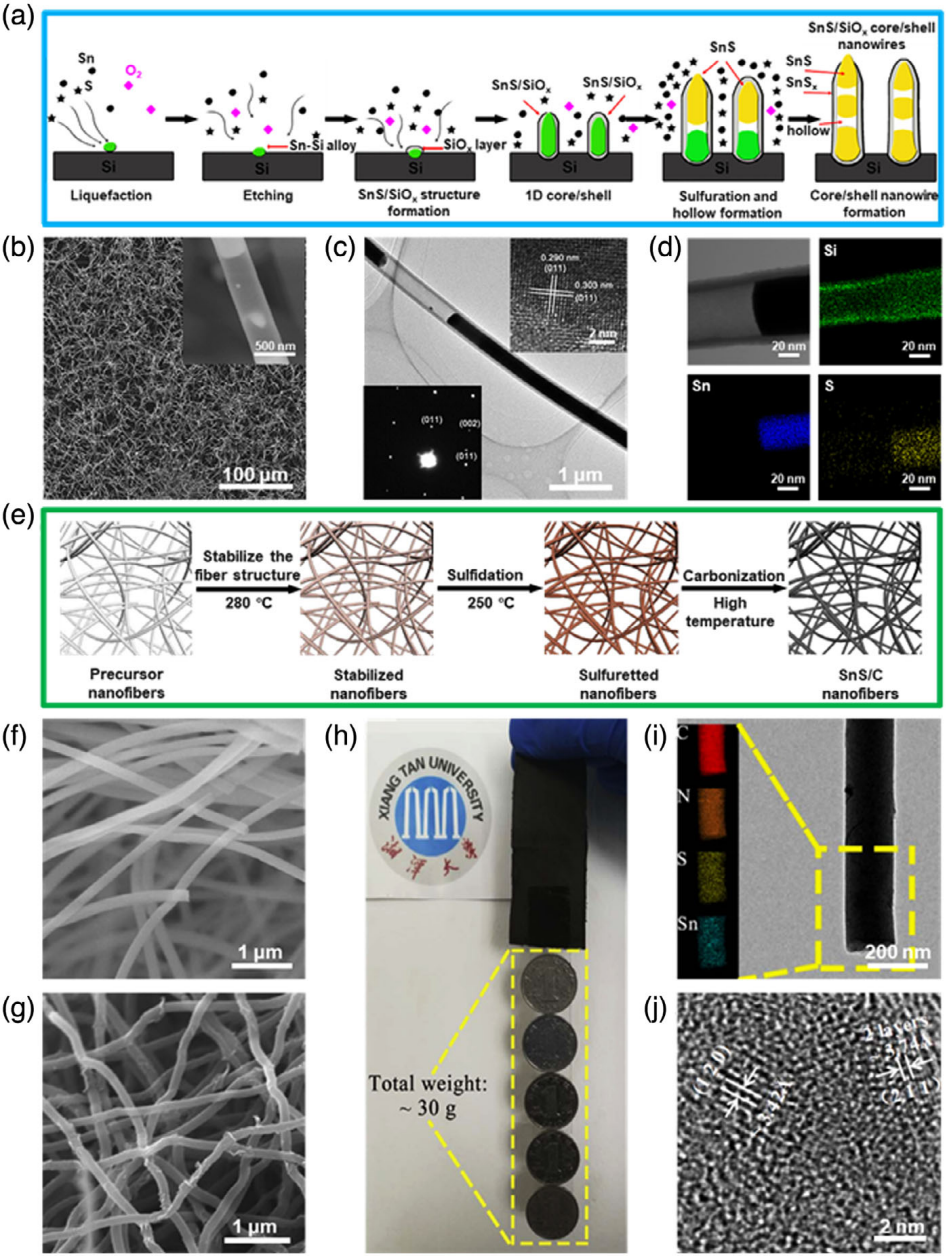
Structural characterizations of the SnS‐based nanostructures with 1D SnS encapsulated. a) Schematic illustration of the growth process of SnS/SiO_*x*_ core/shell nanowires; b) low‐magnification SEM image of the as‐fabricated SnS/SiO_*x*_ core/shell nanowires; inset showing the high‐magnification SEM image; c) TEM morphology image of a single SnS/SiO_*x*_ core/shell nanowire; upper‐right inset of (c) showing its HRTEM image and bottom‐left inset of (c) showing its SAED pattern; d) TEM image of a SnS/SiO_*x*_ core/shell nanowire with hollow structure and its corresponding EDX elemental mapping images. Reproduced with permission.^[^ ^155^ ^]^ Copyright 2020, Elsevier B.V. e) Schematic illustration of the synthesis process for the SnS/C nanofibers; f) SEM images of the precursor SnCl_2_/PVP nanofibers and g) the as‐prepared SnS/C nanofibers fabricated at 650 °C; h) the picture of several Chinese coins (the weight of each 1 yuan coin is about 6 g) taped to the edge of SnS/C nanofibers; i) TEM images of SnS/C nanofibers; inset showing its EDX elemental mapping of the area, marked by the yellow rectangle; and j) its corresponding HRTEM image. Reproduced with permission.^[^ ^30^ ^]^ Copyright 2019, Elsevier B.V.

**Figure 12:**
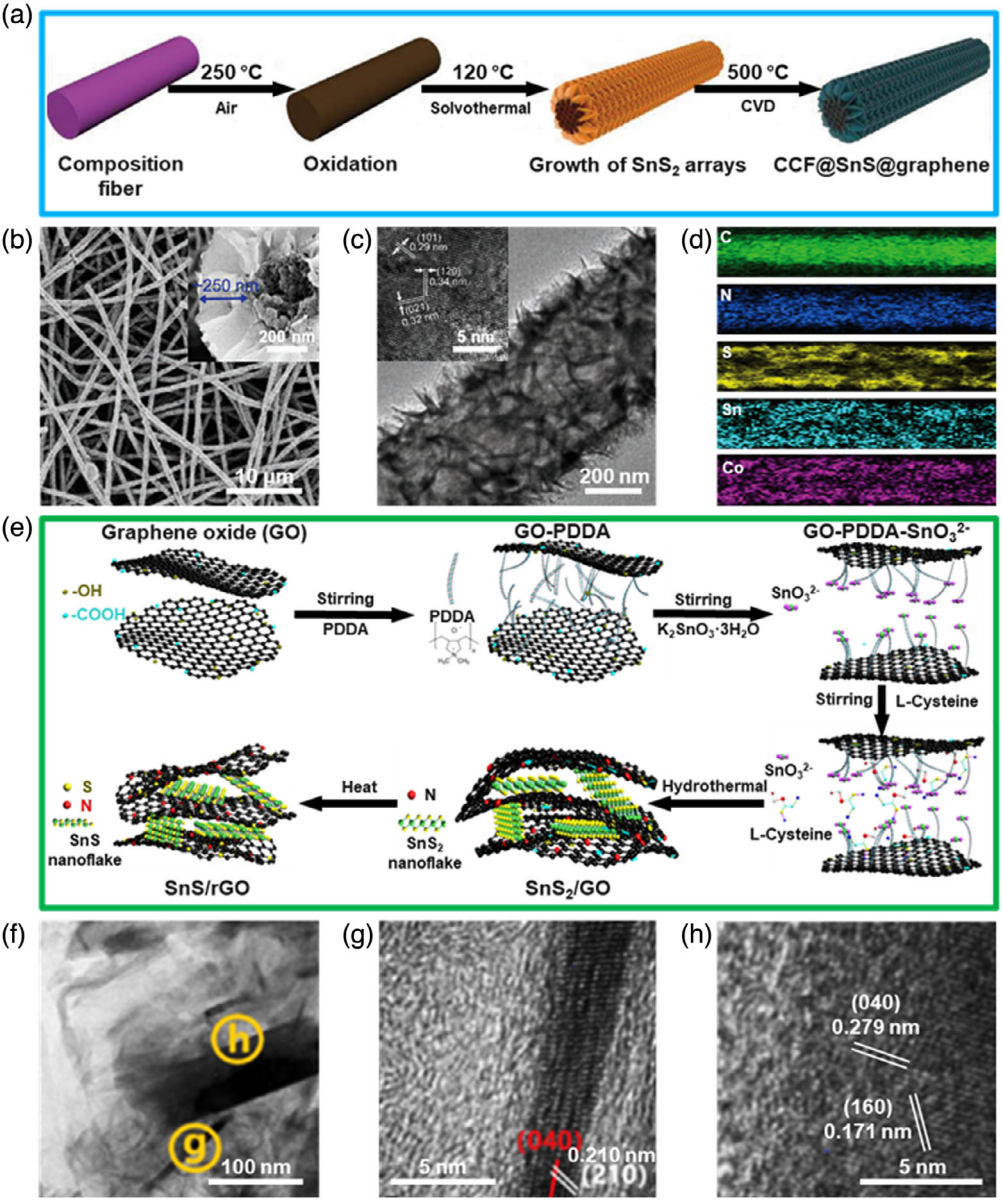
Structural characterizations of the SnS‐based nanostructures with 2D SnS encapsulated. a) Schematic illustration of fabrication procedure of CCF@SnS@graphene; b) low‐magnification SEM image of CCF@SnS@graphene and its corresponding high‐magnification SEM image (inset); c) TEM image of the as‐prepared CCF@SnS@graphene and its HRTEM image (inset); d) EDX mapping of C, N, Sn, S, and Co components. Reproduced with permission.^[^ ^69^ ^]^ Copyright 2020, Wiley‐VCH. e) Schematic diagram of the fabrication process of the 2D SnS NS/2D rGO NS heterostructures; f) TEM image of the as‐fabricated 2D SnS NS/2D rGO NS heterostructures and g,h) the corresponding HRTEM images correspond to the regions shown in (f). Reproduced with permission.^[^ ^159^ ^]^ Copyright 2019, American Chemical Society.

Despite pure SnS nanostructures has shown superior advantages in many applications, several drawbacks (e.g., relatively low flexibility and easy restacking) make them difficult to satisfy the growing demand of many civil and military applications.^[^ 29, 100, 101, 102, 103, 104, 105 ^]^ To this end, functional SnS‐based nanostructures have received considerable research interest,^[^ 106, 107 ^]^ and have been successfully synthesized by hybridizing with other nanomaterials, such as 2D SnS/2D GeS heterostructures,^[^ ^108^ ^]^ wrap‐around SnS–SnS_2_ core‐shell heterostructures,^[^ ^109^ ^]^ ultrathin self‐organized trigonal SnS–SnS_2_ twist‐heterostructures,^[^ ^110^ ^]^ ultrathin SnS/SnS_2_ sandwiched heterostructures,^[^ ^86^ ^]^ and 2D SnS/1D carbon nanotube (CNT) heterostructures,^[^ 111, 112 ^]^ to exploit the full potential and go beyond the limits. To simplify the classification, the currently reported SnS‐based nanostructures are divided into three models, that is, loaded model, sandwiched model, and encapsulated model (Figure 1). In the loaded model, 0D, 1D, and 2D SnS nanostructures as active materials are tightly loaded on the substrates (**Figures** 6, 7, 8); in the sandwiched model, the active 2D SnS NSs are well sandwiched between coating layers (inorganic nanomaterials or polymers) and the obtained composites still maintain 2D structure (**Figure** **9**); in the encapsulated model, 0D, 1D, and 2D SnS nanostructures are entirely wrapped by coating materials (**Figures** 10, 11, 12). In the next part, three models of SnS‐based nanostructures will be comprehensively discussed.

#### 2.2.1 Loaded Model

In the loaded model, the electrons can quickly transfer across the substrate materials (e.g., MXenes and graphene) to 0D, 1D, or 2D SnS nanostructures for remarkably improved performances.^[^ 28, 113, 114, 115, 116, 117, 118, 119 ^]^ In the following, some typical examples including 0D, 1D, and 2D SnS nanostructures, are introduced to demonstrate the features of SnS‐based heterostructures in a loaded model to highlight the promising applications.^[^ 28, 114, 118, 119 ^]^

Hybriding 0D SnS with other materials in a loaded model usually integrate the advantages of both individual constituents and overcome their individual shortcomings, such as serious agglomeration. In 2020, Liu et al.^[^ ^118^ ^]^ designed a new heterostructure based on 0D SnS QDs and 2D α‐SnWO_4_ NSs by a one‐pot hydrothermal method using amorphous SnWO_4_ as a precursor. The TEM image of pure α‐SnWO_4_ has a smooth and NS‐like morphology with a lateral size of sub‐micrometer (Figure 6a). After the introduction of SnS, the NS morphology of α‐SnWO_4_ maintains unchanged while its surface becomes rough because uniform QDs are well distributed on it (Figure 6b). The HRTEM image of the as‐fabricated nanocomposites shows that the interplanar *d*‐spacings of 0.28 and 0.202 nm can be indexed to the (200) and (141) planes of α‐SnWO_4_ and SnS, respectively (Figure 6b inset), and the histogram of the size of 0D SnS QDs on the surface of 2D α‐SnWO_4_ NSs displays that the average size of the SnS NPs is 2.2 nm (Figure 6c), both of which manifest the successful synthesis of the 0D SnS QD/2D α‐SnWO_4_ NS heterostructures in a loaded model. The intimate contact between 2D α‐SnWO_4_ NSs and 0D SnS QDs is conductive to the separation of photo‐induced electron–hole pairs, and thereby significantly enhance photocatalytic activity in the degradation.^[^ ^118^ ^]^ Additionally, in 2019, the combination of the reduction reaction and electrostatic assembly was employed to fabricate 0D SnS NP/2D MXene Ti_3_C_2_T_*x*_ NS heterostructures.^[^ ^113^ ^]^ Poly(diallyl dimethylammonium chloride) (PDDA) was chosen because it is a kind of cationic polymer which acted as a glue to tightly anchor 0D SnS NPs on the surface of 2D MXene Ti_3_C_2_T_*x*_ NSs (Figure 6d). It can be found that small NPs are intimately anchored on the surface of MXene NSs (Figure 6e,f), and the average size of the NPs is about 50 nm (Figure 6f). The HRTEM image shows the lattice *d*‐spacings are 0.28 and 0.44 nm, which are corresponding to (040) and (120) planes of orthorhombic SnS (Figure 6g), confirming the successful anchoring of 0D SnS NPs on the 2D MXene NSs.^[^ ^120^ ^]^ Here, 2D MXene Ti_3_C_2_T_*x*_ NSs provide a reliable substrate for the growth of 0D SnS NPs to efficiently suppress the volume expansion of SnS and prolong the cycle life in Li‐ion batteries. Other loaded model based on 0D SnS NPs, such as 0D SnS NP/2D MoS_2_ NS/N, P‐codoped C heterostructures,^[^ ^116^ ^]^ 0D SnS NP/2D MoS_2_@C heterostructures,^[^ ^121^ ^]^ 0D SnS NP/N‐doped C NS heterostructures,^[^ ^122^ ^]^ 0D SnS NP/2D reduced graphene oxide (rGO) heterostructures,^[^ 123, 124 ^]^ and 0D SnS NP/C nanocomposites,^[^ ^125^ ^]^ has also widely reported for diverse applications in recent years.

In recent years, 1D SnS nanomaterials have also been employed to prepare SnS‐based heterostructures in a loaded model and many examples, such as 1D SnS nanorod/N‐doped hierarchical porous C (N‐HPC) nanocomposites,^[^ ^64^ ^]^ 1D SnS nanobelt/graphene heterostructures,^[^ ^63^ ^]^ and 1D SnS nanowire/Au‐coated Si heterostructure,^[^ ^126^ ^]^ have been demonstrated to achieve appealing performances. For example, in 2020, Hu et al.,^[^ ^64^ ^]^ successfully synthesized 1D SnS nanorod/N‐HPC nanocomposites via phase transformation from N‐HPC/SnS_2_ by thermal annealing. It can be seen in Figure 7a that 1D SnS nanorods are horizontally loaded on the surface of N‐HPC, and the structure of 1D SnS nanorods has been further evidenced by HRTEM (Figure 7b) with an interplanar space of 0.84 nm which can be corresponding to (111) plane of orthorhombic SnS. In addition, the crystal structures of hierarchical porous C (HPC), SnS_2_/N‐HPC and SnS/N‐HPC were characterized by X‐ray diffraction (XRD), as shown in Figure 7c. The result shows that the diffraction peaks at 22.1°, 26.0°, 27.6°, 30.5°, 31.6°, 39.1°, 42.5°, 44.7°, and 45.4° can be clearly observed, which can be assigned to (110), (120), (021), (101), (111/040), (041), (210), (141), and (002) planes of orthorhombic SnS phase (PDF#01‐075‐1803).^[^ ^64^ ^]^ These XRD peaks are in good agreement with the standard SnS without any impurities, manifesting that SnS_2_ was completely reduced to SnS via thermal annealing. The conversion of 1D SnS nanorods from SnS_2_ in N‐HPC was also carried out by EDX elemental mapping (Figure 7d), confirming its successful reduction. The involvement of 1D SnS nanorods after crystal transformation makes a dramatic contribution to the suppression of volume changes and improvement of ion/electron transportation, leading to a promising anode architecture for Li‐ion batteries.^[^ ^64^ ^]^ Moreover, in 2018, a fast C‐plasma method was conducted to in situ uniformly grow hierarchical graphene on 1D SnS nanobelts using sustainable oil as the C source and partially reduced Sn as the catalyst.^[^ ^63^ ^]^ The 1D SnS nanobelts were successfully synthesized by a facile hydrothermal method. The morphology of the as‐prepared 1D SnS nanobelts could be seen in Figure 7e. A free‐standing membrane comprised of the entangled 1D SnS nanobelts with the length of dozens of micrometers and the width of ≈100 nm was formed. After C‐plasma treatment, hierarchical graphene uniformly grew on the surface of 1D SnS nanobelts (Figure 7f), leading to the strengthened flexibility even after continuous bending.^[^ ^63^ ^]^ It should be noted that metal Sn is generated during C‐plasma due to the strong plasma electron impact dissociation of the C‐based oil, evidenced by XRD pattern with the appearance of two new peaks at 43.8° and 55.3° corresponding to tetragonal Sn (JCPDS 04‐0673).^[^ ^63^ ^]^ The two interfaces among C, Sn, and SnS can be clearly seen in Figure 7g. The lattice spacing of 0.29 nm can be assigned to the (200) facet of Sn and the thickness of the Sn layer ranges from 3 to 5 nm. This unique heterostructure was also demonstrated to be capable of restricting the active nanomaterials reaggregation and stabilizing the network structure for significant improvement of overall electrochemical performance.^[^ ^63^ ^]^

Moreover, 2D SnS NSs or nanoflakes have also been introduced to fabricate SnS‐based heterostructures in a loaded model for distinctly improved performances, such as 2D SnS NS/carbonized bacterial cellulose (CBC) nanofiber heterostructures,^[^ ^114^ ^]^ 2D SnS NS/C nanofiber composites,^[^ ^28^ ^]^ 2D SnS NS/2D GeS NS heterostructures,^[^ ^108^ ^]^ 2D SnS NS/2D MoS_2_ NS heteostructures,^[^ 119, 127 ^]^ 2D SnS NS/1D aminated C heterostructures,^[^ ^115^ ^]^ 2D SnS/1D hollow C microtube heterostructures,^[^ ^111^ ^]^ 2D SnS NS/2D graphene heterostructures,^[^ ^128^ ^]^ 2D SnS NS/1D CNT heterostructures,^[^ ^112^ ^]^ etc. In 2021, Yuan et al.^[^ ^114^ ^]^ elaborately designed and rationally synthesized free‐standing film consisting of 2D SnS NSs and CBC nanofibers by a hot bath reaction followed by carbonization at high temperature (600–800 °C). Figure 8a presents the synthetic procedure of the free‐standing 2D SnS NS/1D CNC nanofiber heterostructures. Initially, bacterial cellulose (BC) aerogel obtained by freeze‐drying (Figure 8b) was immerse into a mixed ethanol solution containing SnCl_4_ and TAA, in which Sn^4+^ interacted with ‐OH in the BC nanofibers via electroadsorption and chemiadsorption to render Sn^4+^ uniformly distributed on the surface of BC nanofibers; then the sample was heated at around 80 °C to form a yellow 2D SnS_2_ NS/BC aerogel (Figure 8c); finally, the as‐obtained 2D SnS_2_ NS/BC aerogel was transferred into 2D SnS NS/CBC nanofiber heterostructure in the process of carbonization at high temperature (600–800 °C), as shown in Figure 8d. It can be seen in Figure 8b that the 3D interconnected network structure of BC aerogel has a diameter ranging from 30 to 60 nm, which is an ideal substrate for anchoring SnS_2_ nanomaterials. The as‐obtained 2D SnS_2_ NS/BC aerogel also shows a 3D interconnected network structure of BC as well as some aggregates on the surface (Figure 8c). After carbonization at 700 °C, the 3D reticulated and porous architecture of the 2D SnS NS/CBC nanofiber heterostructures maintains unchanged, and 2D SnS NSs are uniformly distributed along the CBC nanofibers (Figure 8d). The TEM image illustrates that after carbonization at 700 °C, the diameter of the 2D SnS NS/CBC nanofiber heterostructures apparently increase compared to the pure BC due to the growth of 2D SnS NSs during the transformation of SnS_2_ into SnS.^[^ ^114^ ^]^ It was demonstrated that the unique 3D network largely facilitated electrons/ions transport along all directions, significantly promoted the exposure of active sites of the 2D SnS NSs, and greatly accommodated the volume variation during lithium insertion/extraction.^[^ ^114^ ^]^ Apart from that, in 2020, Sutter et al.,^[^ ^108^ ^]^ for the first time, exploited the integration of anisotropic layered 2D SnS NS/2D GeS NS heterostructures with seamless lateral interfaces and tunable vertical design via van der Waals interaction by a PVD method using a two‐step growth process: i) 2D SnS NS was grown on a mica substrate; ii) followed by 2D GeS growth. A compact central region with small facet edges surrounded by a wide edge‐band, can be clearly seen in the NS after transferred to TEM support (Figure 8e). It should be noted that the center of the STEM sample is brighter, indicating that it has a higher average atomic number or larger thickness.^[^ ^108^ ^]^ SAED pattern taken from green region in Figure 8e identifies the wide edge‐band as single‐crystalline GeS, while the center displays a vertical overlap of GeS NS and SnS NS (Figure 8f). The HRTEM image taken from the region with an arrow marked in Figure 8e shows that the wide edge‐band GeS connected and aligned to the SnS center without any seams (Figure 8g), indicating the successful fabrication of the 2D SnS NS/2D GeS NS heterostructures in a loaded model. In addition, a vertical heterostructure composed of 2D SnS NS and 2D MoS_2_ NS was designed and fabricated based on the preferential adsorption of S powder by the precursor, MoS_2_.^[^ ^127^ ^]^ The schematic diagram of the fabrication procedure of 2D SnS NS/2D MoS_2_ NS heterostructure is presented in Figure 8h. It has three steps: i) sulfurization of raw material MoO_3_ was carried out to fabricate a MoS_2_ monolayer on a SiO_2_/Si substrate^[^ 129, 130 ^]^; ii) the preferential adsorption of S on the MoS_2_ monolayer; iii) S‐preloaded MoS_2_ was converted into the 2D SnS/2D MoS_2_ vertical heterostructure after the flow of SnCl_4_ vapor. Raman spectroscopy shows that the *A*_1g_ mode of the MoS_2_ monolayer in the 2D SnS NS/2D MoS_2_ NS heterostructure is slightly blue‐shifted (Figure 8i), suggesting the p‐type doping of the MoS_2_ monolayer by 2D SnS NS.^[^ 131, 132 ^]^ It also displays the red shift of *E*1 2g mode mainly caused by tensile strain in the MoS_2_ monolayer (Figure 8i),^[^ ^131^ ^]^ which originally results from the big lattice mismatch between two materials, MoS_2_ and SnS. Optical microscopy was also performed to characterize the morphology of the as‐fabricated 2D SnS NS/2D MoS_2_ NS heterostructure (Figure 8j). The interface between the 2D SnS NS and MoS_2_ monolayer can be clearly seen due to the contrast discrepancy. The SEM image shows that a majority of the 2D SnS NS grow on the surface of MoS_2_ monolayer, confirming that MoS_2_ monolayer as a template provides abundant SnS nucleation due to its preferential adsorption of S. The atomic structures of the SnS and MoS_2_ in the as‐fabricated 2D SnS NS/2D MoS_2_ NS heterostructure were also revealed by HRTEM (Figure 8k inset). It was manifested that this unique structure of 2D SnS NS/2D MoS_2_ NS heterostructure could not only significantly promote effective charge separation and the photoluminescence quenching of MoS_2_, but also remarkably enhance optical nonlinearities compared with individual MoS_2_ and SnS.^[^ ^127^ ^]^

#### 2.2.2 Sandwiched Model

Recently, SnS/2D nanomaterial hybrids in a sandwiched model were also intensively investigated for targeted applications, such as high‐performance solar cells,^[^ 133, 134, 135, 136 ^]^ and electronic devices.^[^ 137, 138, 139, 140 ^]^ For instance, in 2018, the facile morphology control of anisotropic SnS was realized via regulated growth kinetics during vapor transport deposition (VTD), and SnS/2D CdS heterostructure‐based thin film solar cells were fabricated to study the impact of the SnS morphology.^[^ ^135^ ^]^ It should be emphasized that the morphology of SnS grains can directly convert from plate‐like shape to cube‐like shape depending on the open‐circuit voltage (*V*_oc_) of the solar cells.^[^ ^135^ ^]^ An optimized solar cell structural morphology of the as‐fabricated soda‐lime glass (SLG)/Mo/SnS/CdS/intrinsic‐ZnO (i‐ZnO)/Al‐doped ZnO (AZO) with each layer clearly defined can be seen in Figure 9a. This structure was fabricated by three steps: i) cube‐like SnS grains obtained by the VTD technique was directly deposited on an SLG/Mo template; ii) chemical bath deposition was conducted to form a CdS buffer layer on the SnS surface; iii) i‐ZnO and AZO layers were sequentially radio‐frequency‐sputter‐deposited.^[^ ^135^ ^]^ It can be seen that there is an abrupt heterojunction formed between the SnS and CdS layers (Figure 9b), and the thickness of the middle layer CdS is measured to 70 nm. Surprisingly, a minority of MoS_2_ with a thickness of around 6 nm is also observed between substrate Mo layer and active SnS layer, confirmed by the lattice spacing of 0.61 nm indexed to (002) plane of MoS_2_ crystal, as seen in Figure 9c. The formation of MoS_2_ layer between Mo layer and SnS layer can be ascribed to the high pressure sulfurization.^[^ ^141^ ^]^ Therefore, SnS layer is well sandwiched between upper CdS layer and bottom Mo layer. Figure 9d,e shows the HRTEM image of the SnS grains between CdS layer and Mo layer. Regions X and Y marked in Figure 9d are magnified, as shown in Figure 9e, exhibiting that there are perfect SnS lattices observed near the boundary of the grains. SAED pattern of region Z marked in Figure 9d with the (100) zone axis confirms the phase purity of the cube‐like SnS grains fabricated by the VTD technique. The as‐fabricated SLG/Mo/SnS/CdS/i‐ZnO/AZO was found to maintain 98.5% of the initial efficiency (2.984%) even after stored in air for six months, suggesting the excellent quality of this anisotropic SnS‐based devices. Furthermore, Liu et al.^[^ ^138^ ^]^ reported sandwich‐like 2D SnS NS/polypyrrole (PPy) heterostructures synthesized by a pyrrole reduction and in situ polymerization using ZnSn(OH)_6_ microcubes as the Sn source and TAA as the S source. Figure 9g shows the synthetic procedure of the sandwich‐like 2D SnS NS/PPy heterostructures. First, uniform ZnSn(OH)_6_ microcubes obtained by a coprecipitation route at room temperature was used to synthesize 2D ultrathin SnS_2_ NSs in the presence of TAA by a hydrothermal method; then SnS NSs were converted from SnS_2_ NSs via in situ polymerization to prepare the sandwich‐like 2D SnS NS/PPy heterostructures. The typical TEM image of the as‐synthesized 2D SnS_2_ ultrathin NSs clearly shows smooth surfaces without any particles (Figure 9h). The TEM image of the 2D SnS NS/PPy heterostructures in Figure 9i displays a veil‐like microstructure with excellent transparency, indicating the PPy is well coated on two sides of 2D SnS NSs to form sandwich‐like NSs. The sandwich‐like structure was also supported by a representative HRTEM image of a broken composite NS (Figure 9j) due to the framework consisting of a crystalline SnS with a clear lattice spacing of 0.34 nm corresponding to (120) plane of orthorhombic SnS crystal (Figure 9j inset) and an amorphous PPy. It was also confirmed by XRD pattern in Figure 9k with complete reduction of SnS_2_ (JCPDS No. 23‐0677) by pyrrole, into SnS (JCPDS No. 39‐0354). The sandwich‐like 2D SnS NS/PPy heterostructures deliver superior cycling stability, remarkable rate capability, and a high specific capacity, holding great promise for high‐performance Li‐ion batteries.^[^ ^138^ ^]^

#### 2.2.3 Encapsulated Model

To meet the demand of new‐generation devices, macroscopically assembled architectures with secondary SnS‐based nanostructures in an encapsulated model are also expected to greatly enhance the device performances for versatile applications.^[^ 30, 69, 142, 143, 144, 145, 146, 147 ^]^ In the following, important examples on 0D, 1D, and 2D SnS nanostructures hybridizing with other components in an encapsulated model for promising performances are introduced.

So far, 0D SnS NPs have been frequently used as building blocks to achieving appealing performances due to the low‐cost fabrication, tunable functionalities, good compatibility, and excellent environmental stability.^[^ 142, 147, 148, 149, 150, 151 ^]^ For example, Choi and Kang^[^ ^147^ ^]^ developed a Sn–Mo sulfide yolk–shell (SnS–MoS_2_) composite microspheres synthesized by a one‐pot spray pyrolysis. The schematic of the yolk‐shell SnS–MoS_2_ composite microspheres can be seen in Figure 10a. First, the ultrasonic nebulizer was used to prepare droplets containing tin oxalate and MoO_3_, and sucrose; second, the SnO_2_–MoO_3_–C composite microsphere was formed through the rapid drying and decomposition of droplets, and continuous combustion and contraction resulted into the formation of SnO_2_–MoO_3_ composite microspheres; finally, the yolk–shell SnS–MoS_2_ composite microspheres were obtained by sulfidation of the as‐prepared SnO_2_–MoO_3_ composite microspheres.^[^ ^147^ ^]^ The morphologies and EDX mapping images of the as‐fabricated SnS–MoS_2_ composite microspheres after sulfidation can be seen in Figure 10b–d. The SEM image shows a clear yolk–shell SnS–MoS_2_ composite microsphere with a big crack (Figure 10b), confirmed by TEM image with a much clearer yolk–shell structure (Figure 10c). In addition, clear lattice spacings of 0.29 and 0.69 nm can be seen in the HRTEM image (Figure 10c inset), which are corresponding to the (101) and (002) planes of the orthorhombic SnS phase and hexagonal MoS_2_ phase, respectively.^[^ 152, 153 ^]^ The EDX elemental mapping images of a single yolk–shell SnS–MoS_2_ composite microsphere illustrate that SnS and MoS_2_ nanocrystals are evenly distributed all over the core and multiple shells (Figure 10d). This unique yolk–shell structure of the SnS–MoS_2_ composite microspheres was proven to have a great contribution to improve Na ion storage capability and structural stability during cycling due to the synergistic effect.^[^ ^147^ ^]^ Moreover, Choi and Kang^[^ ^154^ ^]^ also successfully fabricated SnS/C composite microspheres by the same method, one‐pot spray pyrolysis. As shown in Figure 10e, an aqueous spray solution containing Sn salt, thiourea, and polyvinyl pyrrolidone (PVP) was used to prepare SnS/C composites; and amorphous C microspheres with uniformly distributed plate‐like SnS nanocrystals (plate‐like SnS/C composite microsphere) and cubic‐like SnS nanocrystals (cube‐like SnS/C composite microsphere) were formed at 700 and 900 °C, respectively. It can be seen that the SnS/C composite microspheres prepared at 900 °C have a different morphology from that fabricated at 700 °C (Figure 10f,g). The sample prepared at 700 °C resulted in a composite with spherical morphology coated with 2D nanoplates (Figure 10f), which was also confirmed by TEM characterization (Figure 10f inset). However, the sample heat‐treated at 900 °C leads to the uniform formation of cube‐like nanocrystals with several tens of nanometers on the surface of the composite microspheres (Figure 10g), evidenced by HRTEM image (Figure 10g inset). Elemental mapping also reveals the attached SnS nanocrystals surrounding the surface of the C microspheres (Figure 10h). It was verified that the as‐prepared SnS/C composite microspheres also displayed superior reversible capacities and excellent cycle performance, which have great potential for Na‐ion batteries as anode materials.^[^ ^154^ ^]^

Apart from that, SnS‐based nanostructures containing 1D SnS nanomaterials in a capsulated model were also commonly fabricated by direct mixing or in situ formation for high‐performance or multifunctional devices, such as SnS/SiO_*x*_ core/shell nanowires,^[^ ^155^ ^]^ SnS/C nanofiber composites,^[^ 30, 144 ^]^ SnS–Sn/CNT composites,^[^ ^156^ ^]^ etc. For example, in 2020, Yang et al.^[^ ^155^ ^]^ reported well‐defined SnS/SiO_*x*_ core/shell nanowires fabricated by a CVD method using SnS powder as the source. The schematic illustration of the growth process of the well‐defined SnS/SiO_*x*_ core/shell nanowires can be seen in Figure 11a, which can be divided into six steps: i) SnS powder was heated at high temperature to generate Sn and S vapor; ii) the supersaturated Sn vapor gradually deposited on the Si surface to form liquid droplets, and subsequently etched Si substrate due to the relatively low melting point of Sn/Si alloys attached to Si surface; iii) Si substrate was oxidized by a tiny of oxygen to form SiO_*x*_; iv) owing to the confinement of the as‐synthesized SiO_*x*_ layer, Sn/SiO_*x*_ was gradually grew along 1D direction under the continuous flow of Sn; v) at the same time, SnS/SiO_*x*_ core/shell heterojunction was formed in the process of sulfuration; vi) due to the transition from liquid Sn to solid SnS, the contraction of the core volume led to the formation of the holes in the heterojunction structures. The low‐magnification SEM image shows that a large quantity of the as‐synthesized SnS/SiO_*x*_ core/shell nanowires with a curved shape are randomly distributed on the Si surface (Figure 11b). Some hollow parts can also be found in a single SnS/SiO_*x*_ core/shell nanowire in the high‐magnification SEM image (Figure 11b inset). In addition, the hollow parts of the nanowires are further confirmed by TEM characterization (Figure 11c). HRTEM image obtained from the edge region of a single nanowire also shows that the core has obvious crystal lattices indexed to orthorhombic SnS, further confirmed by SAED pattern (Figure 11c inset), and the shell is amorphous (Figure 11c inset).^[^ ^155^ ^]^ EDX elemental mapping was also carried out and the result demonstrated that O and Si were evenly distributed on the shell while Sn was only located at the core, and S was spread over the entire nanowire with markedly stronger intensity in the core (Figure 11d). Additionally, in 2019, Xia et al.^[^ ^30^ ^]^ successfully fabricated 1D SnS/C nanofiber composites via electrospinning, followed by calcination. The schematic illustration of the fabrication procedure of the 1D SnS/C nanofiber composites can be seen in Figure 11e. PVP, SnCl_2_•5H_2_O, and DMF were homogeneously mixed for electrospinning to prepare uniform nanofibers, which were then stabilized at 280 °C. Then the stabilized nanofibers were sulfuretted at 250 °C to generate SnS material in the nanofiber composites. Finally, the sulfuretted nanofibers were carbonized at high temperature (550 or 750 °C) for 1D SnS/C nanofiber composites. SEM image shows that the as‐spun SnCl_2_/PVP nanofibers have a smooth surface with an average diameter of ≈200 nm (Figure 11f). After carbonization, the diameter of the sample is shrunk to be about 130 nm with the fibrous morphology maintained (Figure 11g). The mechanical property of the as‐fabricated 1D SnS/C nanofiber composites intuitively measured shows that they can endure 30 g weight without any cracks, as shown in Figure 11h. The 1D SnS/C nanofiber composites were also characterized by EDX elemental mapping, and the result demonstrated that the elements of Sn, S, C, and N were evenly distributed in the nanofibers (Figure 11i). HRTEM image reveals clear lattice fringes with spacings of 1.87 and 3.42 Å, which can be assigned to (211) and (120) planes of orthorhombic SnS (Figure 11j), which also matches well with the XRD result.^[^ ^30^ ^]^ It was reported that these as‐obtained 1D SnS/C nanofiber composites are intimately woven into a network, which not only enhance the mechanical properties, but also promote fast ionic and electronic transport, making them have promising applications in flexible energy devices, such as Li‐ion batteries or Na‐ion batteries.^[^ ^30^ ^]^

In addition to the SnS‐based nanostructures with 0D SnS or 1D SnS encapsulated, 2D SnS nanomaterials encapsulated in the SnS‐based nanostructures have recently attracted much attention owing to its large surface‐to‐volume ratio, thickness‐dependent bandgap and anisotropic property.^[^ 36, 51, 157 ^]^ SnS‐based nanostructures containing 2D SnS NSs in a capsulated model, such as 2D SnS NS/N‐doped graphene hierarchical microflowers,^[^ ^158^ ^]^ Co,N‐modified porous C fiber@2D SnS NS array@2D graphene composites,^[^ ^69^ ^]^ 2D SnS NS/2D reduced graphene oxide (rGO) heterostructures,^[^ ^159^ ^]^ 2D SnS_2_/SnS NS heterostructure@3D graphene frameworks,^[^ ^145^ ^]^ 2D SnS NS/SnO_2_ NP/spherical graphene composites,^[^ ^160^ ^]^ are also urgent to achieve appealing performances to satisfy the growing demand of modern nanodevices. For example, in 2020, Cui et al.^[^ ^69^ ^]^ used a CVD technique to directly fabricate graphene‐like film on the surface of 2D SnS NS arrays which were supported by Co, N‐modified porous C nanofibers, abbreviated as CCF@SnS@graphene. The schematic diagram of the overall fabrication process for CCF@SnS@G can be seen in Figure 12a. First, 1D polyacrylonitrile (PAN) nanofibers containing cobalt acetate prepared by electrospinning was stabilized at 250 °C in air. Second, SnS_2_ NS arrays were grew on the stabilized PAN nanofibers by a solvothermal method, abbreviated as Co‐PAN@SnS_2_ NS composites. Finally, the Co‐PAN@SnS_2_ NS composite nanofibers were heat‐treated at 500 °C under Ar/H_2_ atmosphere to in situ convert SnS_2_ NS arrays to SnS NS arrays as well as interconnected graphene‐like C film. SEM image in Figure 12b shows that as‐prepared composites have the integrated structures with an average diameter of 600 nm. The SnS NS arrays are connected together to form a 3D network structure with clear gap space (Figure 12b inset), which is very conductive to the free transport of an electrolyte and efficient suppression of volume expansion. The low‐magnification TEM image displays that the as‐prepared CCF@SnS@G has an inner structure of C nanofiber and outer sheet‐like structure of SnS@G (Figure 12c), consistent with the SEM result. HRTEM image exhibits three clear lattice spacings of 0.29, 0.32, and 0.34 nm, which are assigned to the (101), (021), and (120) planes of orthorhombic SnS crystal, respectively (Figure 12c inset). EDX elemental mapping was also conducted to examine the distribution of different elements in the CCF@SnS@G. The result shows that elements C and N are predominately distributed in the inner nanofibers while Sn and S are enriched on the outside of nanofibers, and element Co is dispersed in the whole nanofibers (Figure 12d), confirming the successful fabrication of the unique structure of CCF@SnS@G. In addition, in 2019, Wang et al.,^[^ ^159^ ^]^ rationally fabricated oriented 2D SnS NSs confined in 2D rGO NSs via electrostatic self‐assembly followed by thermal treatment in the presence of PDDA. As shown in Figure 12e, GO and PDDA are homogeneously mixed and the electrostatic self‐assembly can be realized; then a hydrothermal approach is performed to fabricate 2D SnS_2_ NS/2D GO NS heterostructures; finally, the as‐fabricated intermediate product is directly heated at high temperature (600 °C) under Ar to simultaneously convert SnS_2_ NSs and GO NSs into SnS NSs and rGO NSs, respectively, to obtain the final product, 2D SnS NS/2D rGO NS heterostructures. TEM image shows that 2D SnS NSs with a smaller lateral size are tightly covered by 2D rGO NSs with a relatively larger lateral size, due to the contrast discrepancy (Figure 12f). Three lattice fringes of 0.210 nm, 0.279 m, and 0.171 nm can be clearly observed in Figure 12 g,h, which can be assigned to (210), (040), and (160) planes of orthorhombic SnS crystal, respectively, indicating the precursor SnS_2_ NSs were well reduced into SnS NSs in the process of thermal treatment. In addition to these lattice fringes, a clear lattice fringe corresponding to (040) plane of orthorhombic SnS (Figure 12g) is also observed in a ultrasmall NS marked in Figure 12f. These multiple lattice fringes indicates that there are many open edges at SnS NSs produced in the process of heat treatment, which do favor the Na^+^ transfer and shorten diffusion pathways, leading to superior Na^+^ storage capacity and excellent transport kinetics.^[^ ^159^ ^]^

## 3 Properties and Applications of SnS‐Based Nanostructures

Black phosphorus (BP) has achieved great progress since it was exfoliated, for the first time, into a monolayer form in 2014. SnS as one of the most popular black phosphorus analogues (BPA),^[^ 51, 161, 162, 163 ^]^ shares the same ground‐state orthorhombic structure of BP and possesses moderate thickness‐dependent bandgap and high mobility, anisotropic electrical and thermal conductivity, etc., which has great potential in a variety of fields. Notably, it is also important that SnS functionalized with other materials (e.g., polymers and other nanomaterials) is of great significance due to the synergistic effect, which holds tremendous potentials for future nanodevices in practical applications.^[^ 29, 69, 78, 103, 108, 115, 134, 164, 165, 166 ^]^ In this section, the fundamental properties (crystal structure, electronic band structure, optical property, and Raman spectroscopy) and related applications of SnS‐based nanostructures, including batteries and solar cells, catalysis (photocatalysis and electrocatalysis), optoelectronics, sensors, ferroelectrics, thermoelectrics, nonlinear properties, and biomedical applications, are clearly discussed.

### 3.1 Crystal Structure

**Figure 13:**
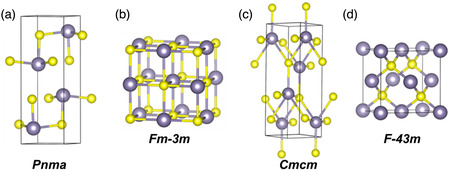
Crystal structures of SnS. Reproduced with permission.^[^ ^167^ ^]^ Copyright 2012, American Chemical Society.

The orthorhombic herzenbergite phase (space group *Pnma*) of SnS is the most commonly studied crystalline phase (**Figure** **13**a). In this structure, the Sn^2+^ ion coordinates to three S^2−^ ions, with the remaining Sn 5S^2^ electrons occupying the vacant position of a tetrahedral geometry as a lone pair of electrons.^[^ ^167^ ^]^ It was reported that bulk orthorhombic SnS had a density of 5.22 g cm^−3^ and a melting point of 882 °C.^[^ ^18^ ^]^ Single crystal of SnS typically usually shows p‐type conductivity in the range from 10^−1^ to 10^−4^ W cm^−1^, and also can change its characteristics from p‐type to n‐type.^[^ ^168^ ^]^ Other SnS phases that are of interest are *Fm‐3m* (rock salt phase), *Cmcm* (high temperature orthorhombic phase), and *F*‐*43m* (zinc blende phase), as shown in Figure 13b–d.

### 3.2 Electronic Band Structure

**Figure 14:**
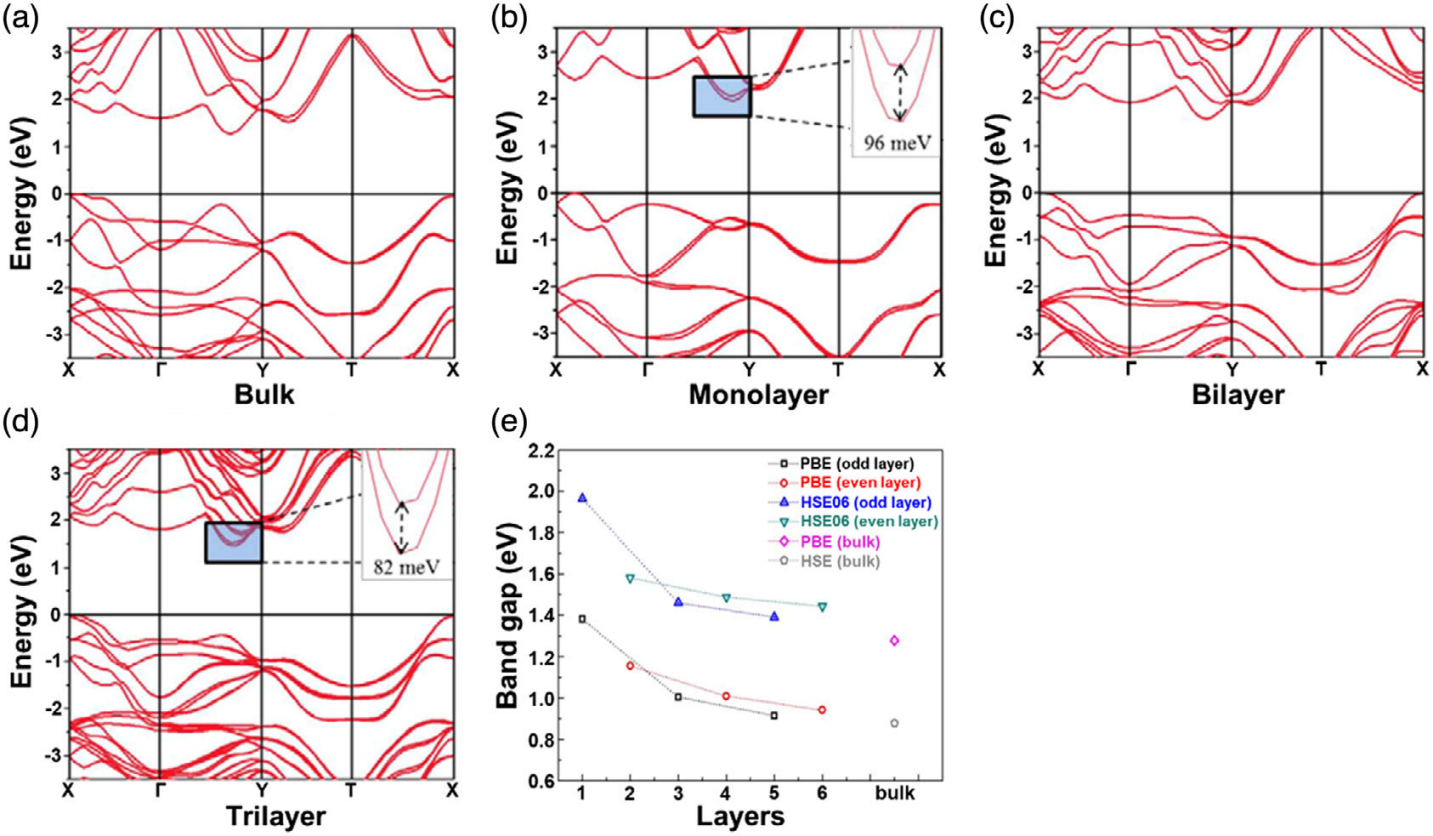
Electronic band structures of few‐layer SnS and bulk SnS: a) bulk, b) monolayer, c) bilayer, and d) trilayer SnS; e) evolution of the indirect bandgaps as a function of the layer thickness; note that the PBE‐ and HSE06‐calculated bandgaps of the bulk are marked by a pink diamond and a gray pentagon, respectively. Reproduced with permission.^[^ ^169^ ^]^ Copyright 2016, American Chemical Society.

Tunable bandgap energies are of great interest for the group‐IV monochalcogenides, such as SnS and GeS.^[^ 163, 169, 170 ^]^ Due to the underestimation of bandgaps in conventional density functional theory (DFT) calculations, the Perdew–Burke–Ernzerhof (PBE) functional calculations using the Heyd–Scuseria–Ernzerhof (HSE06) functional with 25% Fock exchange to predict a more accurate bandgap.^[^ ^169^ ^]^ The band structures of bulk, monolayer, bilayer, and trilayer SnS were calculated by HSE06 and spin–orbit coupling corrections, as shown in **Figure** **14**. It can be observed that the bulk SnS shows indirect bandgap and has the valence‐band maximum along Γ–X and the conduction‐band minimum along Γ–Y (Figure 14a). The bandgap energy (1.27 eV) of the bulk SnS matches with optical absorption measurements (1.24 eV).^[^ ^171^ ^]^ Monolayer SnS shows the largest bandgap energy of 1.964 eV (Figure 14b). The thinner the few‐layer SnS, the weaker the interlayer van der Waals interaction, and thereby the smaller the dispersion of bands, leading to a larger bandgap (Figure 14e). However, different from the normal quantum confinement effect, the bandgap of SnS does not fall monotonically with the thickness but displays an odd–even effect (Figure 14e), that is, there is a sharp drop of the bandgap energies at the conduction‐band minimum (CBM) along Γ–Y for monolayer and trilayer SnS (Figure 14b,d), but not for bulk and bilayer SnS (Figure 14a,c).

### 3.3 Optical Property

**Figure 15:**
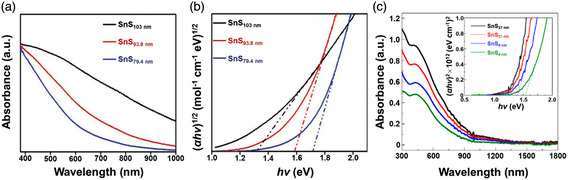
Optical property of few‐layer SnS. a) UV–vis–NIR absorption spectra of different size‐selected 2D SnS Ns in NMP solvents; and b) the corresponding Tauc plots for the calculation of bandgap energy. Reproduced with permission.^[^ ^36^ ^]^ Copyright 2018, The Royal Society of Chemistry. c) Absorbance spectra of SnS films with different thicknesses; inset showing the corresponding Tauc plot. Reproduced with permission.^[^ ^175^ ^]^ Copyright 2020, American Chemical Society.

Because of the relatively narrow bandgap energy of SnS nanomaterials, they usually show strong photoresponse behavior in the UV–vis–NIR region.^[^ 36, 67, 86, 172, 173, 174 ^]^ In 2018, our group^[^ ^36^ ^]^ employed UV–vis–NIR absorption spectroscopy to characterize the optical response of size‐selected 2D SnS_*x−y*_ obtained by a liquid cascade centrifugation (LCC) technique, as shown in **Figure** **15**a. Here, the *x* and *y* in the SnS_*x−y*_ represent the lower and upper centrifugation speeds during the LCC process, respectively. It can be observed that the absorption range becomes much wider with the increasing size of the SnS fabricated by a LPE method. Tauc plots were determined based on the results in Figure 15a, and size‐dependent bandgap energies of 1.32 eV (SnS_2–4k_), 1.59 eV (SnS_4–6k_), and 1.71 eV (SnS_6–8k_) are obtained (Figure 15b), suggesting that the bandgap energy of 2D SnS Ns could be readily controlled by simply tuning the number of SnS layers. Furthermore, in 2020, Kwon et al.^[^ ^175^ ^]^ also fabricated SnS ultrathin films by thermal disproportionation of SnS_2_ grown by CVD, and the absorption property of the SnS exhibited the same trend as our work (Figure 15c), that is, a blue shift in the absorption edge with a decrease in thickness.^[^ ^36^ ^]^ The optical bandgap of SnS with different thicknesses was also deduced from the corresponding absorption spectra using the Tauc plot (Figure 15c inset). It can be seen that bandgap energy gradually increases with the decrease of SnS thickness, in agreement with the quantum confinement effect.

### 3.4 Raman Spectroscopy

**Figure 16:**
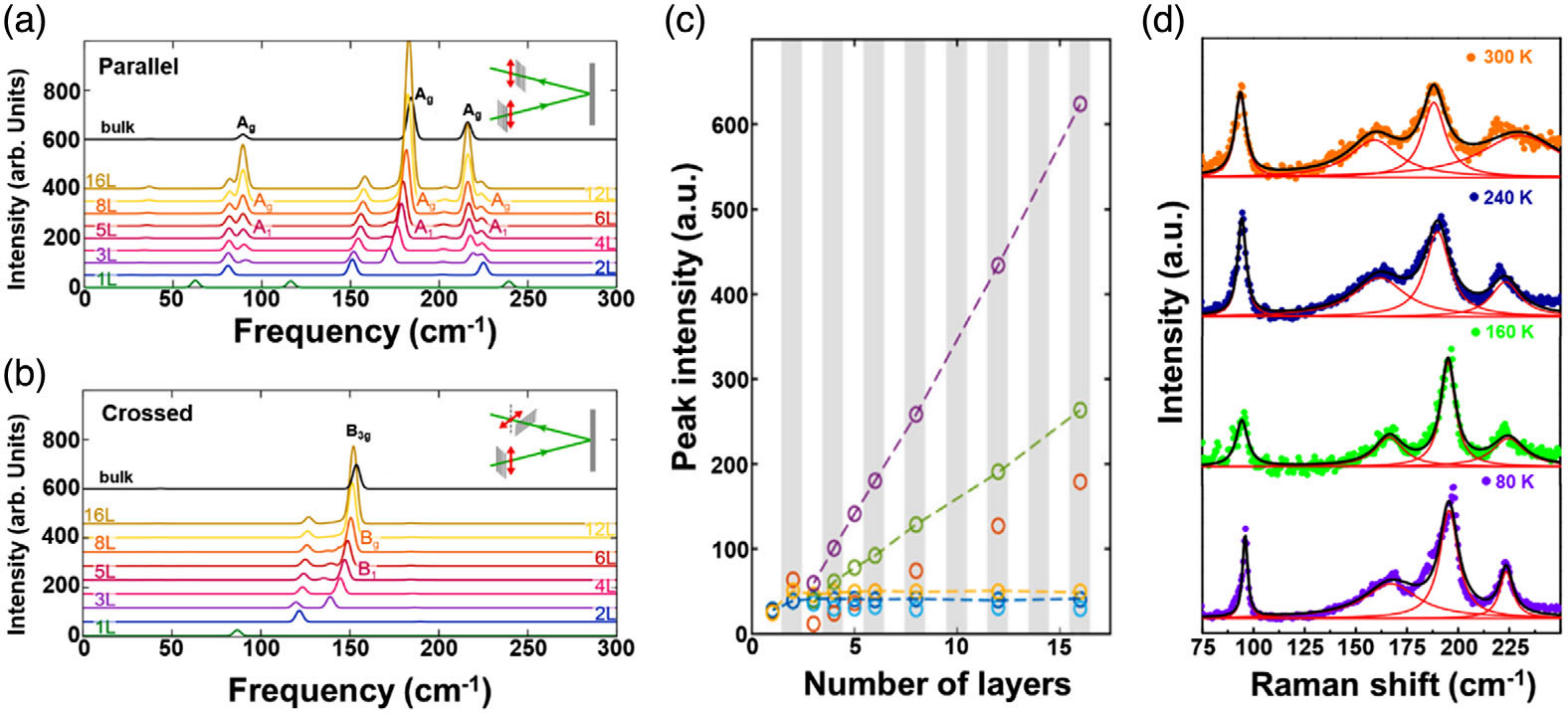
Raman spectra of few‐layer SnS. a) Thickness‐dependent Raman spectra for parallel polarization configuration (XX + YY, see inset) in the few‐layer regime (1–16 L) and for bulk SnS; b) thickness‐dependent Raman spectra for crossed polarization configuration (XY + YX, see inset); c) dependence of the peak intensity on thickness. Reproduced with permission.^[^ ^86^ ^]^ Copyright 2021, Elsevier B. V. d) Raman shift of various vibrational modes of SnS NSs at different temperatures under 532 nm laser with a power density of 3.21 mW μm^−2^. Reproduced under the terms of the CC‐BY 4.0 license.^[^ ^176^ ^]^ Copyright 2020, The Authors, published by Springer Nature. .

Raman scattering is usually employed to characterize and understand the fundamental light‐matter interaction in SnS.^[^ 86, 176, 177, 178, 179, 180, 181 ^]^ For example, in 2021, Sutter et al.,^[^ ^86^ ^]^ computed Raman spectra for SnS between 1 and 16 layers as well as bulk SnS by RGDOS approach for parallel and cross‐polarization configurations, as shown in **Figure** **16**a,b. The modes with systematic changes can be easily detected of with the change of frequency, which is usually employed to identify the number of layers. In the parallel configuration, both bulk and monolayer SnS show three Raman‐active peaks, while few‐layer SnS NSs have six clear peaks that form three groups with two peaks in a pair, who frequencies fall between those of the bulk and monolayer SnS (Figure 16a). In the crossed configuration, only one (set) of peak(s) can be observed, and the peak doublets are mainly due to the existence of bulk‐ and surface‐localized modes (Figure 16b). The collected peak intensities can be seen in Figure 16c. It shows that the modes with constant intensity belong to surface‐localized modes while those with linearly increasing intensity can be assigned to bulk modes, and thus the number of SnS layers can be identified based on the relative peak intensities. Moreover, in 2020, cryogenic Raman scattering was also carried out to investigate the phonon dynamics in ultrathin SnS NSs at a temperature range from 80 to 300 K,^[^ ^176^ ^]^ as shown in Figure 16d. All active modes (*A*_g_: 94.5, 188.1, and 221.5 cm^−1^; *B*_3g_: 161.7 cm^−1^) in Raman spectra are in good agreement with those in Sutter's work.^[^ ^86^ ^]^ It was demonstrated that all the Raman modes of the exfoliated SnS vary linearly with temperature, which is similar to other anisotropic layered materials.^[^ 182, 183 ^]^

### 3.5 Batteries and Solar Cells

As a typical BPA material, SnS has attracted great interest in energy devices because of its low‐cost, high theoretical capacity (1137 mAh g^−1^ for Li‐ion batteries and 1022 mAh g^−1^ for Na‐ion batteries), large interlayer spacing (0.559 nm), and low discharge potential.^[^ 16, 29, 30, 63, 69, 154 ^]^ More importantly, three models (loaded model, sandwiched model, and encapsulated model) on the SnS functionalized with other materials (e.g., polymers and other nanomaterials) are also clarified and demonstrated for further improvement of electrochemical performances and solar cell efficiency.

**Figure 17:**
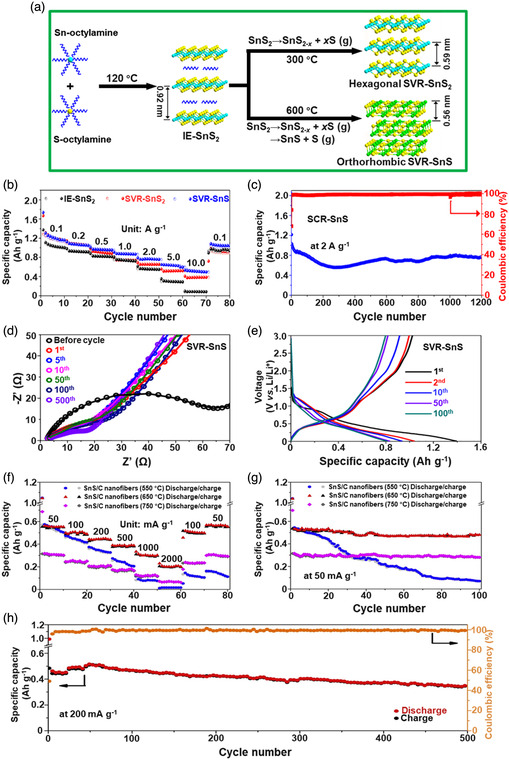
Battery performance of the SnS‐based nanostructures. a) Schematic illustration for the synthesis of IE‐SnS_2_, SVR‐SnS_2_, and SVR‐SnS; b) rate performance of these three electrodes for Li‐ion batteries; c) long‐term stability and corresponding Coulombic efficiency of the SVR‐SnS electrode at a current density of 2 A g^−1^; the initial discharge/charge cycle was conducted at a small current density of 50 mA g^−1^; d) Nyquist plots of the SVR‐SnS electrode at various cycles, and e) representative discharge/charge curves of the SVR‐SnS electrode at a current density of 1 A g^−1^. Reproduced with permission.^[^ ^188^ ^]^ Copyright 2020, Elsevier B.V. f) Rate performance of SnS/C nanofibers with different carbonization temperatures at various current densities from 50 to 4000 mA g^−1^; g) cycling performance of 1D SnS/C nanofibers at a current density of 50 mA g^−1^; and h) long‐term cycling performance for SnS/C nanofibers (650 °C). Reproduced with permission.^[^ ^30^ ^]^ Copyright 2019, Elsevier B.V.

With respect to the electrochemical performances in batteries, although pure SnS nanostructures, such as 0D SnS NPs,^[^ ^31^ ^]^ 1D SnS nanostructures,^[^ 62, 66 ^]^ 2D SnS NSs or nanoplates,^[^ 153, 184, 185 ^]^ 3D SnS nanoflowers or yolk–shell microstructures,^[^ 32, 99, 186 ^]^ have made considerable progress in the field of batteries, such as Li or Na‐ion batteries and Li‐S batteries, yet its low electronic conductivity, large volume expansion and poor cycling stability during charging and discharging, greatly limit its practical applications. To this end, a number of researches has focused on the functionalization of SnS with graphene,^[^ 69, 101, 124, 137, 158, 159 ^]^ CNTs,^[^ 111, 112, 156, 187 ^]^ C fibers,^[^ 28, 30, 146 ^]^ conductive polymers,^[^ ^138^ ^]^ MoS_2_ NSs,^[^ 116, 147, 150 ^]^ etc., to achieve outstanding electrochemical performances. For example, in 2020, 2D SnS nanoplates modified with abundant S vacancies were synthesized by a self‐template strategy for Li‐ion batteries as advanced anode materials.^[^ ^188^ ^]^ The synthetic route of 2D SnS nanoplates modified with abundant S vacancies can be seen in **Figure** **17**a, where interlayer‐expanded SnS_2_ nanoplates, hexagonal S‐vacancy‐rich 2D SnS_2_ nanoplates, and orthorhombic 2D SnS nanoplates are abbreviated as IE‐SnS_2_, SVR‐SnS_2_, and SVR‐SnS, respectively. The electrochemical performances of these as‐synthesized three samples as anode materials for Li‐ion batteries were investigated. Rate capabilities of the three samples display that the sample SVR‐SnS has a remarkably outperformed capacity retention of 41.6% compared to those of SVR‐SnS_2_ (31.7%) and IE‐SnS_2_ (8.3%) when current density rises from 0.1 to 10 A g^−1^ (Figure 17b). Furthermore, the capacity of the SVR‐SnS electrode can retain 89.0% of its initial capacity when the current density returns to 0.1 A g^−1^. It should be noted that the SVR‐SnS electrode can still deliver a reversible discharge capacity of 765 mAh g^−1^ after 1,200 cycles at 2 A g^−1^ (Figure 17c), which was considered to be the best cycling performance in the SnS‐based electrodes.^[^ ^188^ ^]^ It was demonstrated that the excellent cycling performance and superior rate capability of SVR‐SnS electrode could be attributed to these following three aspects: i) due to the change of electronic states after SnS nanoplates were modified with abundant S vacancies, the bandgap of SVR‐SnS reduced and its electronic conductivity increased; ii) the formation of localized built‐in electric field further accelerated the Li^+^ diffusion^[^ 189, 190 ^]^; iii) abundant S vacancies served as active sites for further improvement of the electrochemical reaction.^[^ 191, 192, 193 ^]^ It can be observed in Figure 17c that there is a capacity fading in the first 280 cycles for the SVR‐SnS electrode, which can be ascribed to the relatively low reversibility of the electrochemical reaction and the pulverization of the SVR‐SnS nanoplates. Nyquist plots of the SVR‐SnS electrode before cycling and after various cycles verifies that its electrical conductivity gradually increases and the electrochemical reaction kinetics improves with the discharging/charging cycling (Figure 17d). The galvanostatic charge/discharge profiles of the SVR‐SnS electrode in the cutoff voltage from 0.01 to 3.0 V at 1 A g^−1^ show that a relatively large initial Coulombic efficiency of 73.4% is achieved as well as a reversible capacity of 1022 mAh g^−1^ (Figure 17e). After 100 cycles, a discharge capacity of 807 mAh g^−1^ is still retained, indicating that the SVR‐SnS electrode has better electrochemical performances and reversibility. Moreover, in 2019, Xia et al.^[^ ^30^ ^]^ successfully fabricated a SnS/C film via electrospinning for both Li‐ion batteries and Na‐ion batteries as a free‐standing anode. The fabrication process for the 1D SnS/C nanofibers can be seen in Figure 11e. The SnS/C nanofibers carbonized at 550, 650, and 750 °C, are abbreviated as SnS/C nanofibers (550 °C), SnS/C nanofibers (650 °C), and SnS/C nanofibers (750 °C), respectively. The rate capabilities of the three 1D SnS/C nanofiber samples at different current densities (50–2000 mA g^−1^) show that the 1D SnS/C nanofibers (650 °C) electrode displays the best rate performance compared to the other two electrodes (Figure 17f). In addition, the discharge capacities of SnS/C nanofibers (650 °C) decrease as the current density increases, while they can totally recover when the current density goes back, which suggests that the SnS/C nanofibers holds great promises in the practical applications of high‐power Na‐ion batteries. The cycle performances of these three electrodes at 50 mA g^−1^ in Na‐ion batteries exhibit that the SnS/C nanofibers (650 °C) electrode can maintain 90.2% of its initial discharge capacity, comparable to that of SnS/C nanofibers (750 °C) but significantly superior to that of SnS/C nanofibers (550 °C) due to its low electronic conductivity (Figure 17g). Regardless of similar cycling stabilities of the SnS/C nanofibers (650 °C) and SnS/C nanofibers (750 °C), SnS/C nanofibers (750 °C) delivers an apparently lower capacity (281 mAh g^−1^) than 481 mAh g^−1^ for SnS/C nanofibers (650 °C). In addition, the galvanostatic discharge‐charge measurement of the SnS/C nanofibers (650 °C) under a very high current density of 200 mA g^−1^ was performed to characterize the long‐term cycling stability, as shown in Figure 17h. After 500 cycles, the SnS/C nanofibers (650 °C) electrode can still deliver a discharge capacity of 349 mAh g^−1^, which is considered to be one of the most high‐performance SnS‐based electrodes for Na‐ion batteries as compared to bare SnS,^[^ ^194^ ^]^ multiwall CNTs/SnS composites,^[^ ^195^ ^]^ and graphene/SnS composites.^[^ ^185^ ^]^ Similarly, the as‐prepared SnS/C nanofibers (650 °C) also have ultralong cycle life and high capacity in Li‐ion batteries.

**Figure 18:**
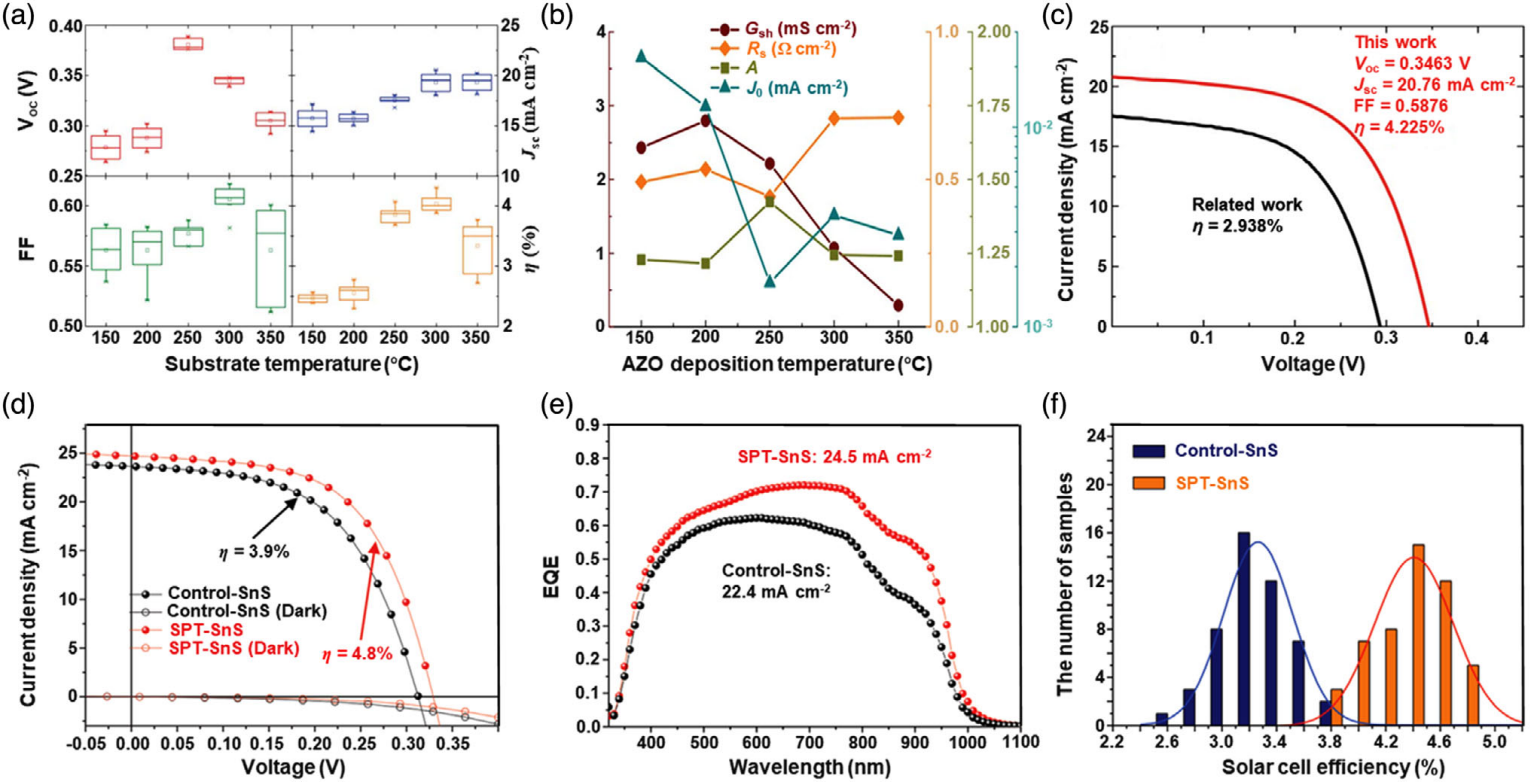
Application of the SnS‐based nanostructures in solar cells. a) A box plot for device performance of all eight cells for each of the SnS/CdS TFSCs at various AZO deposition temperatures; for clarity, fully shunted cells were excluded; b) the calculated diode parameters, *G*_sh_, *R*_s_, *A*, and *J*_0_, as a function of the AZO film deposition temperature; c) current density‐voltage curves for the champion cell (*η* = 4.225%). Reproduced with permission.^[^ ^198^ ^]^ Copyright 2020, The Royal Society of Chemistry. Comparison of the control‐SnS and SPT‐SnS solar cells: d) solar cell efficiencies, e) EQE, and f) reproducibility of the solar cell efficiency. Reproduced with permission.^[^ ^134^ ^]^ Copyright 2019, Wiley‐VCH.

In terms of the performances in thin‐film solar cells, earth‐abundant SnS has shown intriguing properties in recent years due to its suitable bandgap, high absorption coefficient, and conductivity.^[^ 36, 51, 196, 197 ^]^ Moreover, a number of researches have focused on the optimization of solar cell efficiency by controlling device structures, such as Mo‐coated soda‐lime glass (SLG/Mo)/SnS/CdS/i‐ZnO/AZO/Al,^[^ ^198^ ^]^ In‐doped SnO_2_/ZnO/Zn(O,S):N/SnO_2_/SnS/Mo/glass,^[^ ^181^ ^]^ FTO/TiO_2_/SnS/Au,^[^ ^134^ ^]^ fluorine‐doped tin oxide (FTO)/planar‐TiO_2_ or mesoporous‐TiO_2_/SnS/P3HT/PEDOT:PSS/Ag,^[^ ^199^ ^]^ ITO/Zn_1*−x*_Mg_*x*_O/SnS/Cu,^[^ ^200^ ^]^ and Al/ZnO:Al_2_O_3_/ZnO/CdS/SnS/Mo/SLG.^[^ ^201^ ^]^ In 2020, Cho et al.^[^ ^198^ ^]^ reported VTD SnS/CdS heterojunction solar cells whose final device configuration was SLG/Mo/SnS/CdS/i‐ZnO/AZO/Al, with a highest efficiency of 4.225% and excellent long‐term stability even over two years. The solar cell output parameters, such as open‐circuit voltage (*V*_oc_), short‐circuit current density (*J*_sc_), fill factor (FF) and power conversion efficiency (*η*), as a function of substrate temperature can be seen in **Figure** **18**a. The thin‐film solar cells (TFSCs) is largely dependent on the AZO substrate deposition temperature, that is, the TFSCs show a relatively poor device performance at substrate (AZO) deposition temperature of 150 °C (*V*_oc_: 0.295 V, *J*_sc_: 14.88 mA cm^−2^, FF: 0.584, and *η*: 2.57%); then reach an improved device performance at 300 °C (*V*_oc_: 0.346 V, *J*_sc_: 20.53 mA cm^−2^, FF: 0.604, and *η*: 4.30%); and device performance degrades as the substrate deposition temperature further increase to 350 °C (*V*_oc_: 0.313 V, *J*_sc_: 20.08 mA cm^−2^, FF: 0.601, and *η*: 3.77%) due to the Cd diffusion at 350 °C. The overall device performance is limited due to the relatively smaller *J*_sc_ and FF. Surprisingly, the TFSCs with AZO films deposited at 300 °C exhibit the highest efficiency, although they display slightly reduced *V*_oc_. The influence of the substrate deposition temperature on the diode quality of SnS/CdS TFSCs was analyzed, and the diode parameters such as shunt conductance (*G*_sh_), series resistance (*R*_s_), ideality factor (*A*), and dark saturation current density (*J*_0_) were plotted against the AZO deposition temperature, as shown in Figure 18b. There is a clear decline in *G*_sh_ observed with the increase of the AZO deposition temperature, which can be mainly ascribed to the enhanced properties of CdS due to densification during annealing. *R*_s_ maintain almost unchanged below 250 °C, while display a slightly increase when temperature ranges from 250 to 350 °C. It can be also observed that the *A* value is nearly similar in the studied temperature range. As for *J*_0_, there is a sharp reduction in the temperature ranging from 150 to 250 °C, indicating a dramatic decline in interface defects and nonradiative recombination which is also in good agreement with the highest *V*_oc_ (0.389 V) achieved for the TFSCs deposited at 250 °C; moreover, slightly enhancement is achieved when AZO deposition temperature increases from 250 to 350 °C. In addition, independently measured current density–voltage curves of the TFSCs with AZO films deposited at 300 °C verifies that both the current density and voltage exhibit an obvious enhancement compared to the previously reported work.^[^ ^135^ ^]^ It was also concluded that a much thinner CdS (45 nm) may result into the improved *η* (4.225%) in the wavelength range of 400 to 500 nm compared to the previously reported work (*η* = 2.938%) with a thicker CdS (70 nm), as shown in Figure 18c. It is envisioned that the decrease of bulk and interface defect densities of SnS absorbers plays an important role in achieving high efficiency of the SnS‐based TFSCs. Additionally, a high‐performance TiO_2_/SnS heterostructure‐based solar cells fabricated by a solution of SnCl_2_ and thiourea was designed and exhibited the highest *η* (≈4.8%) in the reported solution‐processed solar cells.^[^ ^134^ ^]^ The fabrication procedure for the TiO_2_/SnS heterostructure‐based solar cells is presented as follows: i) the solution containing a predetermined amount of SnCl_2_ and thiourea is directly deposited on nanostructured TiO_2_ (ns‐TiO_2_)/blocking layer TiO_2_ (BL‐TiO_2_)/FTO by spin coating; ii) the sample is annealed at 350–450 °C in air, followed by deposition of Au electrodes to form the final device configuration of FTO/BL‐TiO_2_/ns‐TiO_2_/SnS/Au.^[^ ^134^ ^]^ The comparison of current density–voltage curves of the devices before and after SnCl_2_ post‐treatment (SPT) under 1 sun radiation conditions shows that the efficiency of SnS solar cell (control‐SnS, *η* = 3.9%) can be further increased by SnCl_2_ post‐treatment (SPT‐SnS, *η* = 4.8%), as shown in Figure 18d. The FF was investigated to understand the effect of SPT on the solar cell performance, and the result displayed that SPT‐SnS owned a lower FF (7.5) compared to that of control‐SnS (8.5), suggesting its less recombination. External quantum efficiency (EQE) of 22.4 mA cm^−2^ was characterized at a photocurrent density in the wavelength range of 350 to 1100 nm (Figure 18e), in line with the current density in Figure 18d. It should be pointed out that the EQE for SPT‐SnS increases by almost 8.6% in the range of 500 to 850 nm, compared with that in the mid‐ to near‐infrared region. More importantly, statistical analysis of the efficiency of the as‐fabricated control‐SnS and SPT‐SnS solar cells shows that the SPT not only improves the solar cell efficiency but also enhances the reproducibility (Figure 18f). It was also proved that the improved soar cell performance was predominately attributed to the much denser and more uniform SnS thin film after SPT as well as the in situ reduction of defects with the penetration of Cl^−^ into the SnS lattice.^[^ ^134^ ^]^ It is anticipated that this solution process can provide a new direction for further improvement of the solar cell efficiency for promising solar cell materials.

### 3.6 Catalysis

**Figure 19:**
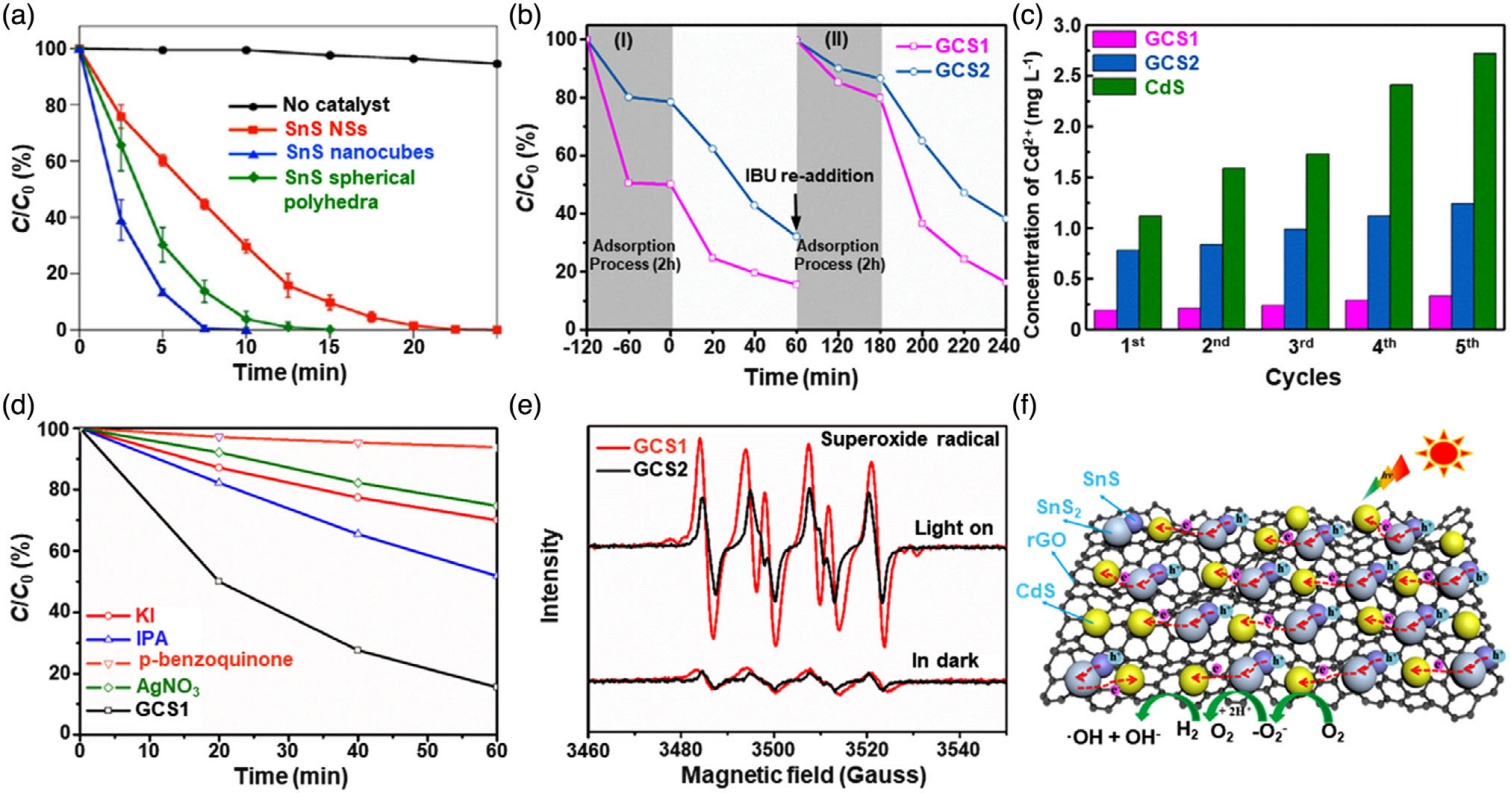
Photocatalytic performance of the SnS‐based nanostructures. a) Determination of the activity of SnS NSs, nanocubes, and spherical polyhedra in the photocatalytic degradation of MB as a function of time. Reproduced with permission.^[^ ^50^ ^]^ Copyright 2013, American Chemical Society. Adsorption and photocatalytic activity evolution for b) GCS1 and GCS2; c) cycling performance of the concentration of Cd^2+^ for each run in the reaction solution of CdS, GCS1, and GCS2; d) the trapping experiments of reactive species, DMPO spin‐trapping ESR spectra recorded with GCS1 and GCS2 in e) methanol dispersion (for DMPO‐^•^ $O_{2}^{-}$); f) possible photogenerated charge transfer mechanism on the GCS1 composite; the direction of the arrows represents the transfer pathway of electrons and the opposite direction indicates the migration route of holes. Reproduced with permission.^[^ ^208^ ^]^ Copyright 2020, Elsevier B.V.

SnS, as an emerging BPA semiconductor material, holds great promise in the community of photo/electrocatalysis due to its unique properties: i) thickness‐dependent indirect bandgap from 1.07 to 1.25 eV and direct bandgap from 1.30 to 1.39 eV^[^ 102, 202, 203 ^]^; ii) earth‐abundant 2D binary IV–VI compound with low‐cost; iii) ultrahigh carrier mobility (10,000–38,000 cm^2^ V^−1^ s^−1^)^[^ ^169^ ^]^; and iv) excellent environmental stability.^[^ 5, 115 ^]^ In addition, to further exploit the potential of SnS in photo/electrocatalysis, SnS has been usually hybridized with other functional nanomaterials for preparing efficient co‐catalysts, such as CuS@β‐SnS nanocomposites,^[^ ^22^ ^]^ SnS/rGO heterostructures,^[^ ^140^ ^]^ SnS/aminated‐C composites,^[^ ^115^ ^]^ 0D SnS QD/2D α‐SnWO_4_ NS heterostructures,^[^ ^118^ ^]^ SnS/SnS_2_ heterostructures,^[^ ^204^ ^]^ SnS/P‐doped g‐C_3_N_4_ nanocomposites,^[^ ^205^ ^]^ SnS/SnO_*x*_ heterostructures,^[^ ^206^ ^]^ etc. For example, colloidal SnS nanocubes, SnS spherical polyhedral, and SnS NSs were successfully fabricated and demonstrated to have excellent photocatalytic degradation activity of methylene blue (MB).^[^ ^50^ ^]^ As shown in **Figure** **19**a, it can be found that both SnS nanocubes and SnS spherical polyhedral are distinctly better than SnS NSs, which can be attributed to the higher surface area of 0D SnS nanocubes and 0D spherical polyhedral with statistically higher activities on a mass basis. However, 0D SnS nanocubes show much faster photocatalytic activity of MB than 0D spherical polyhedral, although 0D SnS nanocubes and 0D spherical polyhedral almost have the same size as well as similar absorption. This fact indicates the shape of SnS nanomaterials has a great influence on the photocatalytic activity, similar to other semiconductor nanomaterials.^[^ 33, 34, 207 ^]^ It also implies that the (011) facet by which 0D SnS nanocubes are substantially bound is more active in the photodegradation of MB than the exposed (010) and (001) faces in a large quantity on the 0D spherical polyhedral. Moreover, in 2020, a new kind of photocatalyst based on two Sn valence states (II and IV), CdS–SnS–SnS_2_/rGO heterostructure, was successfully fabricated by a facile solvothermal method.^[^ ^208^ ^]^ For convenience, the as‐prepared CdS–SnS–SnS_2_/rGO heterostructure with a rGO loading content of 0 and 6.0 wt% is abbreviated as CS and GCS1, respectively, while CdS‐SnS_2_/rGO heterostructure with the same loading of rGO (6.0 wt%) synthesized by a similar method except for the introduction of SnS, is abbreviated as GCS2. The photocatalytic activity for the degradation of an important model pollutant (ibuprofen, IBU) was investigated under visible light. The adsorption and photodegradation efficiencies of GCS1 and GCS2 were measured to understand the importance of SnS component in the heterostructures, as shown in Figure 19b. In the stage I, both of the samples show obvious absorption of IBU in the dark, and the absorption efficiency of GCS1 can reach ≈50% in 2 h while that of GCS2 comes up to ≈20%. After exposed to visible light for 1 h, 85% of the initial IBU has been degraded for GCS1, significantly superior to that for GCS2 (68%). Moreover, after the first adsorption and photodegradation, fresh IBU was then added into the initial solution for a second measurement. As displayed in Figure 19b, in stage II, near 80% adsorption efficiency is observed for GCS1, which is distinctly larger than that in the stage I (≈50%). After second exposure to visible light for 1 h, it can be seen that 85% of IBU has been removed for GCS1, remarkably outperforming that for GCS2 (62%), indicating that the inherent merits of SnS and double heterojunctions after the introduction of SnS are indeed beneficial to the improvement of photocatalytic performance. Furthermore, due to the nontoxicity of Sn^4+^ and Sn^2+^, the toxicity of the as‐fabricated composites can be easily characterized by simply measuring the concentration of Cd^2+^ by inductive coupled plasma emission spectrometer, as shown in Figure 19c. It can be found that GCS1 shows the best photocorrosion efficiency of CdS after five cycles compared to individual CdS and GCS2, indicating that the introduction of SnS has a great effect on photocorrosion efficiency of metal ions such as Cd^2+^, and environmental stability. Moreover, different reactive species were chosen to understand the excellent photocatalytic efficiency of IBU for GCS1. The reactive species of KI, isopropanol (IPA), p‐benzoquinone (p‐BQ), and AgNO_3_ was added to selectively quench holes (*h* ^+^), hydroxyl radicals (^•^OH), superoxide anion radicals ^•^ $O_{2}^{-}$, and electrons (*e* ^−^), respectively. It can be deduced from the trapping curves in Figure 19d that ^•^ $O_{2}^{-}$ plays a vital role in the IBU degradation due to its significant suppression effect while other species (*h* ^+^, ^•^OH, and *e* ^−^) also take part in the IBU degradation. The importance of ^•^ $O_{2}^{-}$ in the IBU degradation was also evidenced by an electron spin resonance spin‐trap technique using 5,5‐dimethyl‐1‐pyrroline *N*‐oxide (DMPO), as shown in Figure 19e. Much stronger peak intensities under visible light can be observed compared to that in the dark, confirming that ^•^ $O_{2}^{-}$ play a decisive role in the degradation of IBU. The peak intensity of GCS1 is obviously stronger than that of GCS2, which is possibly attributed to the double heterojunction after the introduction of SnS. The proposed mechanism of the composites in the photocatalytic degradation can be seen in Figure 19f. The introduction of SnS in this composite can form another heterojunction between SnS and SnS_2_ except that between SnS_2_ and rGO, and rGO in this composite acts as an electron transfer bridge, which can largely enhance the transfer efficiency of the accumulated electrons from SnS edge to SnS_2_ at first, and then to CdS for accelerating the separation of photogenerated *h* ^+^, and *e* ^−^, and thereby efficiently promotes the photocatalytic activity.

**Figure 20:**
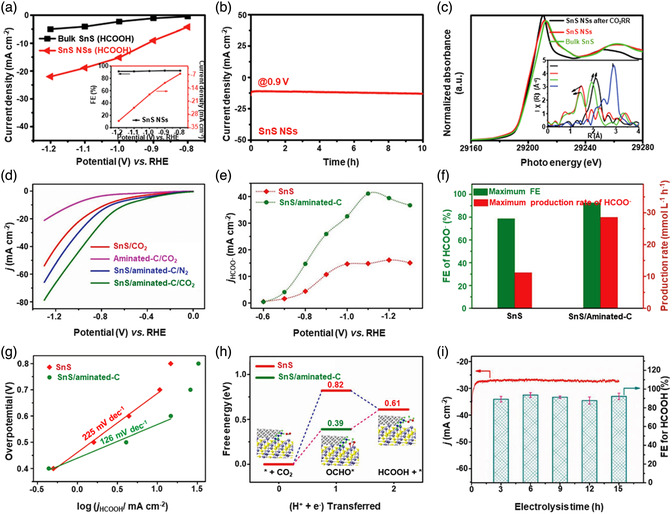
Electrocatalytic performance of the SnS‐based nanostructures. a) Partial current densities (HCOOH) for the CO_2_RR of bulk SnS and SnS NSs with partial current density and HCOOH F.E. of SnS NSs (inset); b) chronoamperometry measurements of SnS NSs at 0.9 V; and c) Sn K‐edge XANES and EXAFS (inset) spectra for bulk SnS and SnS NSs before and after continuous CO_2_RR tests. Reproduced with permission.^[^ ^5^ ^]^ Copyright 2020, The Royal Society of Chemistry. d) LSVs of SnS, aminated‐C, and SnS/aminated‐C composites in CO_2_/N_2_‐saturated 0.5 m KHCO_3_ electrolyte; e) partial current density of formate production for SnS and SnS/aminated‐C composites; f) comparison of maximum formate F.E. and formate production rate of SnS and SnS/aminated–C composites; g) Tafel plots of SnS and SnS/aminated‐C; h) calculated free‐energy diagram of electrocatalytic reduction of CO_2_ to formate over SnS and SnS/aminated–C composites; and i) stability test of SnS/aminated–C composites at an electrolysis potential of −0.9 V. Reproduced with permission.^[^ ^115^ ^]^ Copyright 2020, Wiley‐VCH.

Apart from widely photocatalytic applications, recent studies have also shown that SnS‐based nanostructures have great potential in electrocatalysis.^[^ 5, 22, 102, 115 ^]^ For example, in 2020, Chen et al.^[^ ^5^ ^]^ reported that electrochemically exfoliated 2D ultrathin SnS NSs with a relatively large lateral size of 1.0 μm and a very thin thickness of only about 5.0 nm, exhibited a good electrocatalytic activity toward CO_2_ reduction reaction (CO_2_RR). A three‐electrode system in 0.5 m KHCO_3_ electrolyte was used to investigate the electrocatalytic properties of the as‐fabricated SnS NSs. The as‐fabricated 2D SnS NSs exhibit significantly larger current densities in the potential range from −1.2 to −0.8 V, compared to those of bulk SnS (**Figure** **20**a), indicating an excellent HCOOH selectivity of the 2D SnS NSs. A maximum faradaic efficiency (F.E.) value of 82.1% of the as‐fabricated 2D SnS NSs for HCOOH production at −1.1 V can be observed, which is about 2.78 times higher than that of bulk SnS (29.5%) under the same conditions (Figure 20a inset). The Tafel slope of the 2D SnS NSs was measured to be as low as 180 mV dec^−1^, approaching the theoretical value (200 mV dec^−1^), significantly lower than that of the bulk SnS (357 mV dec^−1^), suggesting that the 2D SnS NSs have favorable reaction kinetics. Chronoamperometry measurement was conducted to investigate the long‐term stability of the 2D SnS NSs for CO_2_RR at −0.9 V with the current density remained at 13 mA cm^−2^ for 10 h, as shown in Figure 20b. A negligible change in the current density can be found during measurement, indicating that the 2D SnS NSs have an excellent stability for practical applications. To trace the structural evolution of the 2D SnS NSs before and after CO_2_RR measurements, the post Sn K‐edge X‐ray absorption near‐edge structure (XANES) and the extended X‐ray absorption fine structure (EXAFS) were carried out, as shown in Figure 20c. It can be seen that there are no obvious differences for the Sn absorption edge positions before and after CO_2_RR between 2D SnS NSs and bulk SnS, suggesting that all the samples have the same oxidation state of Sn^2+^. In addition, from Fourier transform EXAFS results (Figure 20c inset), there are clear Sn^2+^‐O peaks observed in 2D SnS NSs and bulk SnS due to oxygen groups adsorption to form the Sn^2+^‐O coordination. However, after CO_2_RR, the disappearance of the Sn^2+^‐O peak and remarkably enhanced intensity of the Sn‐S peak of 2D SnS NSs are clearly observed, revealing that the electrocatalysis efficiently promotes the desorption of adsorbed O groups on the Sn surface, leading to more exposed active sites of Sn–S. Moreover, in 2020, hollow nanotubes consisting of 2D SnS NSs were modified by amino‐functionalized C for CO_2_RR by a sacrificial template technique.^[^ ^115^ ^]^ 2D SnS NSs coated with aminated C layers uniformly grew on the surface of MoO_3_ nanorods by a facile hydrothermal method, followed by MoO_3_ nanorods selectively etched away by NH_3_·H_2_O to prepare SnS/aminated–C composites. Linear sweep voltammetry (LSV) curves measured in N_2_ and CO_2_‐saturated electrolyte (Figure 20d) show that a larger current density has achieved for the SnS/aminated–C composites under CO_2_ than N_2_‐saturated electrolyte. Note that in CO_2_‐saturated electrolyte, the SnS/aminated–C composites display a more positive onset potential and a larger current density compared to those of individual SnS and aminated C. Electrocatalytic activity of CO_2_RR evaluated by the potentiostatic electrolysis (Figure 20e) shows that SnS/aminated–C composites also display a remarkably higher formate partial current density ($J_{\text{HCOO}}^{-}$, 41.1 mA cm^−2^) than that of pristine SnS (13.7 mA cm^−2^). As a result, the formate production rate for the SnS/aminated–C composites can reach up to 28.6 mmol L^−1^ h^−1^ (Figure 20f), suggesting that the SnS/aminated–C composites are ideal electrocatalysts for CO_2_RR to formate. Tafel plots (overpotentials for formate production vs $\text{log}J_{\text{HCOO}}^{-}$) calculated from Figure 20e illustrate that in the relatively low overpotential region, the as‐fabricated SnS/aminated–C composites exhibit a much lower slope value (126 mV dec^−1^) than that of pristine SnS (225 mV dec^−1^), indicating a faster reaction rate of the SnS/aminated–C composites (Figure 20g). It should be pointed out that the pretty low slope value of the SnS/aminated–C composites (126 mV dec^−1^, close to 118 mV dec^−1^) verifies that the initial step with one electron transfer is the rate‐determining step. To elucidate the mechanism of CO_2_RR, density functional theory (DFT) theoretical calculations were also carrier out using the computational hydrogen electrode method. As shown in Figure 20h, there are two‐step electron‐proton coupling transfer steps in the electrocatalytic reduction of CO_2_ to HCOOH. For both pristine SnS and the SnS/aminated–C composites, the free energy (Δ*G*) which is essential to form OCHO* is higher than that of the formation of adsorbed HCOOH, suggesting the formation of OCHO* is the potential‐limiting step, in good agreement with the Tafel analysis in Figure 20g. However, the Δ*G* to form OCHO* of the SnS/aminated–C composites reduce by 0.43 eV, which is even lower than that for COOH*, implying that they have an excellent selectivity to the formation of formate. A continuous electrolysis experiment lasting 15 h for the SnS/aminated–C composites shows that the current density and the F. E. for the formate remain nearly stable (Figure 20i), indicating that they also hold great promise for practical applications.

### 3.7 Optoelectronics

**Figure 21:**
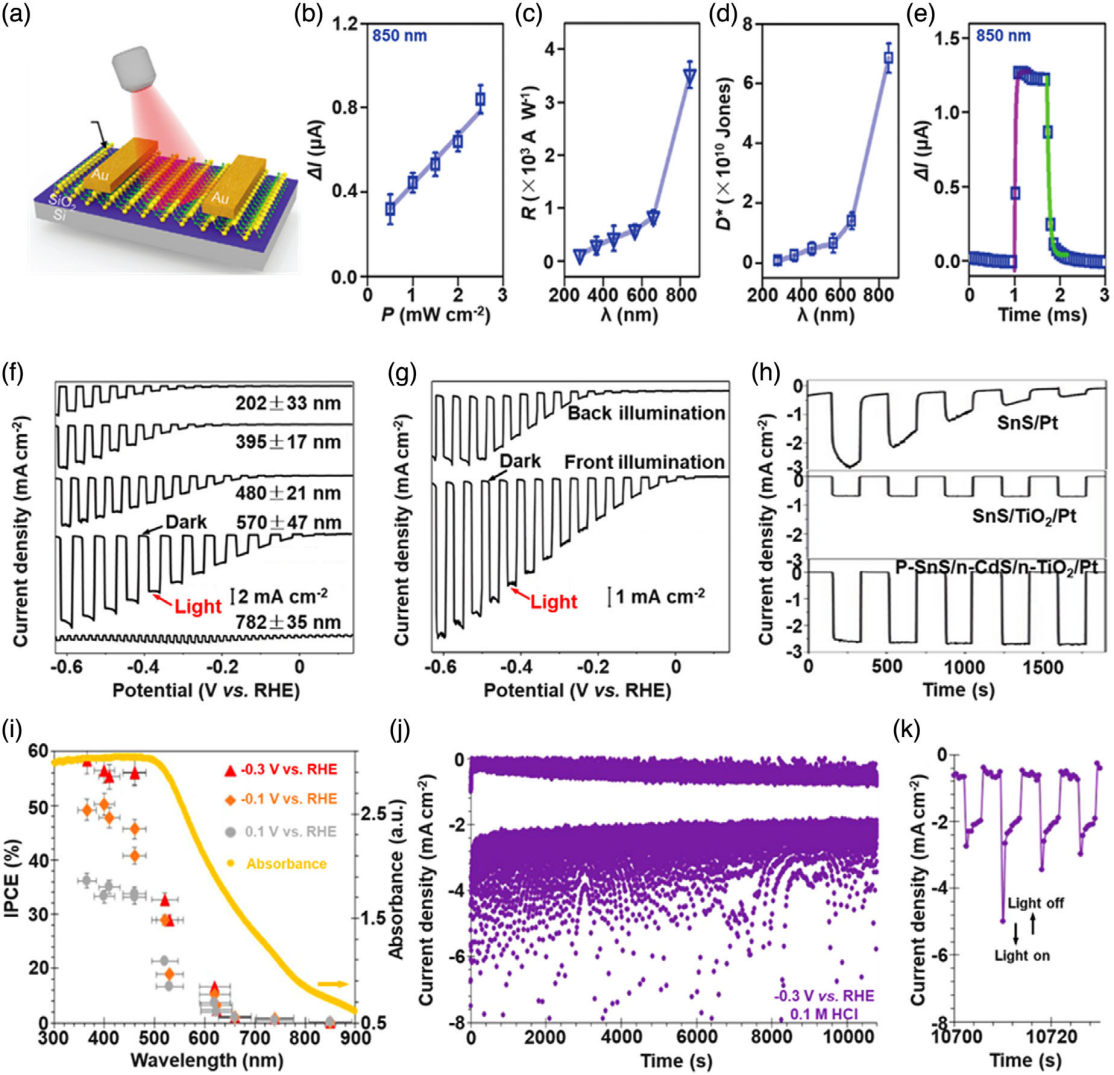
Photoresponse behavior of the SnS‐based nanostructures. a) Schematic illustration of 2D SnS NS‐based photodetector fabricated on a SiO_2_/Si substrate; b) photocurrent at different power intensities of multiple‐unit cell thick SnS layers under 850 nm laser; c) responsivity, and d) detectivity obtained at *P* = 2.5 mW cm^−2^ and *V*_ds_ = 2.0 V at different wavelengths for multiple‐unit cell thick SnS layers; and e) response time of multiple‐unit cell thick SnS layers under 850 nm laser at a frequency of 500 Hz. Reproduced with permission.^[^ ^23^ ^]^ Copyright 2020, Wiley‐VCH. f) *I*–*V* characteristics of the SnS films synthesized with different thicknesses (202 ± 33, 395 ± 17, 480 ± 21, 573 ± 47, 782 ± 35 nm) irradiated from the front side; g) *c* plots of SnS films with a thickness of ≈600 nm under back irradiation and front irradiation; and h) *I*–*V* characteristics of SnS/Pt, SnS/TiO_2_/Pt, and p‐SnS/n‐CdS/n‐TiO_2_/Pt in 0.5 m H_2_SO_4_ under chopped simulated sunlight. Reproduced with permission.^[^ ^78^ ^]^ Copyright 2017, Wiley‐VCH. i) IPCE and absorbance characteristics of SnS nanodisks as a function of photon wavelength; j) prolonged (180 min) *I*–*t* plot obtained from the as‐prepared SnS nanodisk‐based photocathode at −0.3 V versus RHE, and k) its stability at the time intervals of 10,700–10,730 s. Reproduced with permission.^[^ ^215^ ^]^ Copyright 2017, American Chemical Society.

Impressed by the fascinating optoelectronic properties of BP and SnSe,^[^ 8, 209 ^]^ 2D binary IV–VI compound SnS, as a semiconductor material, is of great interest in optoelectronics because its bandgap can be tuned from NIR to visible wavelengths.^[^ 36, 173, 203 ^]^ Moreover, the abundant constituent elements, excellent stability, and low toxicity compared to lead and cadmium, also make SnS‐based nanostructures more promising than most other semiconductors on the basis of long‐term, low‐cost, and environmentally benign optoelectronic technologies.^[^ 23, 25, 60, 78, 87, 103, 139, 210, 211, 212, 213, 214, 215 ^]^ In 2020, Krishnamurthi et al.^[^ ^23^ ^]^ successfully fabricated single‐layer and multilayer SnS with lateral dimensions reaching the millimeter scale by utilizing van del Waals transfer of molten Sn on conventional substrates, such as SiO_2_/Si. **Figure** **21**a presents the schematic diagram of SnS NS‐based photodetector fabricated on a SiO_2_/Si substrate. In this device, separate multilayer SnS layers are set between a pair of Au electrodes. The photoresponse of the multilayer SnS NS‐based photodetectors as a function of the incident power shows a linear increase in photocurrent (*I*) with the irradiation power density (*P*_inc_) (Figure 21b), which can be ascribed to the photoconductive effect. Responsivity (*R*) and detectivity (D*) are two critical parameters which are usually used to evaluate the performance of a photodetector. *R* of a photodetector is calculated based on the equation: *R* = Δ*I*/(*P*_inc_ × *S*), where *S* is the effective area between the two electrodes of the material subjected to irradiation. It can be observed in Figure 21c that the highest *R* of the multilayer SnS NS‐based photodetector can reach up to 3510 A W^−1^ under 850 nm laser with an irradiation intensity of 2.5 mW cm^−2^, and *R*_s_ at other laser wavelengths (*λ*s) range from 10^2^ to 10^3^ A W^−1^, implying the high sensitivity of this multilayer SnS NS‐based photodetector. D* of a photodetector is calculated based on the formula: D* = [(*S* × Δ*f*)^1/2^ × *R*]/*I*_n_, where Δ*f* and *I*_n_ denote the bandwidth and noise current, respectively. The optimal detectivity obtained at a power density of 2.5 mW cm^−2^ for a multilayer SnS‐based device is calculated to be 6.83 × 10^10^ Jones under 850 nm laser (Figure 21d). The response speed at different pulsed frequencies is usually carried to determine the operational speed of a photodetector, which plays an important role in evaluating its feasibility for practical applications. Rise time (*t*_r_) and decay time (*t*_d_) are defined as the time required for the photocurrent to increase from 10% and reach 90% of its peak value and vice versa.^[^ 36, 77 ^]^ The *t*_r_ and *t*_d_ under 850 nm laser and irradiation frequency of 500 Hz are calculated to be 48.33  and 113.14 μs, respectively (Figure 21e), indicating its fast photodetection capability in the NIR region. Moreover, in 2018, Cheng et al.^[^ ^78^ ^]^ used a chemical bath deposition (CBD) technique to directly deposit SnS thin film on the FTO‐coated glass which acted as a photocathode for solar H_2_ production. As shown in Figure 21f, the photoelectrochemical (PEC) performance of the as‐fabricated SnS thin film‐based photocathode with different thicknesses of SnS thin film were measured in the presence of reversible polysulfide redox couple ($S_{2}^{2-}$/HS^−^). The thickness of SnS thin film was easily tuned by simply controlling the CBD time. SnS film with a thickness of ≈600 nm is capable of absorbing incident light with all the wavelengths of less than 1100 nm, and presents a high photocurrent density (12 mA cm^−2^) under front irradiation at 100 mW cm^−2^, which was considered to be the highest photocurrent density for SnS‐based photocathodes in the reported aqueous redox electrolytes. In addition, it can be noted that thickness beyond 600 nm (≈750 nm) has a lower photocurrent density, which could be attributed to carrier transport limitation.^[^ ^78^ ^]^ However, the photocurrent density is significantly lower when the sample was irradiated from the back than from the front (Figure 21g), which could be mainly due to the recombination of a minority of carriers at the interface of semiconductor and electrolyte. Moreover, the optimization of the SnS thin film‐based photocathodes for the PEC H_2_ production was carried out in a N_2_‐purged 0.5 m H_2_SO_4_. The chopped photocurrent density transient (*I*–*t*) at 0 V versus reversible hydrogen electrode (RHE) for bare p‐type SnS (*p*‐SnS) coated with 2 nm electron beam‐deposited Pt NPs can be seen in Figure 21h, where the Pt NPs served as the catalysts for H_2_ evolution. The bare SnS films with Pt NPs display an initial cathodic photocurrent density of 2.9 mA cm^−2^ under 100 mW cm^−2^ irradiation, and the photocurrents substantially decline with the test time, implying that severely chemical and PEC degradation of SnS thin films occurred with the test time under the acidic conditions. To prevent the SnS thin film from degradation, atomic layer deposition was performed to deposit 2 nm‐thick TiO_2_ films on the top of SnS thin film, where TiO_2_ serves as an electron filter because of its large valence band offset.^[^ 216, 217 ^]^ A stable photocurrent density with a maximum value of 0.6 mA cm^−2^ has been achieved after the introduction of the TiO_2_ layer (Figure 21h). It can also be found that a much higher photocurrent density of 2.4 mA cm^−2^ is also obtained after a buffer layer of CdS was inserted between SnS and TiO_2_ layers due to the formation of staggered type II heterojunction.^[^ ^78^ ^]^ Apart from these, in 2017, Patel et al.^[^ ^215^ ^]^ fabricated high‐quality thickness‐controlled SnS thin films on a FTO‐coated glass, which exhibited a significant enhancement of the cathodic photocurrent by a facile thermal treatment. Figure 21i presents the incident photon to current conversion efficiency (IPCE) values of SnS thin film‐based photocathodes measured at an applied potential ranging from 0.1 to −0.3 V versus RHE. The IPCE value can be determined based on the equation: IPCE(*λ*) = (1240 × *J*_ph_)/(*λ* × *P*_ph_), where *J*_ph_ and *P*_ph_ denote the photocurrent density and laser density, respectively, corresponding to the photon wavelength.^[^ ^215^ ^]^ It can be seen in Figure 21i that the IPCE values at all the studied voltages (−0.3, −0.1 and 0.1 V vs RHE) gradually decrease with the increase of photon wavelength, in good accordance with the absorbance result. It should be pointed that at *λ* ≤ 420 nm, the IPCE value (40%) at 0.1 V versus RHE, was enhanced by 2 and 2.4 times at −0.1 and −0.3 V versus RHE, respectively. Furthermore, the long‐term stability of the SnS thin film‐based photocathodes at −0.3 V versus RHE in 0.1 m HCl under pulsed irradiation can be seen in Figure 21j. The photocurrent for the SnS thin film continuously decreases from 7.0 to 4.1 mA cm^−2^ throughout the measurement lasting 3 h (Figure 21k), verifying the excellent stability of the SnS thin film.

### 3.8 Sensors

**Figure 22:**
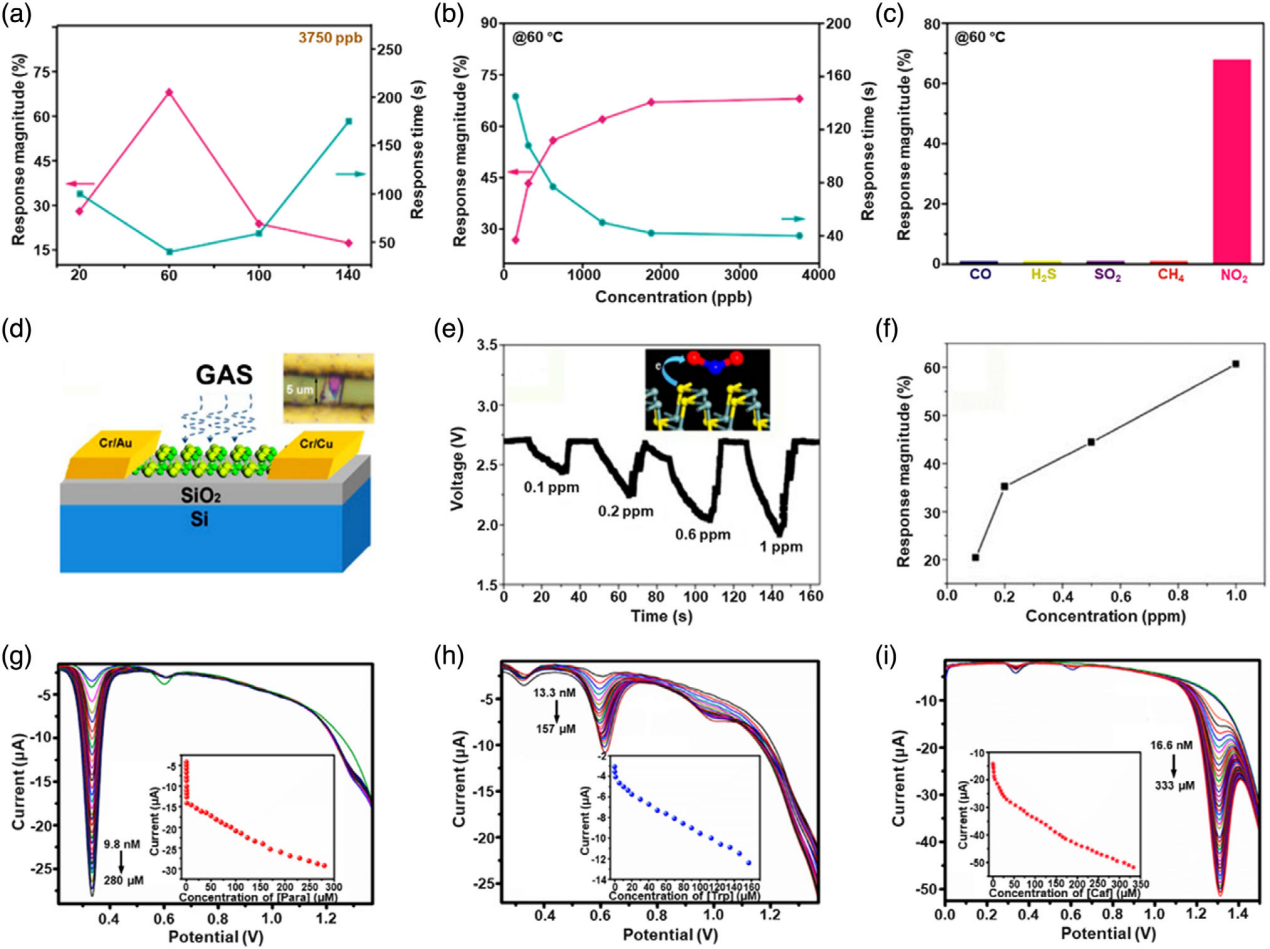
Sensing performance of the SnS‐based nanostructures. a) Response time and response magnitude of the 2D SnS NS‐based sensors upon NO_2_ as a function of operating temperatures; b) response magnitude and response time of the 2D SnS NS‐based sensors as a function of NO_2_ concentration at 60 °C; c) comparison of the response magnitude of CO (300 ppm), CH_4_ (1%), SO_2_ (100 ppm), H_2_S (20 ppm), and NO_2_ (3750 ppb) for 2D SnS NSs at 60 °C. Reproduced with permission.^[^ ^7^ ^]^ Copyright 2019, American Chemical Society. d) Schematic structure of SnS thin‐crystal‐based gas‐sensor device with the optical image of device (inset); e) real‐time voltage response after exposure of device to NO_2_ gas with the increase of NO_2_ concentration; inset showing the electron transfer process from SnS NSs to NO_2_; f) the plot of response as a function of concentration. Reproduced with permission.^[^ ^225^ ^]^ Copyright 2016, American Chemical Society. Differential pulse voltammograms of the effect of g) [Para], h) [Trp] and i) [Caf] at GC‐SnS/TiO_2_@GO electrode; insets showing their corresponding plot of concentration as a function of peak current, respectively. Reproduced with permission.^[^ ^226^ ^]^ Copyright 2019, American Chemical Society.

An ideal sensor usually has advantages of low fabrication costs, excellent sensitivity, a low limit of detection, low hysteresis, a fast and desirably linear response, and preferable recovery for repetitive use.^[^ 56, 98, 218, 219, 220, 221, 222, 223, 224 ^]^ Sensors made from pure SnS nanostructures and SnS‐based composites have shown these properties. For example, 2D p‐type SnS NSs with submicrometer lateral size fabricated by a LPE method were assembled into cost‐effective resistive transducing substrates for a chemical and biological sensor device to detect paramagnetic NO_2_.^[^ ^7^ ^]^ The operation temperature of the 2D SnS NS‐based sensor from room temperature to 140 °C was studied, as shown in **Figure** **22**a. The highest response magnitude (≈68%) and the shortest response time (≈40 s) obtained at 60 °C. The NO_2_ response magnitude and response time of the 2D SnS NS‐based sensor were also investigated in the concentration ranging from 150 per billion (ppb) to 3750 ppb at optimal operating temperature of 60 °C, as shown in Figure 22b. With the increase of NO_2_ concentration, more gas molecules tend to capture photoexcited electrons from the 2D SnS NSs, leading to the enhanced response magnitude and the shorter response time. It is noted that the saturation state is reached at 60 °C when the NO_2_ concentration is greater than 2000 ppb. The as‐fabricated sensor was also exposed to other common gases, such as CO, H_2_S, SO_2_, and CH_4_ for its selectivity measurement, as shown in Figure 22c. It can be seen that all the response magnitudes for CO, H_2_S, SO_2_, and CH_4_ are below ≈1%, which are significantly lower than that of NO_2_ at 60 °C in Figure 22b, possibly due to the relatively strong affinity between NO_2_ gas and 2D SnS NSs. Moreover, large‐size SnS thin crystals were successfully fabricated through 2D oriented attachment (OA) growth of colloidal QDs by a high‐pressure solvothermal method.^[^ ^225^ ^]^ The schematic diagram of the single SnS thin crystal‐based sensor is illustrated in Figure 22d. In brief, thin SnS crystals were firstly dispersed on the surface of a SiO_2_/Si substrate, followed by depositing Cr/Au alloy layer onto the substrate as the electrodes. The dynamic response of the thin SnS crystal‐based device toward NO_2_ shows that with the increase of NO_2_ concentration, the voltage of the device decreases due to the resistance reduction upon exposure to NO_2_ (Figure 22e). Therefore, the higher studied concentration of NO_2_ (0.1–1 ppm) at room temperature, more surface dipoles including surface electric dipoles and magnetic dipoles are generated, leading to more efficient electron transfer from SnS to NO_2_ (Figure 22e inset). The sensor response is nearly linear with the concentration of NO_2_, but the response and recovery time oppositely decrease. The percent change of resistance calculated from Figure 18e as a function of NO_2_ concentration (Figure 22f) verifies that the response of device on exposure to 100 ppb NO_2_ gas at room temperature is around 20% with a very low noise (±5%), suggesting it has significantly superior detection limit. Furthermore, in 2019, Murugan and Kumar^[^ ^226^ ^]^ reported an electrochemical sensor by incorporating SnS microsphere and TiO_2_ on GO NSs (SnS/TiO_2_@GO ternary composite) for concomitant determination of paracetamol ([Para]), caffeine ([Caf]), and tryptophan ([Trp]) in pharmaceutical formulations. The differential pulse voltammetry (DPV) was used to monitor a trace amount of [Para], [Trp], and [Caf] at the SnS/TiO_2_@GO composite modified glassy carbon (GC) electrode (GC‐SnS/TiO_2_@GO), as shown in Figure 22g–i. In the first case, the concentration of [Para] increases from 0.0098 to 280.07 μM while the concentrations of [Trp] and [Caf] keep unchanged. The DPV curves show that peak current (*I*_p_) for [Para] gradually increases with the increasing concentration of [Para] while a slight change in peak current for [Caf] located at around 1.3 eV and [Trp] positioned at around 0.6 V (Figure 22g), indicating strong intermolecular effects between the [Para] and SnS/TiO_2_@GO. There are two linear relationships in the range of 9.8 nM to 10.07 μM (linear regression equation: *I*_p_(10^−6^) = −10.6489[Para μM^−1^]−4.1299 (*R* ^2^ = 0.9929)) and 20.34 to 280.07 μM (linear regression equation: *I*_p_(10^−6^) = −0.0557[Para μM^−1^]−14.7830 (*R* ^2^ = 0.9831)), as seen in the inset of Figure 18g. Similarly, the *I*_p_s for [Trp] and [Caf] also show nearly linear increase with the correspondingly increasing concentration of [Trp] and [Caf], respectively, as shown in Figure 22h,i. The detection limits are 7.5, 7.8, and 4.4 nM for [Para], [Trp], and [Caf], respectively. It was demonstrated that the as‐prepared GC‐SnS/TiO_2_@GO electrode showed significantly superior sensing ability, relatively wide linear range, distinctly low detection limit, and significant decrease in overpotential due to its larger surface area and higher electrical conductivity.^[^ ^226^ ^]^ The SnS/TiO_2_@GO ternary composite electrode was also proved to enable a facile preparation of commercial sensors for drug detection with a high sensitivity.

### 3.9 Ferroelectrics

**Figure 23:**
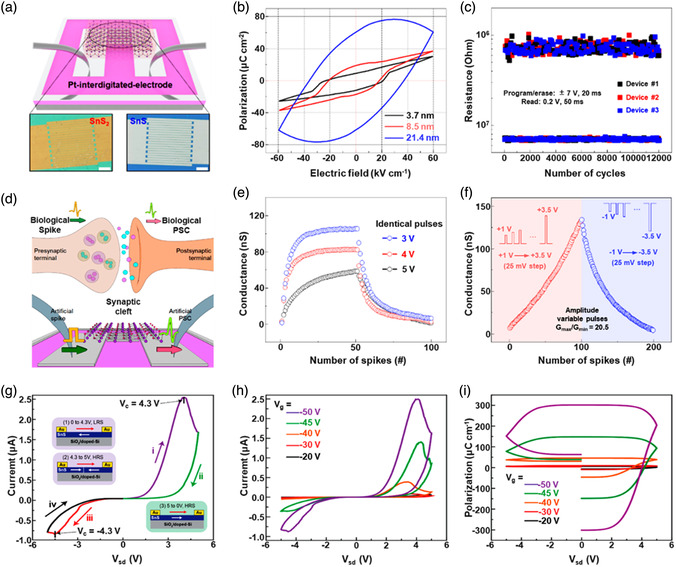
Ferroelectric properties of the SnS‐based nanostructures. a) Schematic illustration of the CVD‐grown SnS thin film‐based device; b) polarization‐electric field curves of a SnS thin film with different thicknesses and the frequency was fixed at 500 Hz (2 ms bipolar sweep); c) resistance changes against applied pulse (fatigue test) of a 3.7 nm‐thick SnS thin film on a Pt‐interdigitated electrode substrate; d) schematic illustration of comparison between biological (above) and artificial synapses (below); e) Postsynaptic current changes as a function of the number of spikes with different pulse amplitudes (from ±3 to ±5 V) and fixed width of 20 ms; and f) conductance changes in potentiation and depression of the fabricated device with an incremental pulse scheme (+1.0 V to +3.5 V, 25 mV steps, 100 states); all PSCs were collected with a read voltage of 0.1 V at the postsynaptic neuron. Reproduced with permission.^[^ ^175^ ^]^ Copyright 2020, American Chemical Society. g) *I*–*V* hysteresis curve of a SnS‐based FET device (channel length *L* = 4 μm, cycled from −5 to 5 V); the voltages were swept in the order 0 → 5 → 0 → −5 → 0 V, as shown by the colored arrows with the four sweeps labeled as i, ii, iii, and iv; inset: schematic illustration of the ferroelectric switching processes during the voltage sweeps i (processes 1 and 2) and ii (process 3); h) *I*–*V* hysteresis curves of a lateral SnS‐based memory device (channel length *L* = 4 μm, cycled from −5.5 to +5.5 V) measured at different *V*_g_s; and i) corresponding *P*–*V*_sd_ hysteresis curves of the memory device at different *V*_g_s. Reproduced with permission.^[^ ^76^ ^]^ Copyright 2019, American Chemical Society.

Ferroelectrics, with spontaneous switchable electric polarization, are an important branch of functional materials owing to their broad applications in many fields, such as random‐access memory, field‐effect transistors (FET), photovoltaics, etc.^[^ 227, 228, 229, 230 ^]^ Nowadays, ultrathin ferroelectrics have received extensive attention for miniaturized sensors, optoelectronic devices, etc., due to its low power consumption and compatible integration for high‐density device.^[^ 12, 231, 232, 233, 234, 235 ^]^ Phosphorene and phosphorene analogues with anisotropic structures, high carrier mobility, thickness‐dependent bandgap, etc., such as SnS, hold great promises in the application of ferroelectricity and ferroelasticity.^[^ 85, 86, 162 ^]^ In 2020, Kwon et al.^[^ ^175^ ^]^ successfully fabricated a lateral Pt/SnS/Pt‐based artificial synapse using ultrathin polycrystalline SnS films grown by a CVD technique, which exhibited stronger ferroelectric response and higher pattern recognition accuracy based on artificial neural network (ANN) simulation. The schematic diagram of the CVD‐grown SnS thin film‐based device can be seen in **Figure** **23**a. Different from SnS_2_ with no obvious ferroelectric property, the shape of the polarization‐electric field curves for SnS thin film gradually tends to be a circle with the increase of SnS thickness (Figure 23b), in good accordance with thickness‐dependent *I–V* results,^[^ ^175^ ^]^ and the CVD‐fabricated SnS with a thickness of 21.4 nm is likely to be a resistor semiconductor. The fatigue measurement of the as‐fabricated device was carried out to evaluate the nonvolatile storage performance and ferroelectric endurance properties, as shown in Figure 23c. The result shows that all the three as‐fabricated 3.7 nm‐thick SnS samples have excellent stability with an on/off ration of ≈10, suggesting that the CVD‐fabricated SnS holds great potential for ferroelectric memory in practical applications. Moreover, the schematic diagram of the comparison between electronic and biological synapses using Pt/SnS/Pt structure can be seen in Figure 23d. The postsynaptic current (PSC), known as the synaptic strength contributing to the storage of information, was collected when a cluster of 50 identical continuous potentiation/depression spikes were applied at various pulse amplitudes, as shown in Figure 23e. There are partial switchable ferroelectric domains observed at ±3 V because the PSC at ±3 V still keeps increased/decreased after 50 potentiation/depression pulses, and they can be completely switched at either ±4 or ±5 V of identical spikes due to the attainable saturated state. To obtain a high value of maximum conductance (*G*_max_)/minimum conductance (*G*_min_) ratio and linear long‐term plasticity (LTP/LTD) curve, a continuously increased voltage spike was applied, as seen in Figure 23f. It should be noted that *G*_max_/*G*_min_ is an important parameter related to mapping capability and power consumption of an efficient neural network, and linear LTP/LTD curve is used to calculate the accuracy of recognition in neural network simulation. The as‐fabricated Pt/SnS/Pt device displays a higher conductance ratio (20.5) for precise learning ability and more conductance states for better learning capability and enhanced robustness, due to its relatively lower operation voltage and much stronger ferroelectric properties, compared to conventional ferroelectric oxides.^[^ ^236^ ^]^ In addition, in 2019, Bao et al.,^[^ ^76^ ^]^ reported robust in‐plane ferroelectricity in large‐area, high‐quality, 2D few‐layer SnS fabricated by a molecular beam epitaxy technique on a variety of substrates that was coupled anisotropically to lattice strain, which revealed strong ferroelectric response at negative gate voltages. The ferroelectric field‐effect transistor (FeFET) based on 2D few‐layer SnS was assembled to investigate the switching and gate tunability of in‐plane ferroelectricity. Figure 23g presents a representative *I*–*V* curve for the FeFET device in the source‐drain bias (*V*_sd_) from +5 to −5 V at an applied back‐gate voltage of −50 V. The arrows in Figure 19g represent the voltage sweep directions. It can be found that with the increasing bias from 0 to +4.3 V (Figure 23g, sweep i), the device displays a low‐resistance state (LRS) due to a negative polarization from the last negative bias sweep (Figure 23g, inset 1), and when the bias exceeds +4.3 V, the negative polarization of SnS domains reverses to be positive, making the few‐layer SnS‐based FeFET device reach a high‐resistance state (HRS) with a sharp current decrease (Figure 23g, inset 2). When the bias gradually decreases to 0 V, the few‐layer SnS‐based FeFET device keeps stayed in the HRS (Figure 23g, sweep ii) and the polarization has the same direction as the external field (Figure 23g, inset 3). Therefore, the coercive bias is considered to be ±4.3 V, which is corresponding to a coercive filed of ≈10.7 kV cm^−1^, close to those of conventional ferroelectrics.^[^ 237, 238 ^]^ The *I*–*V* hysteresis behaviors were also collected as a function of gate voltage to evaluate the electrostatical tunability of the ferroelectricity in few‐layer SnS, as shown in Figure 23h. Upon applying a positive gate voltage (*V*_g_), *e* ^−^ enters into the *p*‐SnS,^[^ ^161^ ^]^ resulting into carrier depletion and significant current drop, while the *I*–*V* hysteresis curves display obvious current peaks at negative *V*_g_, which matches with the ferroelectric switching. Considering that the *V*_sd_ was swept at 1 V s^−1^, the polarization (*P*) can be calculated by the equation: *P*(V) = *d* ^−1^∫*I*(V)dV, where *d* is the distance between the drain electrodes and source. The *P*–*V*_sd_ curves show that the remnant polarization increases with the negative gate voltage, and the discontinuous switching characteristics in *P*–*V*_sd_ curves is similar to that of a diode (Figure 23i), which can be mainly attributed to the asymmetric Schottky barriers at the interface of Au and SnS, and inhomogeneous doping. It was manifested that the few‐layer SnS‐based FeFET device has great promise in the field‐effect‐controlled memory devices as well as nanoscale electromechanical systems. Moreover, in 2021, Sutter et al.,^[^ ^86^ ^]^ employed piezoresponse force microscopy to investigate the in‐plane ferroelectric polarization arrangement and layer‐to‐layer polar ordering in the SnS NSs, which exhibited stacking‐dependent polarization and in‐plane ferroelectric domain structures that include stripe domains and charged domain walls.

### 3.10 Thermoelectrics

**Figure 24:**
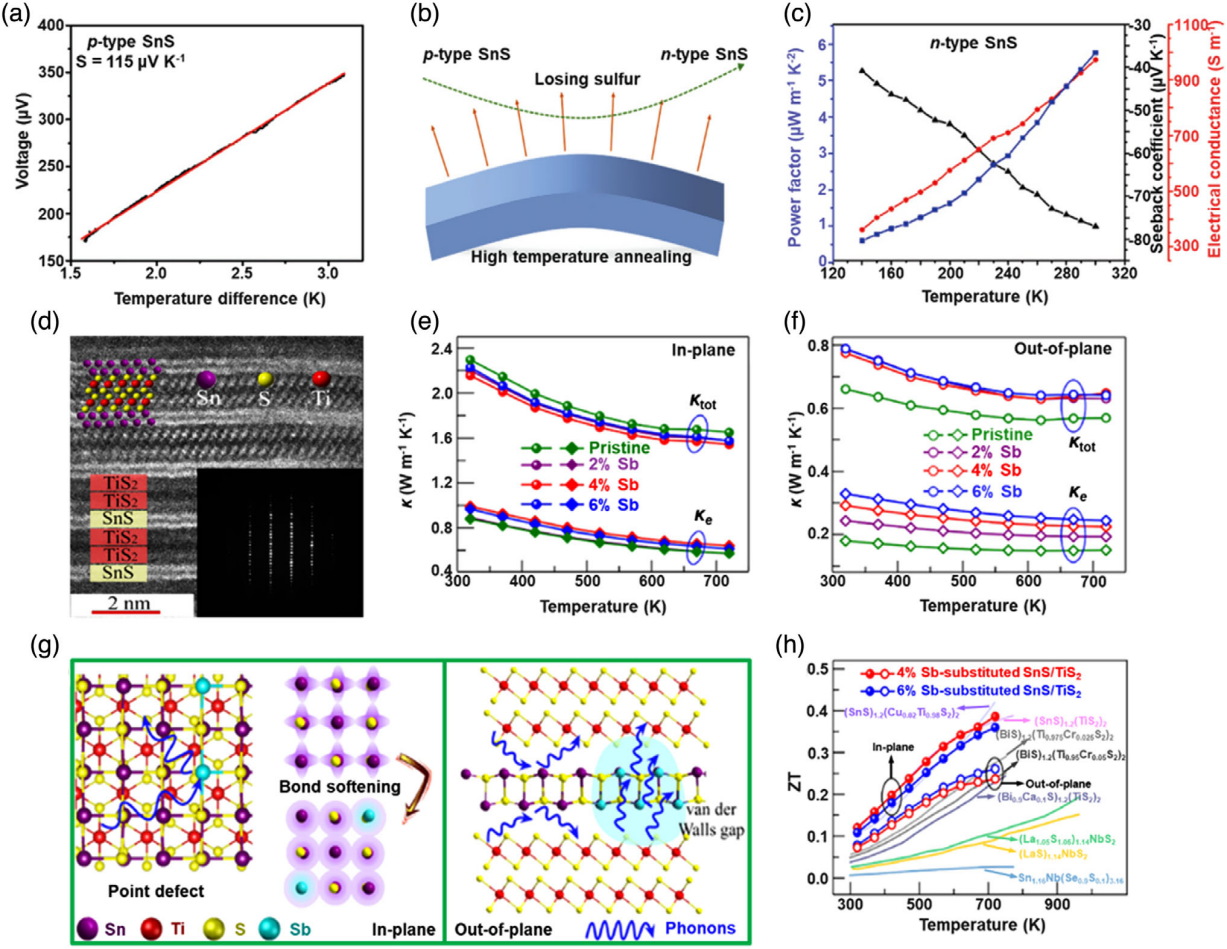
Thermoelectric properties of the SnS‐based nanostructures. a) Schematic illustration of high‐temperature annealing and the transition of the SnS from p‐type to n‐type; b) room temperature Seebeck coefficients of the p‐type SnS thin film; and c) temperature‐dependent Seebeck coefficients, electrical conductivity, and power factor of the n‐type SnS thin film. Reproduced with permission.^[^ ^250^ ^]^ Copyright 2018, Wiley‐VCH. d) Atom‐resolved HAADF‐STEM image for the pristine SnS/TiS_2_ composites fabricated by a conventional solid‐state reaction method; e) temperature dependence of total thermal conductivity *κ*_tot_ and electronic thermal conductivity *κ*_e_ in the in‐plane direction for Sb‐substituted SnS/TiS_2_ composites with different Sb loadings; f) out‐of‐plane direction *κ*_tot_ and *κ*_e_ for these composites; g) schematic representation of the phonon transport in the in plane (left) and out of plane (right) of Sb‐substituted SnS/TiS_2_ composites; and h) temperature dependence of ZT in comparison with the state‐of‐the‐art misfit‐layered chalcogenide. Reproduced with permission.^[^ ^239^ ^]^ Copyright 2019, American Chemical Society.

Thermoelectric (TE) materials can make it come true for the effective conversion between electric and thermal energies by virtue of thermoelectric effect.^[^ 9, 10, 11, 14, 104, 164, 239, 240 ^]^ Harvesting electricity by utilizing waste heat is a clean and sustainable approach to overcome the challenge of the traditional fuel resource consumption, making thermoelectrics become a research hotspot with remarkable attentions for extensive application prospects.^[^ 241, 242, 243, 244, 245, 246, 247 ^]^ The conversion efficiency of a TE material is usually dependent on the dimensionless figure of merit (ZT), defined as ZT = (*S* ^2^ *σ*/*κ*)*T*, where *S* is Seebeck coefficient, *σ* is electrical conductivity, and *κ* is total thermal conductivity including the lattice part (*κ*_L_) and the electronic part (*κ*_e_). SnS with an electronic band structure exhibits low thermal conductivity, favorable transport properties, and the controlled carrier concentration by simply extrinsic doping, making it a promising candidate in the community of thermoelectric power generation.^[^ 240, 242, 248 ^]^ To further improve the TE performance, nanoengineering approaches, such as doping SnS with heteroatoms or alloying SnS with metals or metal chalcogenides,^[^ 104, 164, 239, 240, 242, 249, 250 ^]^ provide an alternative way to optimize the ZT of SnS‐based nanostructures for next‐generation thermoelectrics. For example, in 2018, Yin et al.^[^ ^250^ ^]^ reported successful synthesis of SnS nanocrystals and applied the flexible SnS thin films for high‐performance flexible thermoelectrics. The temperature‐dependent TE performance of the SnS thin film was characterized, as shown in **Figure** **24**a. The result shows that the S of the as‐fabricated SnS thin film exhibits a value of 115 μV K^−1^ at room temperature, indicating that the as‐fabricated SnS nanocrystals are p‐type semiconductors. However, an excess of ligands in the fabrication process made the *σ* of the as‐fabricated SnS nanocrystals relatively poor (less than 100 S m^−1^).^[^ ^250^ ^]^ To efficiently enhance the *σ*, the SnS thin film was annealed at 500 °C under N_2_ to completely get rid of the ligands. Surprisingly, the annealing treatment at 500 °C not only contributed to a large improvement in *σ*, but also made a transition of *p*‐SnS to *n*‐SnS (Figure 24b). As for the annealed SnS thin film, the *σ* increases from 360 to 972 S m^−1^ as the temperature rises from 140 to 300 K (Figure 24c), revealing representative semiconductor behavior. Meanwhile, S displays a nearly linear decrease from −40.8 to −77 μV K^−1^ with an improved PF of 5.8 μW m^−1^ K^−2^ at room temperature. The mechanism of the transition of *p*‐SnS to *n*‐SnS is illustrated, as shown in Figure 24b. In the annealing process, anion atoms in SnS are partially lost, making S deficient (Sn self‐doping), and thereby Sn acts as an electron donor, leading to the *n*‐SnS film. Additionally, in 2019, polycrystalline misfit‐layered antimony (Sb)‐substituted SnS/TiS_2_ alloys were successfully synthesized by a solid‐state reaction method and systematically investigated for their anisotropically thermoelectric properties in a wide temperature range (320–720 K).^[^ ^239^ ^]^ The HAADF‐STEM image and SAED pattern confirm the layered structure of the misfit‐layered superlattice, where a single SnS layer and double TiS_2_ layers are stacked alternatively along the *c*‐axis (Figure 24d). The TiS_2_ layers separated by van der Waals act as conductive species while the SnS layer can be regarded as a barrier layer to suppress phonon transmission. The temperature dependence of the total thermal conductivity (*κ*_tot_) and electronic thermal conductivity (*κ*_e_) in the both directions of in‐plane and out‐of‐plane were investigated, as shown in Figure 24e,f. Notably, both in‐plane *κ*_tot_ and *κ*_e_ are significantly higher than those of out‐of‐plane ones, respectively. Interestingly, the substitution Sb in the SnS/TiS_2_ alloys can efficiently improve the thermoelectric performance in both directions. With respect to the lattice thermal conductivity (*κ*_l_), it sharply decreases in the entirely studied temperature range with the increase of Sb content, especially for the sample with 4% Sb loading, mainly due to the point defects in the process of Sb substitution. In addition, the in‐plane transverse sound velocity after Sb substitution shapely reduces, suggesting the apparent weakness of atomic bonding.^[^ ^239^ ^]^ Both point defects and softened transverse sound velocity were considered to make a great contribution to the overall decline of in‐plane *κ*_l_ (Figure 24g). It should be pointed out that the ZTs for all the Sb‐substituted samples have a comprehensive improvement in both directions and the in‐plane ZTs are apparently higher than those of out‐of‐plane ones under the same conditions (Figure 24h). Notably, the in‐plane ZT for the 6% Sb‐substituted sample or even the out‐of‐plane ZT for 6% Sb‐substituted one is superior/comparable to currently state‐of‐the‐art misfit‐layered chalcogenides, such as Cu‐substituted (SnS)_1.2_/(TiS_2_)_2_ composites,^[^ ^251^ ^]^ (SnS)_1.2_/(TiS_2_)_2_ composites,^[^ ^252^ ^]^ (BiS)_1.2_/(TiS_2_)_2_ composites,^[^ 253, 254 ^]^ [(LaS)_*x*_]_1.14_NbS_2_ composites,^[^ ^255^ ^]^ etc. The texturization engineering provides a new guidance on the improvement of the thermoelectric performance in misfit‐layered chalcogenides.

### 3.11 Nonlinear Properties

**Figure 25:**
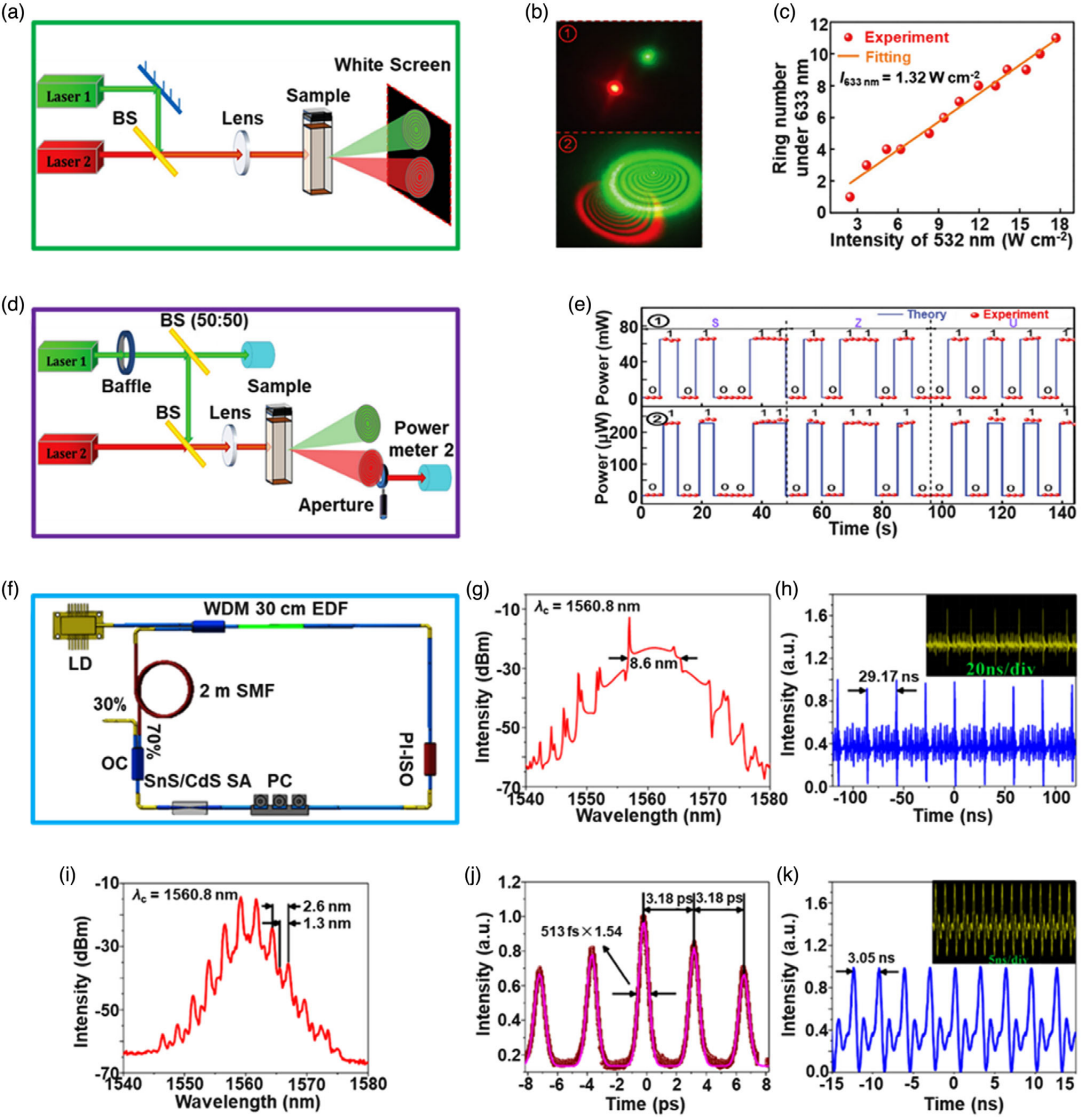
Nonlinear properties of the SnS‐based nanostructures. a) Schematic of the experimental configuration for all‐optical switching based on few‐layer SnS; b) the actual images obtained from the experiment; c) ring number under 633 nm laser with a relatively weak laser density as a function of a strong laser intensity of 532 nm; d) schematic of the experimental configuration for the information carrier based on few‐layer SnS; e) The input signal of the ASCII code of “SZU” (➀), and the corresponding output signal (➁). Reproduced with permission.^[^ ^173^ ^]^ Copyright 2017, Wiley‐VCH. f) Experimental setup of the erbium‐doped fiber laser based on SnS/CdS saturated absorber; g) traditional soliton mode‐locked spectrum with dip‐type sidebands; h) oscilloscope trace; i) ninth bound‐state harmonic mode‐locked spectrum based on SnS/CdS saturated absorber; j) autocorrelation trace of the ninth bound‐state harmonic mode‐locked pulse; and k) oscilloscope trace of the ninth bound‐state harmonic mode‐locked pulse train. Reproduced with permission.^[^ ^257^ ^]^ Copyright 2020, American Chemical Society.

Over the past five years, research on the interaction of SnS‐based nanostructures with photons has attracted great attention in nanophotonic applications, such as optical modulators, and nonlinear photonic devices.^[^ 2, 173, 176, 256, 257, 258 ^]^ Theoretical investigations showed a sizeable bandgap, high carrier mobility, large absorption coefficient, and odd–even quantum confinement effect in 2D SnS.^[^ 169, 176 ^]^ Experimentally, a variety of SnS‐based nanostructures were employed to achieve the advanced photonic performance, such as 2D SnS NSs,^[^ 2, 173, 176, 256 ^]^ SnS thin films,^[^ ^258^ ^]^ and SnS/CdS nanoflower heterostructures.^[^ ^257^ ^]^ In 2017, our group^[^ ^173^ ^]^ successfully fabricated BPA SnS NSs by a facile LPE method, demonstrating excellent nonlinear optical response and stability. The light‐control‐light technique was used to modulate/manipulate the nonlinear phase change based on the spatial cross‐phase modulation (SXPM) effect. The schematic illustration of the experimental setup for all‐optical switching can be seen in **Figure** **25**a. One laser beam with a high intensity mixed together with another laser beam with a relatively weak intensity by a beam splitter (BS) to propagate both laser beams, and the diffraction rings in the screen behind can be observed. The experimental results show that the 633 nm laser with a lower intensity than the threshold for the diffraction ring excitation cannot generate the linear optical effect (red spot in upper subfigure in Figure 25b) while another controlling 532 nm laser with a laser density of 2.45 W cm^−2^ only can excite one diffraction ring (green spot in upper subfigure of Figure 25b). Note that the number of diffraction rings for both 532 and 633 nm lasers can be efficiently enhanced with the incremental laser density (lower subfigure of Figure 25b). The relationship of the diffraction between the two lasers illustrates that the number of diffraction rings under 633 nm laser is almost proportional to the 532 nm laser density (Figure 25c), indicating that the phase of the signal laser (633 nm laser) has an obvious change due to the SXPM. Furthermore, a light–control–light technique was employed to realize all‐optical information conversion, that is, information conversion from one laser wavelength channel to another. The configuration for the information carrier based on few‐layer SnS can be seen in Figure 25d. Upon passing through the BS, the laser is divided into two parts with the same optical power. One part is monitored by power meter 1 as a reference while another part is used as a signal laser to determine the diffraction rings based on the aforementioned discussion. As the baffle is opened, the diffraction rings under signal laser are retransmitted and detected by power meter 2, which can be used for information transmission and conversion.^[^ ^173^ ^]^ A typical example of transmitting information based on this effect can be seen in Figure 25e. By simply controlling the baffle, an input signal “01010011 01011010 01010101” corresponding to the ASCII code of “SZU” of the controlling laser can be successfully transmitted, which sheds new light on the optical communication technology. In addition, in 2020, SnS/CdS nanoflower heterostructures synthesized by a water bath method was used to study the nonlinear optical properties.^[^ ^257^ ^]^ Here, the SnS was employed as a saturated absorber owing to its strong NIR light absorption capacity and CdS was used to modulate charge behavior due to its excellent pyroelectric effect. The experimental configuration of the fiber laser based on the SnS/CdS saturated absorber can be seen in Figure 25f. This mode‐locked spectrum indicates that the central wavelength and 3‐dB bandwidth are 1560.8 and 8.6 nm, respectively (Figure 25g). The dip‐type sideband can also be observed at a wavelength of ≈1570 nm, which can be mainly attributed to a four‐wave mixing type in the parametric process between the dispersive waves and solitons. The pulse train shows that the pulse interval is 29.17 ns, and the corresponding cavity length is 5.7 m (Figure 25h). The ninth harmonic soliton molecule can be easily realized by properly tuning the polarization controller (PC) at a pump power of 351 mW. A modulation period of 2.6 nm and a central wavelength of 1560.8 nm are obtained in the mode‐locked spectrum (Figure 25i). The autocorrelation trace of the ninth harmonic soliton molecule can be seen in Figure 25j. The pulse duration and pulse–pulse separation are 513 and 3.18 ps, respectively. The oscilloscope trace of the pulse train in Figure 25k shows that the pulse interval is 3.05 ns, and the corresponding repetition rate is 328 MHz. It was anticipated that the outstanding SnS/CdS nanoflower materials could provide a fundamental guidance on the advanced nanophotonic devices, such as all‐optical modulators, all‐optical switching devices, and photodetectors.

### 3.12 Biomedical Applications

**Figure 26:**
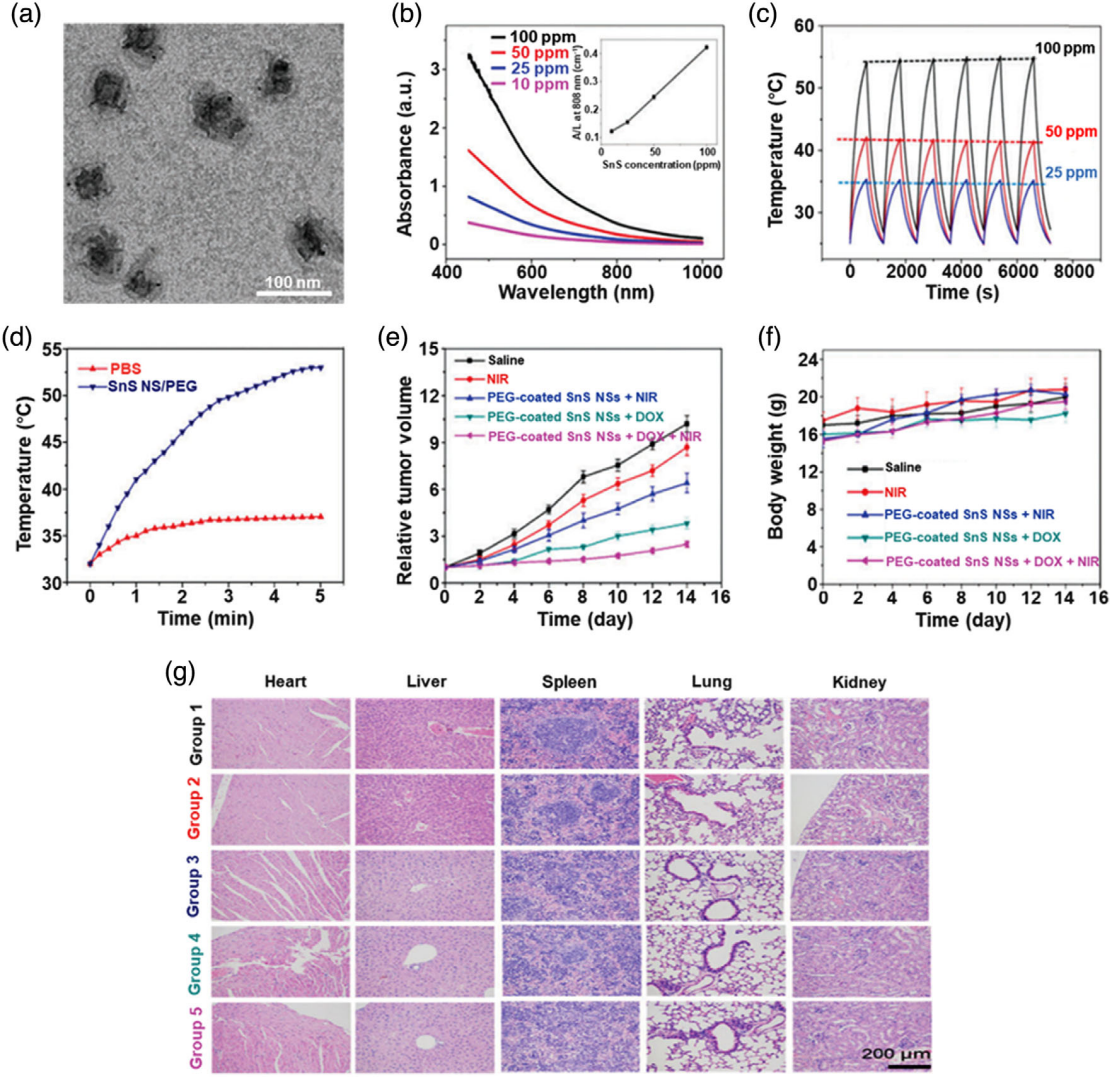
Biomedical properties of SnS‐based nanostructures. a) TEM image of SnS NSs coated by PEG; b) absorption spectra of SnS NSs dispersed in water at different concentrations; inset shows the normalized absorbance intensity divided by the characteristic length of the cuvette (*A*/*L*) at different concentrations under 808 nm laser; c) photothermal heating curves of SnS NSs dispersed in water at different concentrations under irradiation under 808 nm laser for six cycles, and the dotted lines shows the relatively stable photothermal temperature change of the as‐prepared SnS NSs; d) the change of the SMCC‐7721 tumor temperature in mice during laser irradiation; e) corresponding growth curves and representative photographs of tumors removed from the euthanized nude mice treated with saline solution, laser irradiation only, PEG‐coated SnS NSs with laser irradiation, PEG‐coated SnS NSs + DOX without laser irradiation and PEG‐coated SnS NSs + DOX with laser irradiation; f) body weight of nude mice taken every other day after various treatments; and g) H&E‐stained images of major organs from the different groups. Reproduced with permission.^[^ ^203^ ^]^ Copyright 2018, The Royal Society of Chemistry.

In recent years, metal sulfides have received extensive attention in biomedical applications due to their fascinating properties, such as low‐cost, suitable bandgap, environmentally high stability, etc.^[^ 24, 26, 203, 259, 260, 261, 262, 263, 264 ^]^ SnS is also biocompatible and nontoxic due to its poor absorption by human body and rapid excretion in faeces.^[^ 203, 265 ^]^ Up to now, 0D SnS QDs, 2D SnS NSs, and triangular SnS nanopyramids were used in biomedical applications such as photothermal therapy, chemo‐therapy agents, and photoacoustic imaging.^[^ 24, 26, 203 ^]^ In 2018, our group used 2D SnS NSs‐based dual therapy nanoplatforms to achieve excellent tumor therapy by a combination of photothermal‐ and chemo‐therapy.^[^ ^203^ ^]^ The 2D SnS NSs were successfully fabricated by a LPE method and were then functionalized with positively charged poly(ethylene glycol) amine (PEG‐NH_2_) by electrostatic interaction, which was characterized by TEM image with apparent halo coating of PEG on the surface of the 2D SnS NSs (**Figure** **26**a). The optical absorption of the 2D SnS NSs dispersed in water shows a broad absorption ranging from the UV to NIR regions (Figure 26b) and the extinction coefficient at 808 nm is measured to be 3.4 L g^−1^ cm^−1^ based on the Lambert–Beer law (Figure 26b inset). The photothermal property of the 2D SnS NSs at different concentrations in water under 808 nm laser demonstrates that the temperature increases with the concentration and can reach 54 °C at a SnS NS concentration of 100 ppm (Figure 26c), manifesting that the as‐fabricated 2D SnS NSs have excellent photothermal effect. The as‐prepared 2D SnS NSs were employed as carriers for loading doxorubicin (DOX), a prevalent anticancer drug in the clinical application. In in vitro experiments, 2D SnS NSs‐based dual therapy nanoplatforms showed an enhanced therapeutic effect, which was confirmed by an in vivo antitumor evaluation. After the intratumoral injection of PBS and PEG‐coated SnS NSs, it can be observed that the temperature increase (Δ*T*) of PBS‐treated mice is only about 5 °C while that of PEG‐coated SnS NS‐treated mice is up to 23 °C (Figure 26d), confirming its excellent photothermal efficiency of the dual therapy nanoplatform in vivo. Based on this, an in vivo antitumor evaluation was carried out to verify the improved cancer therapy. The SMCC‐7721 (human hepatocellular carcinoma) tumor‐bearing nude mice was treated as follows: group 1, with saline (control); group 2, with laser irradiation only; group 3: PEG‐coated SnS NSs with laser irradiation; group 4: PEG‐coated SnS NSs + DOX without laser irradiation; and group 5: PEG‐coated SnS NSs + DOX with laser irradiation. The tumor volumes (width and length) were measured every two days. After the experiment, all nude mice were euthanized and their tumors were collected. It can be seen in Figure 26e that there is a significantly enhanced therapeutic effect for group 5 compared to other cases, indicating that an improved antitumor effect could be realized by this dual therapy strategy (photothermal‐ and chemo‐therapy). Moreover, the body weight of the nude mice has not been dramatically affected after the dual treatment (Figure 26f), verifying that there are no acute side effects in the process. The major organs of the mice were dissected and stained using hematoxylin and eosin (H&E) for histological analysis. The mice after treated by group 5 (PEG‐coated SnS NSs + DOX with laser irradiation) exhibits no noticeable damage to normal tissue (Figure 26g **)**, indicating that the combined treatment has no dramatic side effects or toxicity to the healthy tissues. Because of the facile fabrication, lower power laser irradiation with precise treatment, negligible side effects, high photothermal efficiency, and compatibility, it was expected that the dual or even multiple therapy nanoplatforms based on nanostructures would pave a new way to the precise treatment of cancer in the future.^[^ ^203^ ^]^

## 4 Conclusion and Perspectives

This comprehensive review provides an overview of nanoengineering of SnS‐based nanostructures (0D, 1D, 2D, or 3D pure SnS nanostructures, and SnS‐based loaded, sandwiched, or encapsulated model) and is further exampled to the detailed illustration of the diverse applications (batteries and solar cells, catalysis, optoelectronics, sensors, ferroelectrics, thermoelectrics, nonlinear properties, and biomedical applications) of SnS‐based nanostructures, highlighting the great promise of SnS‐based nanostructures in the next‐generation devices. Although the great progress has been achieved on the SnS‐based nanostructures, yet there is still much work stayed at the infant stage and some hurdles have to be urgently resolved. 1) With regard to the synthesis, the mass production and process integration of the SnS‐based nanostructures are very crucial to the industrial applications but still difficult to realize. Therefore, it is vital to develop simple, cost‐effective, and eco‐friendly synthetic approaches to controlled synthesis of the high‐quality and well‐defined SnS‐based nanostructures on a large scale, and further develop facile strategies to efficiently control grains and grain boundaries or single crystalline film growth over large areas of SnS to largely boost the practical applications in modern nanodevices. 2) In the field of batteries and solar cells, although SnS nanostructures have achieved a rapid development, they are still greatly limited by many disadvantages, such as low electrical conductivity, poor capacity retention, and volume expansion during alloying/dealloying. Some effective strategies, such as elaborate design of SnS‐based nanostructures, the introduction of carbonaceous materials (i.e., amorphous C, biochar) and 2D Xenes (i.e., BP, bismuthene, antimonene, and graphdiyne), and nanoengineering techniques (i.e., doping, hydrothermal/solvothermal approach, and CVD) are allowed for further development of high‐performance SnS‐based energy devices. 3) As for catalysis, the mechanism of SnS‐based nanostructures is still unclear mainly due to the insufficiently precise nanostructures and absence of in situ characterization. Therefore, it is imperative to precisely synthesize the SnS‐based nanostructures and in situ characterize the structure–property relationships of SnS‐based nanostructures to deeply understand the catalytic mechanism. 4) In spite of the rapid development in the field of optoelectronics and sensors based on SnS‐based nanostructures, the related performances (e.g., detectivity and sensitivity) are still needed to be largely improved for practical applications. In addition, it is essential to develop high‐performance optoelectronics and sensors with unique features, such as super‐small size, ultra‐light mass, flexibility, extreme sensitivity, energy efficiency, and intelligence to meet the urgent demand of modern nanodevices. 5) With respect to ferroelectronics and thermoelectrics, intrinsic local lattice defects, and impurities induced by the solution (hydrothermal/solvothermal) method in the synthetic process of SnS‐based nanostructures, severely scatter the carriers resulting into unsatisfactory results (e.g., poor retention and response in ferroelectronics, and reduced μ and low *σ* in thermoelectrics). Therefore, CVD, PVD, or other methods with relatively less defects and better quality should be preferentially taken into consideration in the high‐performance SnS‐based thermoelectrics. 6) Multifunctionalities based on the elaborate design of SnS‐based nanostructures, a combination of individual functionalities, such as the synergistic effect of photothermal and chemo‐therapy, and a marriage of photodetection and photocatalysis, etc., is an emerging field for multidisciplinary applications due to the inherently multiple properties. The rapid development of multidisciplinary applications based on SnS‐based nanostructures is of great significance for overall improvement of the next‐generation multifunctional devices.

It is envisaged that the hot research of the functional SnS‐based nanostructures will continue to be extensively expanded. The current explorations and insights from the related frontier research such as batteries, solar cells, sensors, catalysis, etc. are of primary importance, with broad implications not only in the emerging BPA materials and 2D Xenes, but also in the high‐performance devices with modern features (intelligence, flexibility, stretchability, ultra‐sensitivity, etc.).

## Conflict of Interest

The authors declare no conflict of interest.
